# Design, synthesis, antiproliferative assessments, and computational studies of new quinolin-2(1*H*)-ones as dual EGFR/HER-2 inhibitors

**DOI:** 10.3389/fchem.2025.1638489

**Published:** 2025-09-16

**Authors:** Lamya H. Al-Wahaibi, Hesham A. Abou-Zied, Martin Nieger, Stefan Bräse, Bahaa G. M. Youssif, Hendawy N. Tawfeek

**Affiliations:** 1 Department of Chemistry, College of Sciences, Princess Nourah bint Abdulrahman University, Riyadh, Saudi Arabia; 2 Medicinal Chemistry Department, Faculty of Pharmacy, Deraya University, Minia, Egypt; 3 Department of Chemistry, University of Helsinki, Helsinki, Finland; 4 Institute of Biological and Chemical Systems, IBCS-FMS, Karlsruhe Institute of Technology, Karlsruhe, Germany; 5 Department of Pharmaceutical Organic Chemistry, Faculty of Pharmacy, Assiut University, Assiut, Egypt; 6 Chemistry Department, Faculty of Science, Minia University, El Minia, Egypt; 7 Unit of Occupational of Safety and Health, Administration Office of Minia University, El-Minia, Egypt

**Keywords:** quinoline, kinases, X-ray, DFT, anticancer, EGFR, HER-2

## Abstract

**Introduction:**

A novel series of quinolin‐2(1*H*)‐one derivative was rationally designed, synthesized, and characterized as potential dual inhibitors of EGFR and HER-2.

**Methods:**

Structural elucidation was achieved through IR, NMR, mass spectrometry, elemental analysis, and single‐crystal X‐ray crystallography. The synthesized compounds were screened for antiproliferative activity against four human cancer cell lines.

**Results and Discussion:**

Compound **5a** exhibited the most potent antiproliferative profile, particularly against MCF‐7 breast cancer cells (IC_50_ = 34 nM), outperforming erlotinib (IC_50_ = 40 nM). Kinase inhibition assays further confirmed dual activity of **5a**, with IC_50_ values of 87?nM and 33?nM against EGFR and HER‐2, respectively. Compound **5a** induced apoptosis via activation of caspase‐3, ‐8, and ‐9, along with upregulation of Bax, downregulation of Bcl-2, and increased cytochrome c release. Flow cytometry analysis demonstrated that **5a** caused significant G0/G1 phase arrest in MCF‐7 cells, indicating a cytostatic mechanism of action. Computational studies provided structural validation of the observed biological activities. Molecular docking studies showed a strong binding affinity **5a** within the ATP‐binding pockets of EGFR and HER‐2, supported by key hydrogen bonding and hydrophobic interactions. These findings were further corroborated by 100 ns molecular dynamics simulations, which confirmed the structural stability and compactness of the **5a**-HER-2 complex, as evidenced by low RMSD, consistent RMSF, and favorable radius of gyration and potential energy profiles. Additionally, ADME predictions revealed that **5a** possesses favorable physicochemical and pharmacokinetic properties. Density Functional Theory (DFT) calculations provided insights into the electronic structure of **5a**, highlighting favorable HOMO–LUMO distribution and electrostatic potential surfaces that support its dual‐binding behavior.

## Graphical Abstract

**Figure:**
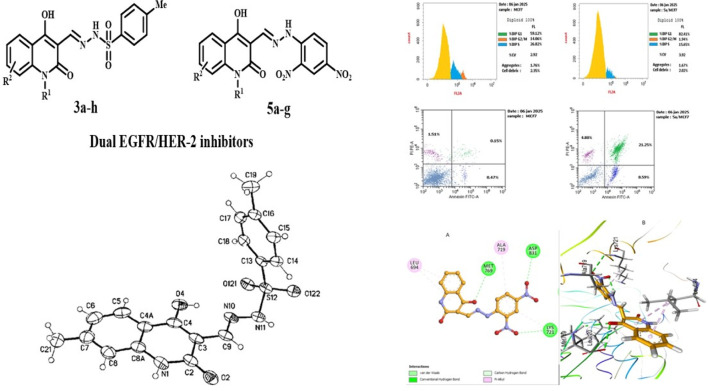
A series of new methylbenzene sulfonohydrazide and (2,4-dinitrophenyl)hydrazono based quinolin-2(1*H*)-one derivatives was designed and synthesised. The Structures of new compounds were validated by IR, NMR, elemental analysis and X-ray crystallography. The new compounds were evaluated as antiproliferative agent targeting EGFR and HER-2.

## Highlights

- A series of new Quinoline-based derivatives was designed and synthesised.
- The Structures of new compounds were validated by IR, NMR, elemental analysis and X-ray crystallography.
- The new compounds were evaluated as antiproliferative agent targeting EGFR, and HER-2.
- Antiproliferative activities were evaluated against four human cancer cell lines.

## 1 Introduction

The initial development and progression of cancer include several receptors and signaling pathways, demonstrating that multitargeting agents are more favorable than individual therapies (Al-Wahaibi et al., 2023a; Al-Wahaibi et al., 2024a). Multitargeting anticancer drugs aim to engage various biological receptors, anticipating synergistic effects and reduced toxicity compared to conventional therapy (Al-Wahaibi et al., 2023b). The two primary methods for identifying multitargeting drugs are screening techniques and knowledge-based strategies, wherein a rational design is established based on some pharmacophores that are retained while introducing another to produce hybrid compounds (Mahmo et al., 2024; Ravikumar et al., 2025).

HER-2 belongs to the EGFR family of tyrosine kinases, a broad category of proteins involved in various processes related to cell growth, proliferation, and differentiation (Hao et al., 2024). HER-2 plays an important role in a variety of cell signaling pathways. Gene amplification and transcriptional dysregulation cause HER-2 overexpression in breast cancer (BC), resulting in 25–50 copies of the gene. This causes a 40- to 100-fold increase in HER-2 expression, resulting in the development of up to 2 million HER-2 receptors on the cell surface (Gutierrez and Schiff, 2011). Additionally, HER-2-positive breast cancer exhibits a propensity for metastasis, particularly to the brain (Zimmer et al., 2022).

In addition to HER-2, there are three forms of EGFRs: EGFR, HER-3, and HER-4. Several investigations indicate that EGFR and HER-2 experience coamplification in numerous cancer types, including those of the breast, ovaries, prostate, colon, and other tissues (Alkahtani et al., 2020; Luhtala, 2019; Maennling et al., 2019). Overexpression of the HER-2 receptor in breast cancer is often associated with improper diagnosis and treatment resistance. Co-overexpression of EGFRs contributes to suboptimal diagnosis and treatment resistance in BC. According to the reports, simply suppressing HER-2 is insufficient for treating HER-2+ breast cancer. The complementary functions and interrelationships among HER-2 family members justify simultaneously targeting HER-2 and EGFR (Ghorab et al., 2018; Soliman et al., 2019).

**FIGURE 1:**
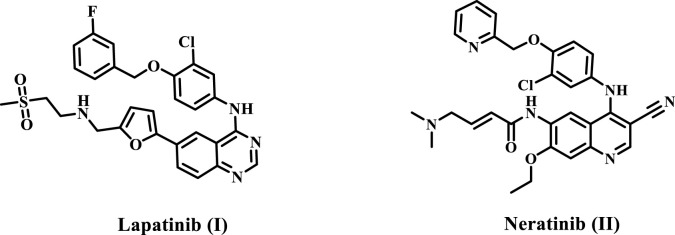
Structures of dual EGFR/HER-2 inhibitors lapatinib and neratinib.

Lapatinib (Compound **I**, Figure 1) is an FDA-approved dual inhibitor of HER-2 and EGFR for HER2-positive breast cancer, granted approval in 2007. However, multiple cases of lapatinib-resistant breast cancer have emerged recently (Pernas and Tolaney, 2019; Tsang et al., 2011). Neratinib (Compound **II**, Figure 1) is a multi-targeting inhibitor of the EGFR family, which received FDA approval in 2017 for HER-2-positive breast cancer (Abourehab et al., 2021; Wu et al., 2023). Phase III study data indicate that neratinib presents multiple adverse effects, such as diarrhea, gastrointestinal tract toxicity, and other side effects, mostly linked to heightened cytochrome P4503A4 activity during metabolism (Harding et al., 2023; Piha-Paul et al., 2023).

**FIGURE 2:**
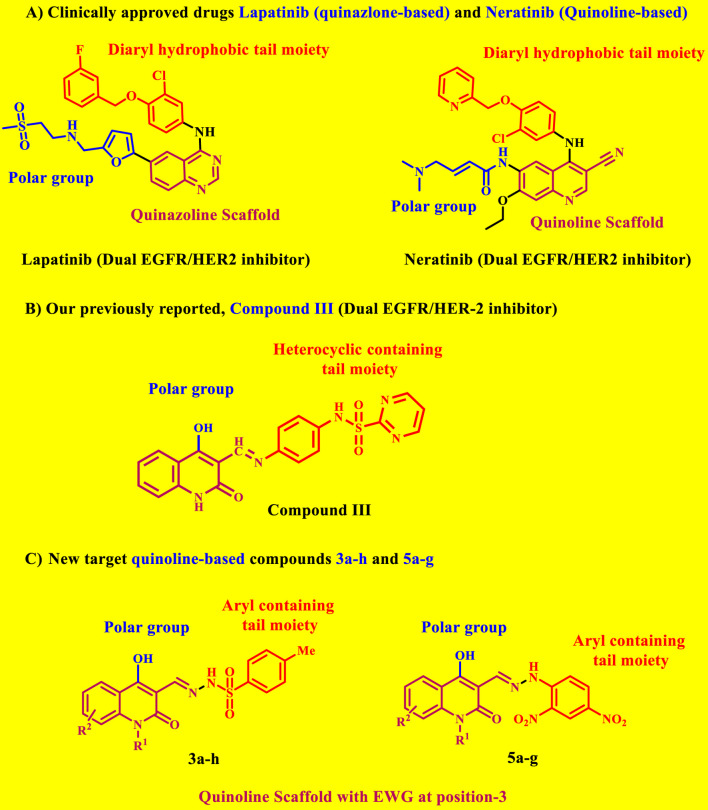
Rational design of **(A)** Lapatinib and Neratinib; **(B)** previously reported compound **III**; **(C)** New target compounds **3a-h** and **5a-g**.

Our recent work (Voigtlaender et al., 2018) describes the design, synthesis, and antiproliferative efficacy of novel quinoline-based compounds functioning as dual EGFR/HER-2 inhibitors. Compound **III** (Figure 2B) was recognized as the most effective dual inhibitor of EGFR and HER-2, with IC_50_ values of 71 and 31 nM, respectively. Compound **III** had more potency than erlotinib as an EGFR inhibitor, displaying equivalent efficacy to Lapatinib as a HER-2 inhibitor. Compound **III** demonstrated significant antitumor activity against a panel of cancer cell lines, with a GI_50_ value of 25 nM, compared to erlotinib’s GI_50_ of 33 nM. Compound **III** was evaluated on four cancer cell lines, with the breast (MCF-7) cancer cell line being the most sensitive, it demonstrated an IC_50_ value of 23 nM against the MCF-7 cell line, 1.8 times more potent than erlotinib (IC_50_ = 40 nM).

### 1.1 Rational design

Lapatinib **I** and Neratinib **II** (Figure 1) are strong inhibitors of both EGFR and HER-2. They obtained FDA approval as a monotherapy or in combination with other chemotherapeutics for the treatment of HER-2-positive metastatic breast cancer (Deeks, 2017; Voigtlaender et al., 2018). According to reports, lapatinib must be dissolved as a tosylate salt due to its limited water solubility. Consequently, clinical applications use lapatinib ditosylate. Simultaneously, the treatment of breast cancer has revealed the negative effects of lapatinib ditosylate, or Neratinib, which include gastrointestinal disorders, hepatic impairment, and arrhythmia (Deeks, 2017). In response to these limitations, researchers developed innovative EGFR/HER-2 dual inhibitors that fight cancers, have fewer side effects, and have enhanced water solubility.

Weissner et al. reported that the nitrogen atom at position three of the quinazoline ring could be replaced by a C-X, where X represents an electron-drawing group (Wissner et al., 2000). Accordingly, this paper describes the design, synthesis, and biological evaluation of new EGFR and HER-2 **3a-h** and **5a-g** dual inhibitors (Figure 2C). We chose lapatinib (quinazoline-based drug) and neratinib (quinoline-based drug) as the lead molecules for these inhibitors. The proposal calls for a quinoline core scaffold with an Azomethine (Schiff base) group at position 3, and a hydrophobic tail containing a benzene ring (Figure 2C), which shows that the hydrophobic tail may contain a *p*-toluene sulphonyl moiety (**3a-h**) or a dinitrobenzene ring (**5a-g**).

## 2 Results and discussion

### 2.1 Chemistry

**SCHEME 1:**
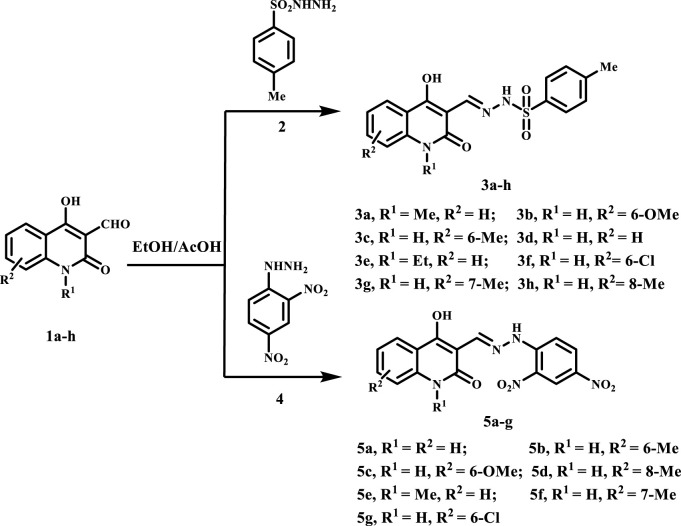
Synthesis of hydrazone derivatives **3a-h** and **5a-g.**

The present study focuses on the synthesis of a new series of (*E*)-*N*'-((4-hydroxy-7-2-oxo-1,2-dihydroquinolin-3-yl)methylene)benzenesulfonohydrazides **3a-h** and (*E*)-3-((2-(2,4-dinitrophenyl)hydrazono)methyl)-4-hydroxyquinolin-2(1*H*)-ones **5a-g** which were obtained in 79%–92% and 76%–90% yields, respectively. The reaction occurs through a straightforward condensation between quinoline-3-carbaldehydes **1a-h** and 4-methylbenzene sulfonohydrazide **2**, along with (2,4-dinitrophenyl)hydrazine **4**, conducted in absolute ethanol and few drops of glacial acetic acid, as depicted in Scheme 1.

The structures of the new compounds were elucidated using various spectroscopic techniques, such as IR, NMR spectroscopy, elemental analysis, and mass spectrometry. At the same time, geometrical configuration is validated using X-ray crystallographic studies. Compound **3a**, as a representative example, exhibit a molecular formula C_18_H_17_N_3_O_4_S, with a molecular weight *m/z* = 371. This result were confirmed by elemental analysis and mass spectrometry. On the other hand, to complete the proof of our results, the ^1^H NMR spectrum of compound **3a** revealed a three broad singlet signals at a downfield shift of δ_H_ = 12.90, 11.71, and 8.36 ppm, attributed to the quinolone-OH proton (Al-Wahaibi et al., 2024b), the hydrazonyl-NH proton (Al-Wahaibi et al., 2023c), and CH = N-proton (Al-Wahaibi et al., 2024b), respectively. Additionally, the tosyl-ring protons exhibit two doublet signals attributed to the 1,4-disubstituted benzene ring system; one signal is observed at δ_H_ = 7.45–7.47 ppm (d, *J* = 8.0 Hz, 2H), while the other doublet is noted at δ_H_ = 7.73–7.75 ppm (d, *J* = 8.0 Hz, 2H). The four protons of the quinolone ring appear within the range of δ_H_ = 7.26–7.76 ppm. Also, two singlet signals exhibiting upfield shifts were found at δ_H_ = 2.36 and 3.52 ppm, which were assigned as the methyl group of the tosyl ring and the *N*-methyl groups, respectively.

The ^13^C NMR spectrum of **3a** exhibited two signals resonating upfield at δ_C_ = 21.05 and 28.96 ppm, attributed to the methyl groups of the *p*-tosyl moiety and the N-methyl, respectively. Additionally, two downfield shift values with δ_C_ = 164.47 and 160.60 ppm are designated as amide-CO and C-4 linked to the hydroxyl group. Additionally, the CH = N was detected resonating at δ_C_ = 149.69 ppm. The aromatic-CH resonated at δ_C_ = 122.15, 124.02, 127.18, and 130.10 ppm. The quaternary carbons resonate at 115.10, 133.16, 134.93, 140.06, and 144.27 ppm.

**FIGURE 3:**
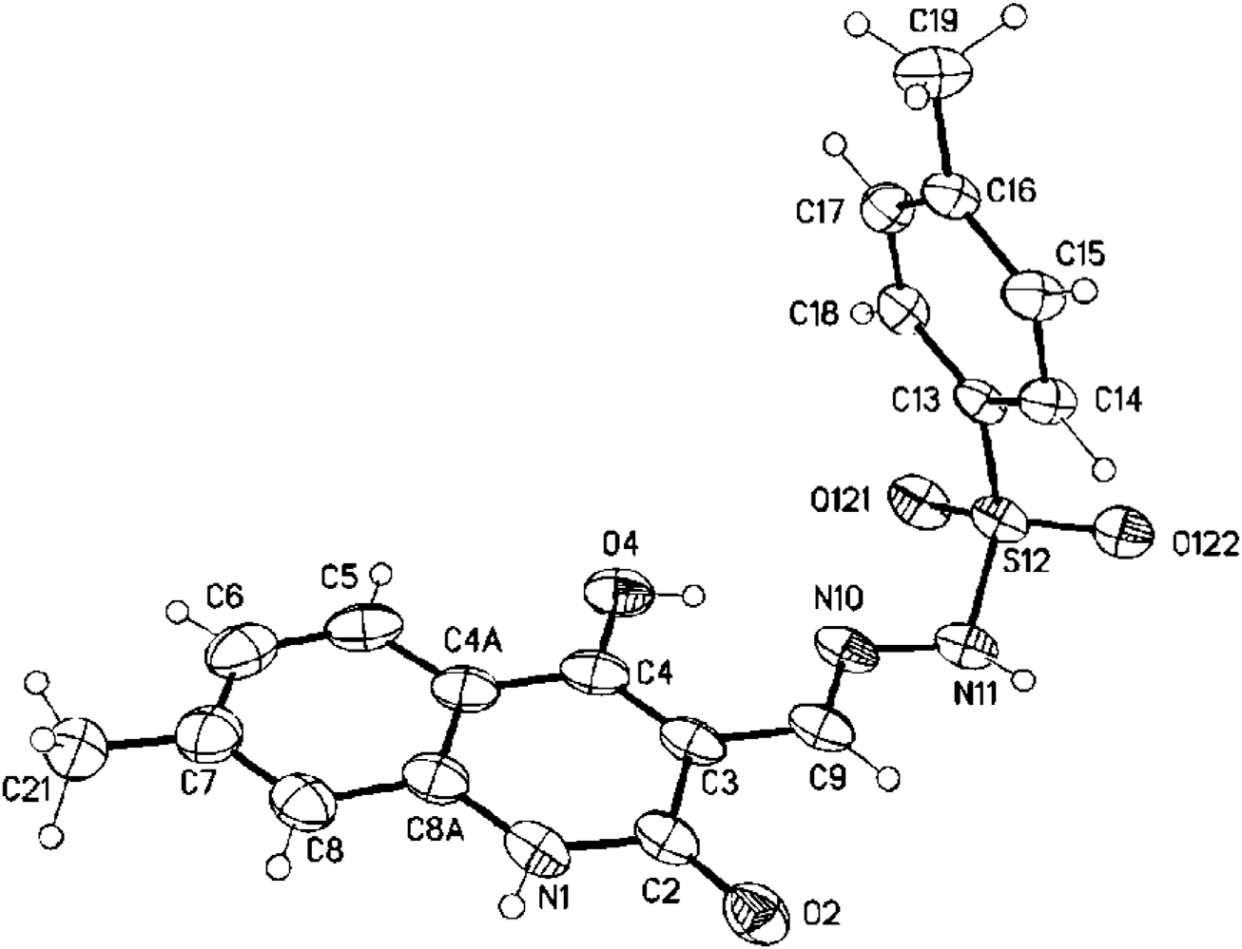
Molecular structure of (*E*)-*N*'-((4-hydroxy-7-methyl-2-oxo-1,2-dihydro-quinolin-3-yl)methylene)-4-methylbenzenesulfonohydrazide **3g** (approx. 30% displacement parameters are drawn at 30% probability level).

**FIGURE 4:**
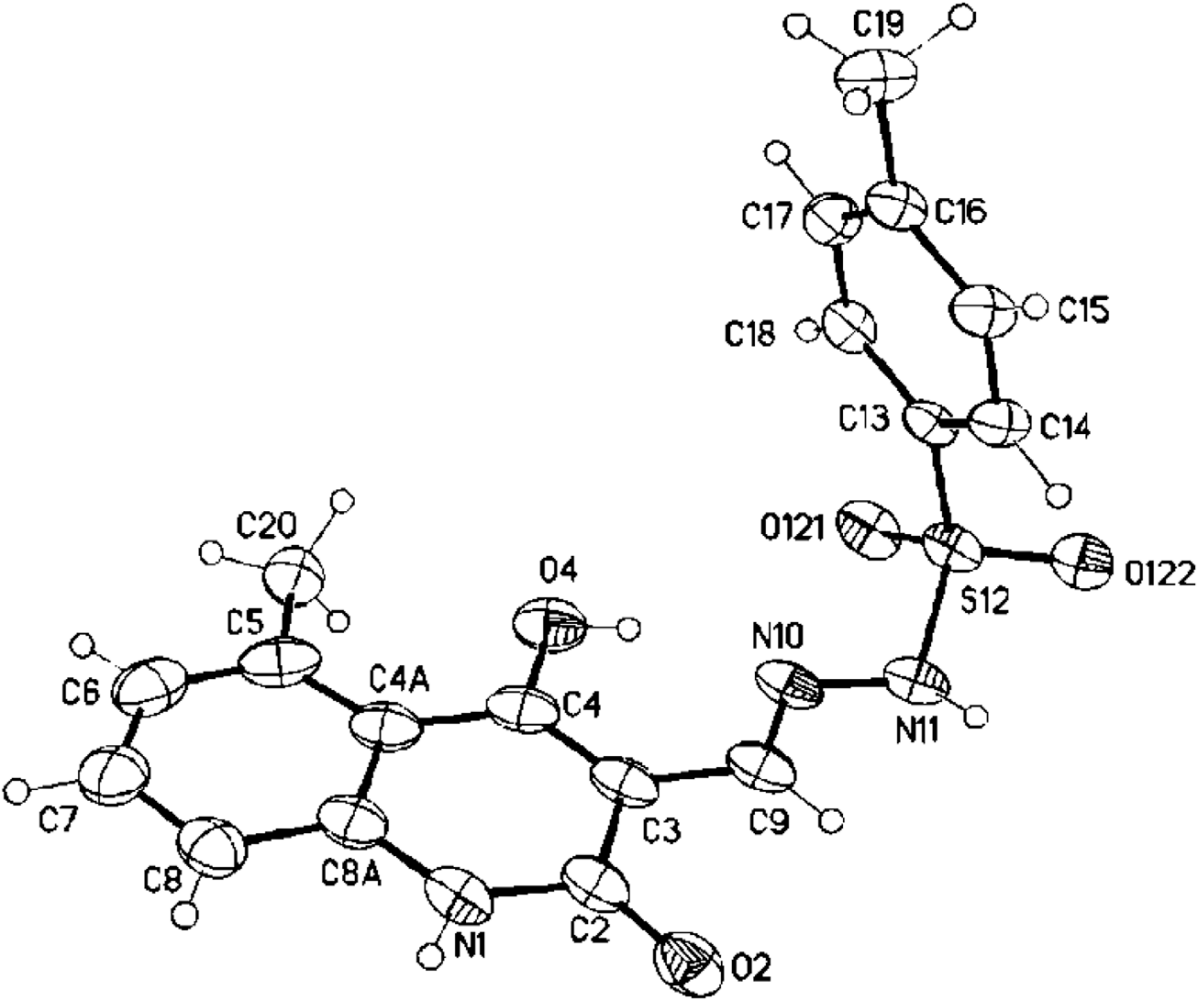
(*E*)-*N*'-((4-hydroxy-5-methyl-2-oxo-1,2-dihydroquinolin-3-yl)methylene)-4-methylbenzenesulfonohydrazide **3g`** (approx. 70%, displacement parameters are drawn at 30% probability level).

Additionally, the X-ray study dispelled any uncertainties regarding the validity of our findings. The geometric configuration of the produced compounds was validated using X-ray crystallographic analysis of compound **3g**, which displayed the predominant *E*-geometry as evidenced by the crystal molecular structure. The X-ray crystallographic analysis reveals that compound **3g** exists as two positional isomers: (*E*)-*N*'-((4-hydroxy-7-methyl-2-oxo-1,2-dihydroquinolin-3-yl)methylene)-4-methylbenzenesulfono-hydrazide (**3g**) and (*E*)-*N*'-((4-hydroxy-5-methyl-2-oxo-1,2-dihydroquinolin-3-yl)methylene)-4-methylbenzenesulfonohydrazide (**3g′**), as illustrated in Figures 3, 4, respectively.

**FIGURE 5:**
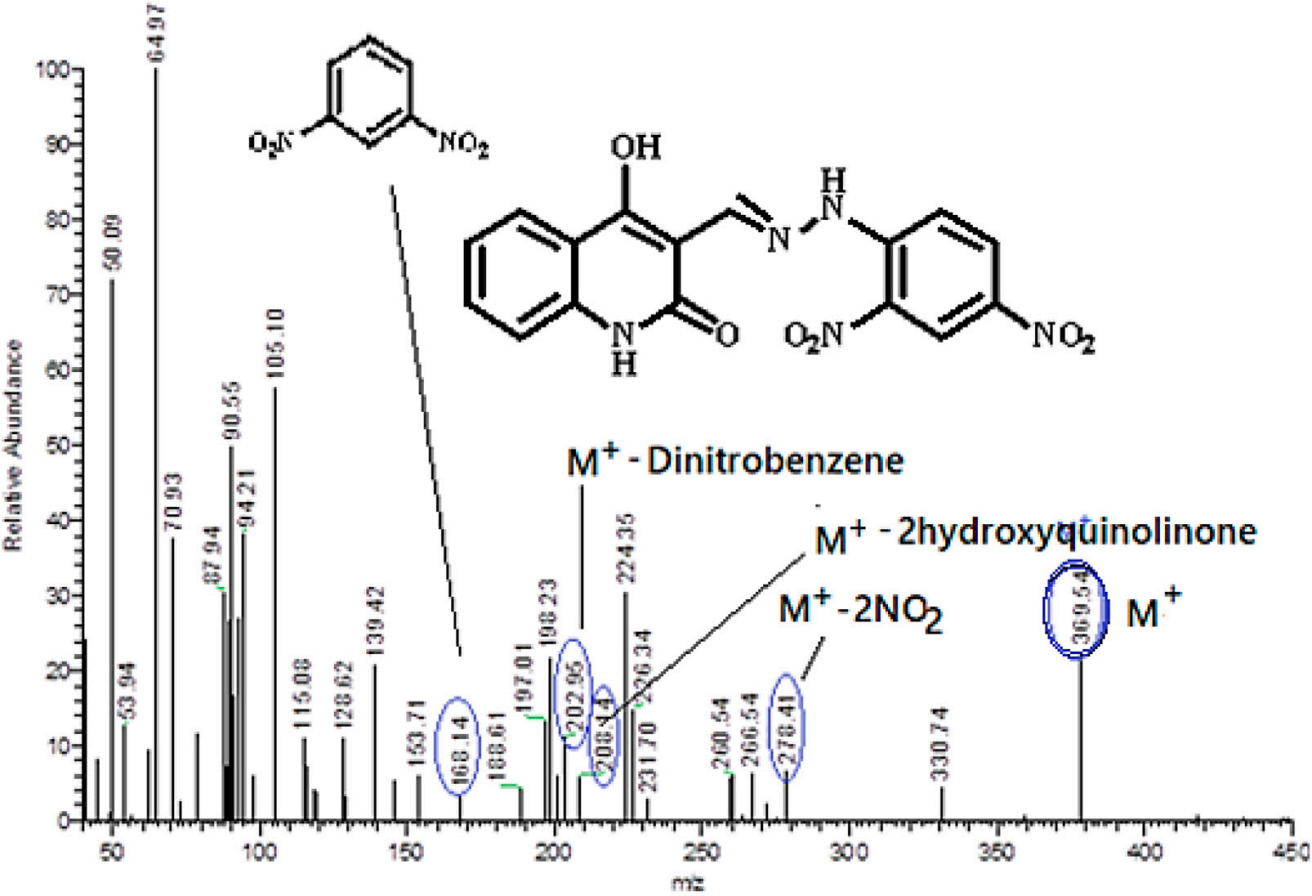
Mass spectrometry and fragmentation patterns for compound (*E*)-3-((2-(2,4-dinitrophenyl)hydrazono)methyl)-4-hydroxyquinolin-2(1*H*)-one (**5a**).

Conversely, the condensation reactions of quinoline-3-carbaldehydes **1a-h** with (2,4-dinitrophenyl)hydrazine (**4**) result in the synthesis of a novel series of (*E*)-3-((2-(2,4-dinitrophenyl)hydrazono)-methyl)-4-hydroxyquinolin-2(1*H*)-ones **5a-g**, which were obtained in high to exceptional yields. We choose compound **5a** as an example which was assigned as (*E*)-3-((2-(2,4-dinitro-phenyl)hydrazono)methyl)-4-hydroxyquinolin-2(1*H*)-one. The structure was assigned based on spectral data, elemental analysis as well as mass spectrometry. The elemental analysis shows that this compound has a molecular formula C_16_H_11_N_5_ and its molecular formula completed by its mass spectrometry which give m/z = 369 to give its actual molecular formula as C_16_H_11_N_5_O_6_. Also, the mass spectrometry fragmentation for the obtained product was studied under electron ionization. The following common features of the fragmentation patterns lend support to the assigned structures: Loss 2NO_2_ groups giving rise to ion m/z = 278 (M^+^ - 92), m/z = 208 (M^+^ - 4-hydroxy-2-quinolinone), m/z = 202 (M^+^ - dinitrobenzene), and m/z = 168 (dinitrobenzene) (Figure 5). Compound **5a** was further confirmed from the ^1^H NMR spectrum which clearly shows the presence of four broad singlet signals at δ_H_ = 8.35, 11.18, 11.33 and 12.77 ppm., with the ratio (1:2:1) which were assigned as CH = N, quinolinon-NH, hydrazono-NH and hydroxyl group, respectively. Other two doublet signals with the ratio (2:2) at δ_H_ = 7.18–7.19 and 7.89–7.91 ppm, which were assigned as quinolinon-2H and dinitrobenzene-2H, respectively. In addition to other protons which appeared as multiplet at δ_H_ = 7.20–7.77 (m, 3H). Furthermore, the ^13^C NMR spectrum for compound **5a** exhibit common signals at δ_C_ = 101.10, 140.06, 144.28, 149.69, 161.10, and 166.43 ppm., which were assigned as (C-3), (Ar-C), (CH = N), (C-4) and quinolinone-C2, respectively.

### 2.2 Biology

#### 2.2.1 Cell viability assay

The MCF-10A normal cell line from the human mammary gland was used to study the impact of new targets **3a-h** and **5a-g** on cellular survival. The MTT assay was employed to determine the viability of **3a-h** and **5a-g** cells after 4 days of incubation with MCF-10A cells (El-Sherief et al., 2019; Ramadan et al., 2020). Table 1 shows that none of the tested compounds caused cell death, with over 86% of cells surviving at a 50 µM concentration.

**TABLE 1:** Cell Viability and IC_50_ values of compounds **3a-h** and **5a-g**.

Comp.	Cell viability %	Antiproliferative activity IC_50_ ± SEM (nM)				
		A-549	MCF-7	Panc-1	HT-29	Average (GI_50_)
**3a**	90	92 ± 9	89 ± 8	94 ± 9	94 ± 9	92
**3b**	92	56 ± 5	52 ± 5	56 ± 5	58 ± 5	56
**3c**	86	50 ± 5	48 ± 4	53 ± 5	52 ± 4	51
**3d**	91	40 ± 3	37 ± 3	43 ± 4	42 ± 4	41
**3e**	89	62 ± 6	61 ± 6	64 ± 6	64 ± 6	63
**3f**	90	46 ± 4	43 ± 4	46 ± 4	48 ± 4	46
**3g**	87	94 ± 9	90 ± 9	94 ± 9	96 ± 9	94
**3h**	90	>100	>100	>100	>100	>100
**5a**	88	36 ± 3	34 ± 3	38 ± 3	38 ± 3	37
**5b**	90	52 ± 5	50 ± 5	54 ± 5	54 ± 5	53
**5c**	89	65 ± 6	62 ± 6	69 ± 6	68 ± 6	66
**5d**	92	71 ± 7	67 ± 6	74 ± 7	74 ± 7	72
**5e**	87	76 ± 7	74 ± 7	76 ± 7	78 ± 7	76
**5f**	90	85 ± 8	83 ± 8	86 ± 8	86 ± 8	85
**5g**	91	81 ± 8	78 ± 7	82 ± 8	82 ± 8	81
**Erlotinib**	ND	30 ± 3	40 ± 3	30 ± 3	30 ± 3	33

#### 2.2.2 Antiproliferative assay

The MTT assay (Al-Wahaibi et al., 2022; Mahmoud et al., 2023) was employed to assess the antiproliferative effects of compounds **3a-h** and **5a-g** on four human cancer cell lines: colon (HT-29) cancer, pancreatic (Panc-1) cancer, lung (A-549) cancer, and breast (MCF-7) cancer cell lines. Erlotinib was applied as a reference. Table 1 presents the median inhibitory concentration (IC_50_) and average IC_50_ (GI_50_) values for each compound evaluated on the four cancer cell lines.

Compounds **3a-h** and **5a-g** had significant antiproliferative activity, with GI_50_ values ranging from 37 to 100 nM, compared to the reference erlotinib (GI_50_ = 33 nM). In all cases, the compounds under investigation have lower potency than erlotinib. Moreover, all examined compounds have a higher affinity for breast cancer (MCF-7) cell line than the other cell lines studied. Compounds **3c**, **3d**, **3f**, **5a,** and **5b** had the highest antiproliferative activity, with GI_50_ values between 37 and 53 nM. Derivatives **3d** and **5a** are more effective than erlotinib against the MCF-7 breast cancer cell line. Their IC_50_ values were 37 nM and 34 nM, respectively, while erlotinib exhibited an IC_50_ value of 40 nM.

Compound **5a** (R^1^ = R^2^ = H, Scaffold B) surpassed all other tested compounds. It exhibited a GI_50_ of 37 nM, rendering it 1.2 times less effective than erlotinib (GI_50_ = 33 nM) against the four cancer cell lines tested. Compound **5a** exhibited substantial antiproliferative activity against the MCF-7 breast cancer cell line, with an IC_50_ value of 34 nM, 1.2 times more effective than erlotinib’s IC_50_ value of 40 nM. Furthermore, compound **5a** has marginally reduced potency compared to erlotinib against the other three cell lines, as seen in Table 1.

The substitution pattern of the quinoline moiety markedly influences the antiproliferative efficacy of compounds **3a-h** and **5a-g**. For instance, compound **5e** (R^1^ = Me, R^2^ = H, Scaffold B), an *N*-methyl derivative, had lower efficacy as an antiproliferative agent than **5a** (R^1^ = R^2^ = H, Scaffold B). Compound **5e** exhibited a GI_50_ of 76 nM, which is twice as low as that of **5a**, indicating that the presence of a free nitrogen atom at position 1 (N-1) of the quinoline moiety is more supportive of antiproliferative activity than the *N*-methyl group.

A different example is the 6-methyl derivative, compound **5b** (R^1^ = H, R^2^ = 6-Me, Scaffold B), the 6-methoxy derivative, **5c** (R^1^ = H, R^2^ = 6-OMe, Scaffold B), and the 6-chloro derivative, **5g** (R^1^ = H, R^2^ = 6-Cl, Scaffold B), all of which were found to be less efficient than the unsubstituted derivative, **5a** (R^1^ = R^2^ = H, Scaffold B). Compounds **5b**, **5c**, and **5g** exhibit IC_50_ values of 53, 66, and 81 nM, respectively, signifying their reduced potency compared to **5a** (GI_50_ = 37 nM). These findings indicate that derivatives possessing an unsubstituted quinoline moiety at the six position exhibit greater efficiency than those substituted with either electron-donating methyl and methoxy groups or an electron-withdrawing chlorine atom. Also, the 8-methyl derivative, compound **5d** (R^1^ = H, R^2^ = 8-Me, Scaffold B), and the 7-methyl derivative, **5f** (R^1^ = H, R^2^ = 7-Me, Scaffold B), were both shown to be less efficient than the unsubstituted derivative, **5a**. To establish an optimal structural-activity relationship (SAR), derivatives of the quinoline moiety’s phenyl ring must be modified with an electron-withdrawing group, such as a halogen atom or nitro group, at various positions of the quinoline structure. These specific modifications are now under investigation in our laboratory.

Compound **3d** (R^1^ = R^2^ = H, Scaffold A) exhibited the second greatest activity, with a GI_50_ value of 41 nM, 1.3-fold less active than erlotinib (GI_50_ = 33 nM). However, **3d** had superior activity to erlotinib against the MCF-7 breast cancer cell line, as indicated in Table 1. Replacing the C6-H of the quinoline moiety in compound **3d** with C6-methoxy in compound **3b** (R^1^ = H, R^2^ = 6-OMe, Scaffold A), a methyl group in compound **3c** (R^1^ = H, R^2^ = 6-Me, Scaffold A), or a chlorine atom in compound **3f** (R^1^ = H, R^2^ = 6-Cl, Scaffold A) led to a marked reduction in antiproliferative activity. The GI_50_ values for **3b**, **3c**, and **3f** were 56, 51, and 46 nM, respectively, demonstrating a potency reduction of 1.4-, 1.3-, and 2.5-fold compared to **3d** (GI_50_ = 41 nM). This corroborates the idea that the quinoline molecule’s unsubstituted phenyl ring exhibited greater activity tolerance.

It is noteworthy that the *N*-methyl derivative, **3a** (R^1^ = Me, R^2^ = H, Scaffold A), the C7-Me derivative, **3g** (R^1^ = H, R^2^ = 7-Me, Scaffold A), and the C8-Me derivative, **3h** (R^1^ = H, R^2^ = 8-Me, Scaffold A), demonstrate the lowest antiproliferative effect against all tested cell lines. The GI_50_ values for **3a**, **3g**, and **3h** were 92, 94, and >100 nM, respectively, indicating a potency decrease of at least 2.3-fold relative to **3d** (GI_50_ = 41 nM).

#### 2.2.3 EGFR inhibitory assay

The most effective antiproliferative derivatives, **3c**, **3d**, **3f**, **5a,** and **5b,** were evaluated for their ability to inhibit EGFR using the EGFR-TK test (Abou‐Zied et al., 2023). The results are presented in Table 2. Erlotinib operated as the reference compound.

**TABLE 2:** IC_50_ values of compounds **3c**, **3d**, **3f**, **5a** and **5b** against EGFR and HER-2.

Compound	EGFR inhibition IC_50_ ± SEM (nM)	HER-2 inhibition IC_50_ ± SEM (nM)
**3c**	99 ± 6	49 ± 3
**3d**	91 ± 6	38 ± 2
**3f**	95 ± 6	44 ± 3
**5a**	87 ± 5	33 ± 2
**5b**	106 ± 7	56 ± 4
**Erlotinib**	80 ± 5	--
**Lapatinib**	--	26 ± 1

The assay results align with those of the antiproliferative assay, indicating that compounds **5a** (R^1^ = R^2^ = H, Scaffold B) and **3d** (R^1^ = *R* ^2^ = H, Scaffold A), the most potent antiproliferative agents, were the most efficient derivatives of EGFR inhibitors, exhibiting IC_50_ values of 87 ± 5 and 91 ± 6 nM, respectively, in comparison to erlotinib’s IC_50_ value of 80 ± 5 nM. Results revealed that compounds **3d** and **5a** were less potent as EGFR inhibitors than the reference drug erlotinib. Compounds **3c** and **3f** inhibited EGFR significantly, with IC_50_ values of 95 and 99 nM, respectively, and were 1.2-fold less effective than the reference erlotinib. Ultimately, compound **5b** exhibited the lowest potency as an EGFR inhibitor, with an IC_50_ value of 106 ± 7 nM.

These data suggest that compounds **3d** and **5a** are effective antiproliferative candidates that could operate as EGFR inhibitors.

#### 2.2.4 HER-2 inhibitory assay

Compounds **3c**, **3d**, **3f**, **5a** and **5b** were evaluated for their capacity to inhibit HER-2 by a kinase assay (Al-Wahaibi et al., 2025). The findings are displayed in Table 2. Lapatinib functioned as the reference drug. The findings indicated that the investigated compounds markedly suppressed HER-2, exhibiting IC_50_ values between 33 and 56 nM, in contrast to lapatinib’s IC_50_ of 26 nM. The evaluated compounds exhibited lower potency in each case than the lapatinib reference medication. Compound **5a** was the most efficient HER-2 inhibitor, with an IC_50_ value of 33 nM, 1.3 times less potent than lapatinib. The data indicate that compound **5a** is a promising antiproliferative candidate with dual inhibitory activity against EGFR and HER-2, necessitating structural modifications for lead optimization.

#### 2.2.5 Apoptotic markers assay

Deficiencies in apoptosis within cancer cells significantly hinder the therapeutic effectiveness of anticancer drugs; therefore, developing of new therapies that target programmed cell death has become an essential objective for clinical use (Hisham et al., 2019; Bräse). Consequently, to reveal the pro-apoptotic potential of our target compounds, compounds **3d** and **5a** were evaluated for their capacity to initiate the apoptosis cascade.

##### 2.2.5.1 Activation of caspases 3, 8, and 9

Activating caspases is crucial in initiating and concluding the apoptotic process (Wu et al., 2025). Caspase-3 is a crucial enzyme that cleaves several proteins within cells, resulting in apoptotic cell death (Kouwenhoven et al., 2025). The impact of compounds **3d** and **5a** on caspase-3 was assessed and matched with Staurosporine as a reference medication (Table 3.

**TABLE 3:** Apoptotic markers assays of compounds **3d** and **5a**.

Compd. No.	Caspase-3		Caspase-8		Caspase-9		Cytochrome C	
	Conc (pg/mL)	Fold change	Conc (ng/mL)	Fold change	Conc (ng/mL)	Fold change	Conc (ng/mL)	Fold change
**3d**	515 ± 5	8.0	2.10 ± 0.20	23	21 ± 3	21	0.65	13
**5a**	570 ± 5	9.0	2.65 ± 0.25	29	24 ± 1	24	0.85	17
Staurosporine	465 ± 4	7.0	1.85 ± 0.15	21	20 ± 1	20	0.50	10
Control	65	1.0	0.09	1	1	1	0.05	1

The findings indicated that **5a** was the most potent derivative, exhibiting a significant overexpression of caspase-3 protein levels (570 ± 5 pg/mL) compared to the reference staurosporine (465 ± 4 pg/mL). Compound **3d** exhibited a 9-fold rise in active caspase-3 levels compared to control cells and induced caspase-3 levels surpassing those of staurosporine, the reference medication. Compound **3d** demonstrated an 8-fold increase in active caspase-3 levels (515 ± 5 pg/mL) compared to the control untreated cells, as shown in Table 3.

To elucidate the apoptotic mechanism of compounds **3d** and **5a**, whether *via* the intrinsic or extrinsic pathway, their impact on caspase-8 and caspase-9 was evaluated. The results indicated that compound **5a** elevates the levels of caspase-8 and caspase-9 by 29 and 18-fold, respectively, while compound **3d** increases the levels of caspase-8 and caspase-9 by 23 and 15-fold, respectively, in comparison to the control cells. This suggests activation of both intrinsic and extrinsic pathways, with a more pronounced effect on the extrinsic pathway, as evidenced by the elevated levels of caspase-8 (Table 3).

##### 2.2.5.2 Cytochrome C assay

The level of Cytochrome C within the cell is crucial for activating caspases and initiating the intrinsic apoptosis pathway (Jan et al., 2025).

Compounds **3d** and **5a** were assessed for their activity against cytochrome C in the MCF-7 human breast cancer cell line, with results in Table 3. Compounds **3d** and **5a** induce a 13-fold and 17-fold increase in cytochrome C levels in MCF-7 human breast cancer cells compared to the control. The results above show that apoptosis may be linked to the overexpression of cytochrome C and the activation of intrinsic and extrinsic apoptotic pathways initiated by the compounds studied.

##### 2.2.5.3 Bax and Bcl-2 levels assay

Compounds **3d** and **5a** were further investigated for their impact on Bax and Bcl-2 levels in the MCF-7 human breast cancer cell line, using staurosporine as a reference, as detailed in Table 4.

**TABLE 4:** Bax and Bcl-2 levels for **3d**, **5a**, and Staurosporine on human breast (MCF-7) cancer cell line.

Compd. No.	Bax		Bcl-2	
	Conc (pg/mL)	Fold change	Conc (ng/mL)	Fold reduction
**3d**	310 ± 2	34	0.80	6
**5a**	320 ± 2	35	0.70	7
Staurosporine	290 ± 2	32	1.00	5
Control	9.00	1	5.00	1

The results indicated that **3d** and **5a** significantly elevated Bax levels compared to staurosporine. Compound **5a** demonstrated an induction of Bax at 320 pg/mL, comparable to staurosporine at 290 pg/mL, and exhibited a 35-fold increase relative to untreated MCF-7 cancer cells, followed by compound **3d** at 310 pg/mL with a 34-fold rise. Ultimately, compound **5a** induced a reduction in the anti-apoptotic Bcl-2 protein level (0.70 ng/mL), preceded by compound **3d** (0.80 ng/mL) in the MCF-7 cell line, in comparison to staurosporine (1.00 ng/mL).

#### 2.2.6 Flow cytometric cell cycle analysis

**FIGURE 6:**
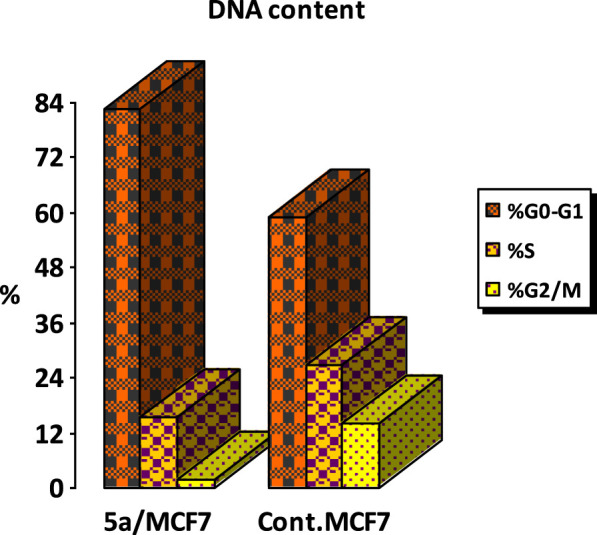
Cell cycle analysis of compound **5a** against the MCF-7 cancer cell line.

**FIGURE 7:**
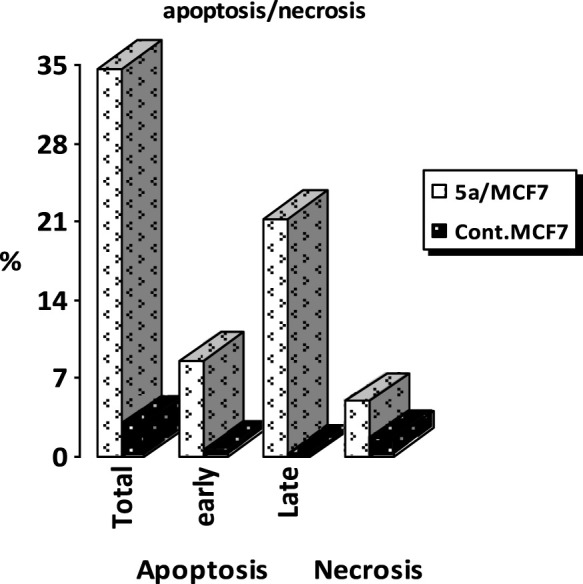
Percentage of apoptosis and necrosis of **5a** against the MCF-7 cancer cell line.

**FIGURE 8:**
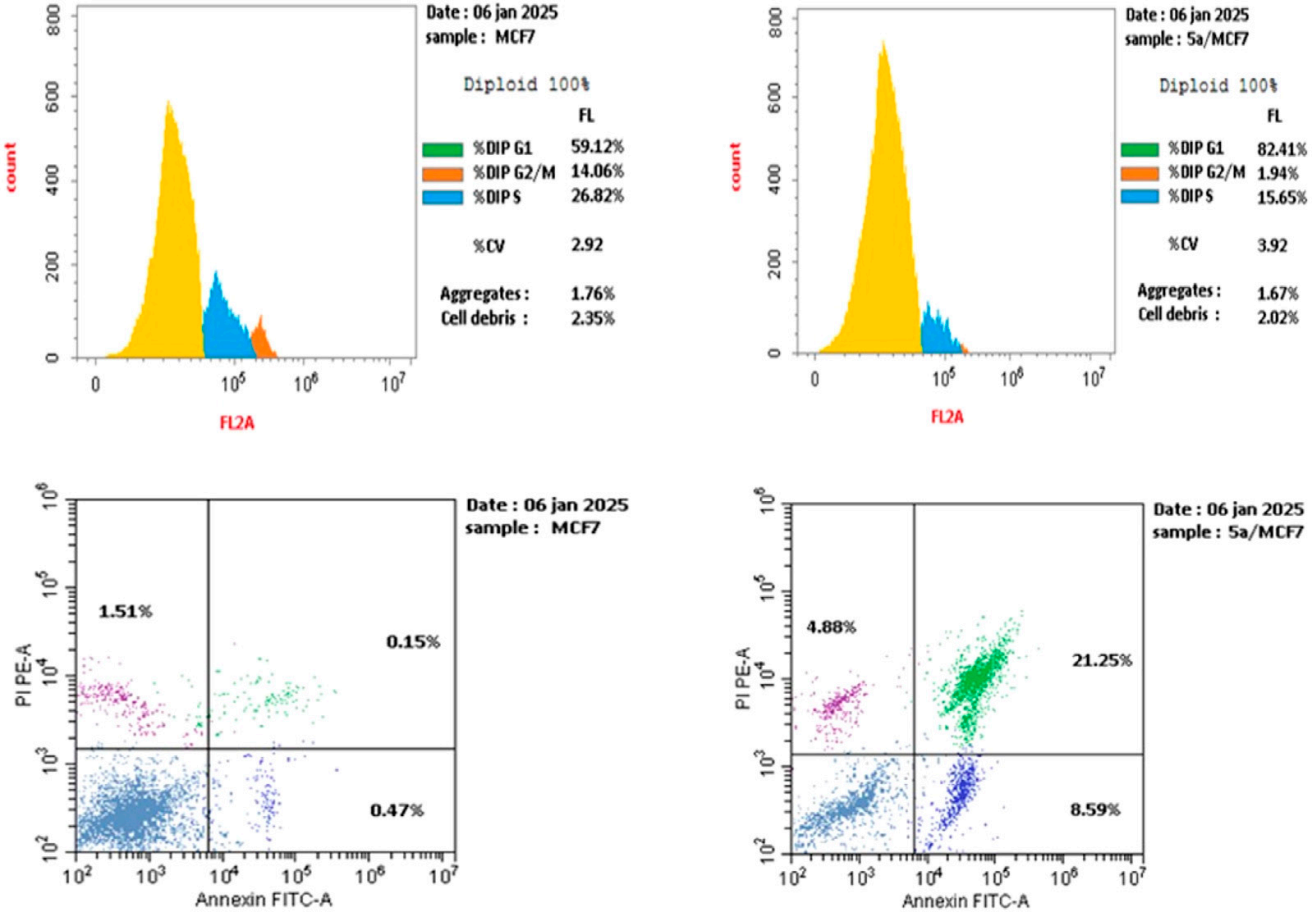
Cell cycle analysis and apoptosis induction of **5a** against the MCF-7 breast cancer cell line.

Cell cycle analysis has been done for the most potent compound **5a** against the MCF-7 human breast cancer cell line. The percentage of MCF-7 cells in the G0/G1 phase of the cell cycle in the control was 59.12%, which significantly increased to 81.42% following treatment with compound **5a**. In comparison, the percentage of cells in the S phase decreased slightly with compound **5a** (15.65%) compared to the control (26.82%) (Figure 6). The percentage of MCF-7 human breast cancer cells in the G2/M phase diminishes to 1.94% following treatment with compound **5a**. The data indicate that compound **5a** primarily induced cell cycle arrest in the G1 phase. Furthermore, it is evident that the examined compound is not cytotoxic; rather, it exhibits antiproliferative properties, inducing programmed cell death and cell cycle arrest (Figures 7, 8).

### 2.3 Docking study of EGFR and HER-2 enzymes

A comprehensive computational docking study was conducted to explore the binding interactions of compound **5a** with EGFR and HER-2 enzymes (Al-Wahaibi et al., 2025). The crystallographic structures of EGFR (PDB ID: 1M17) and HER-2 (PDB ID: 3PP0) were obtained and utilized as structural templates for the docking simulations, which were performed using the Discovery Studio software (Jejurikar and Rohane, 2021). Erlotinib and lapatinib were selected as reference ligands for EGFR and HER-2 (Al-Wahaibi et al., 2024b).

The docking simulations were performed using the OPLS-AA (Optimized Potentials for Liquid Simulations–All Atom) force field during the energy minimization process to ensure conformational stability of the ligand-protein complexes (R et al., 2022). This step is critical in enhancing the accuracy and reliability of computational predictions. Before the docking procedure, an extensive protein preparation protocol was followed, including appropriate protonation of the protein structures to improve their geometrical accuracy and optimize the docking results (Buccheri et al., 2025).

**FIGURE 9:**
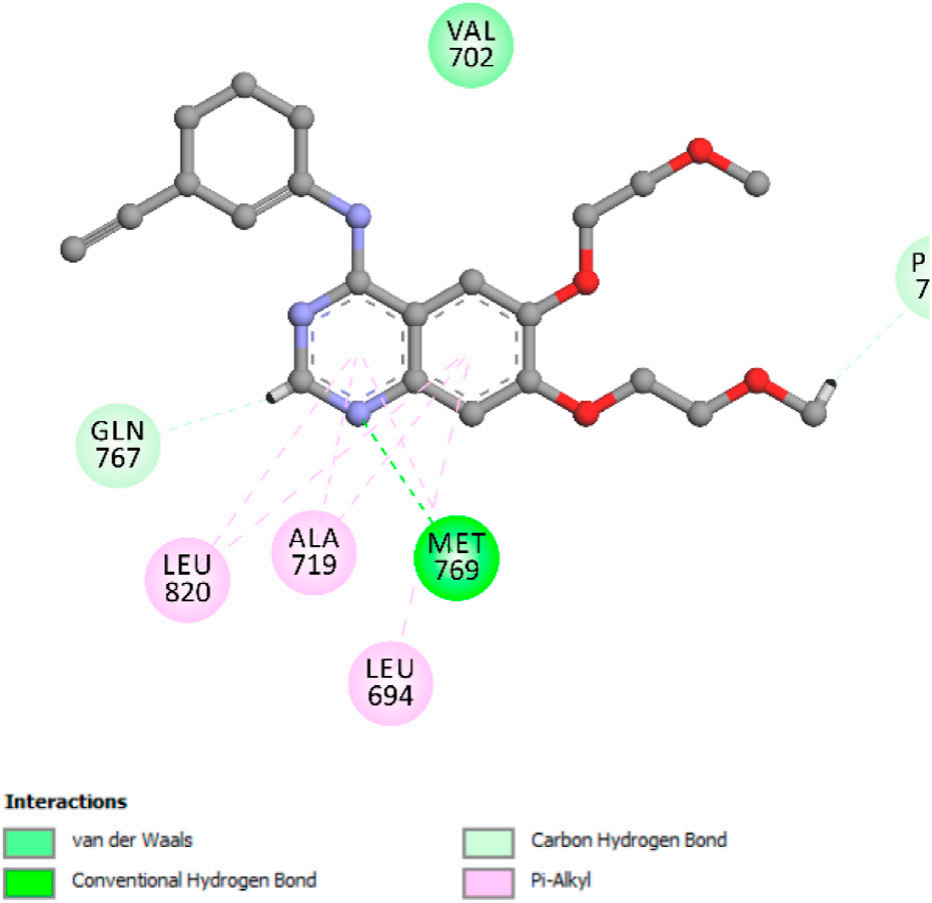
Two-dimensional docking model illustrating the binding orientation of erlotinib within the active site of EGFR.

To assess the validity of the docking protocol, the co-crystallized ligand erlotinib was re-docked into the EGFR binding site (Fayed et al., 2023). This validation step yielded a binding energy (S-score) of −8.05 kcal/mol, with a root mean square deviation (RMSD) value of 0.91 Å, indicating a reliable docking method. The re-docking simulation confirmed a key hydrogen bond interaction between the pyrimidine nitrogen of erlotinib and the Met769 residue in the EGFR active site, a crucial interaction that plays a significant role in stabilizing the ligand within the protein pocket (Figure 9).

The docking scores obtained for compound **5a** demonstrated a strong correlation with the *in vitro* inhibition activity of EGFR, thereby validating the predictive power of the docking methodology used in this study. This consistency between docking results and biological activity highlights the reliability of the docking protocol and its utility in identifying promising inhibitors for EGFR and HER-2 enzymes.

**FIGURE 10:**
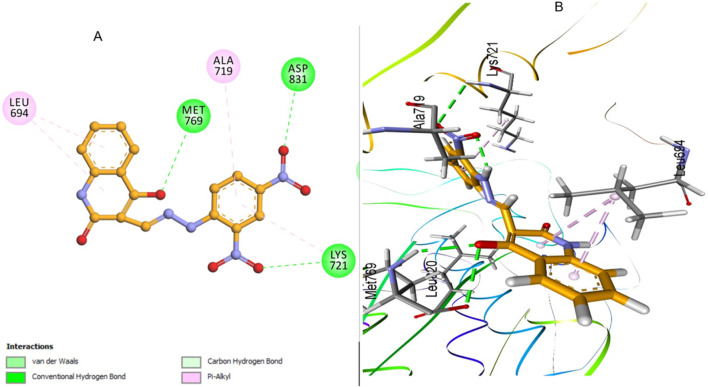
Two-dimensional **(A)** and three-dimensional **(B)** docking representations showing the binding interactions of compound 5a within the ATP-binding pocket of EGFR.

The docking analysis of compound **5a** within the ATP-binding site of EGFR revealed a highly favorable binding pose with a docking score of −7.33 kcal/mol, with a root mean square deviation (RMSD) value of 1.44 Å. The interaction profile shows that compound **5a** forms a pivotal hydrogen bond with the Met769 residue through its enol oxygen, anchoring the ligand within the active site (Figure 10). Additionally, two significant hydrogen bond interactions with the Asp831 and Lys721 residues further stabilize the compound. Notably, Pi-alkyl stacking interactions between the aromatic ring of quinolone and Leu694 strengthen the binding affinity by securing the ligand within the hydrophobic pocket **(** Figure 10 **)**.

Also, Pi-alkyl interaction with residue Ala719 further reinforces stability in the ATP-binding site. The 3D visualization depicts the precise orientation of 5a within the ATP-binding pocket, where the di-nitro phenyl core and hydrazone moiety are optimally aligned to maximize both hydrophobic and hydrogen-bonding interactions. The planar structure promotes effective alignment with the pocket, further enhancing binding efficacy (Figure 10).

To check the validity of the docking protocol for HER-2, the co-crystallized ligand was re-docked into the HER-2 binding site (Metibemu et al., 2021). This validation process yielded a binding energy (S-score) of −7.86 kcal/mol, with a root mean square deviation (RMSD) value of 1.24 Å, confirming the reliability of the docking procedure.

**FIGURE 11:**
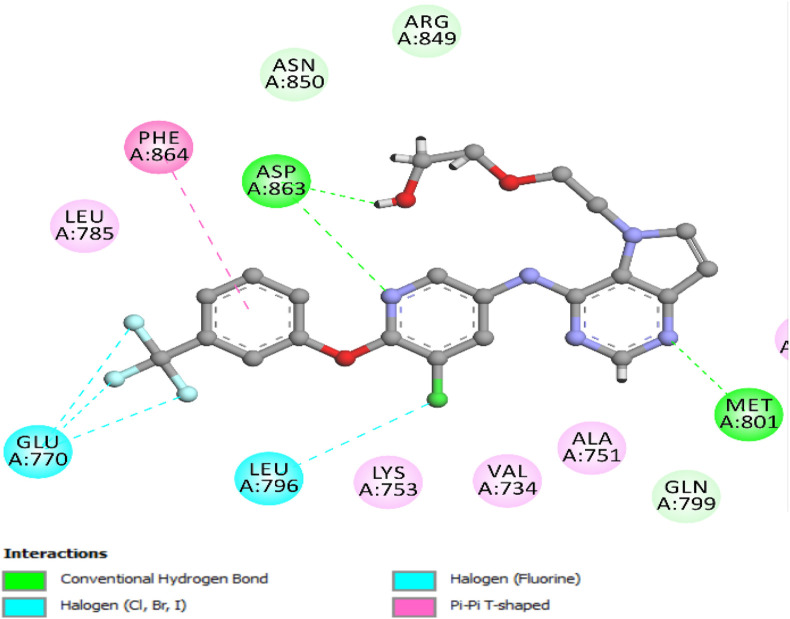
Two-dimensional docking model highlighting the binding interactions of the validated co-crystallized ligand within the HER-2 binding site.

The re-docking simulation revealed a key hydrogen bond interaction between the pyrimidine nitrogen of the ligand and the Met801 residue, which plays a crucial role in stabilizing the ligand within the HER-2 active site (Figure 11). The re-docking simulation revealed crucial interactions within the HER-2 binding pocket, including a key hydrogen bond between the nitrogen of the pyridine ring of the ligand and the Asp863 residue, contributing significantly to ligand stabilization. The docking interactions highlighted additional hydrogen bonds with Met801 and pi-pi T-shaped interactions with Phe864. Notably, halogen interactions with Glu770 and Leu796 further reinforced the binding within the active site (Figure 11).

**FIGURE 12:**
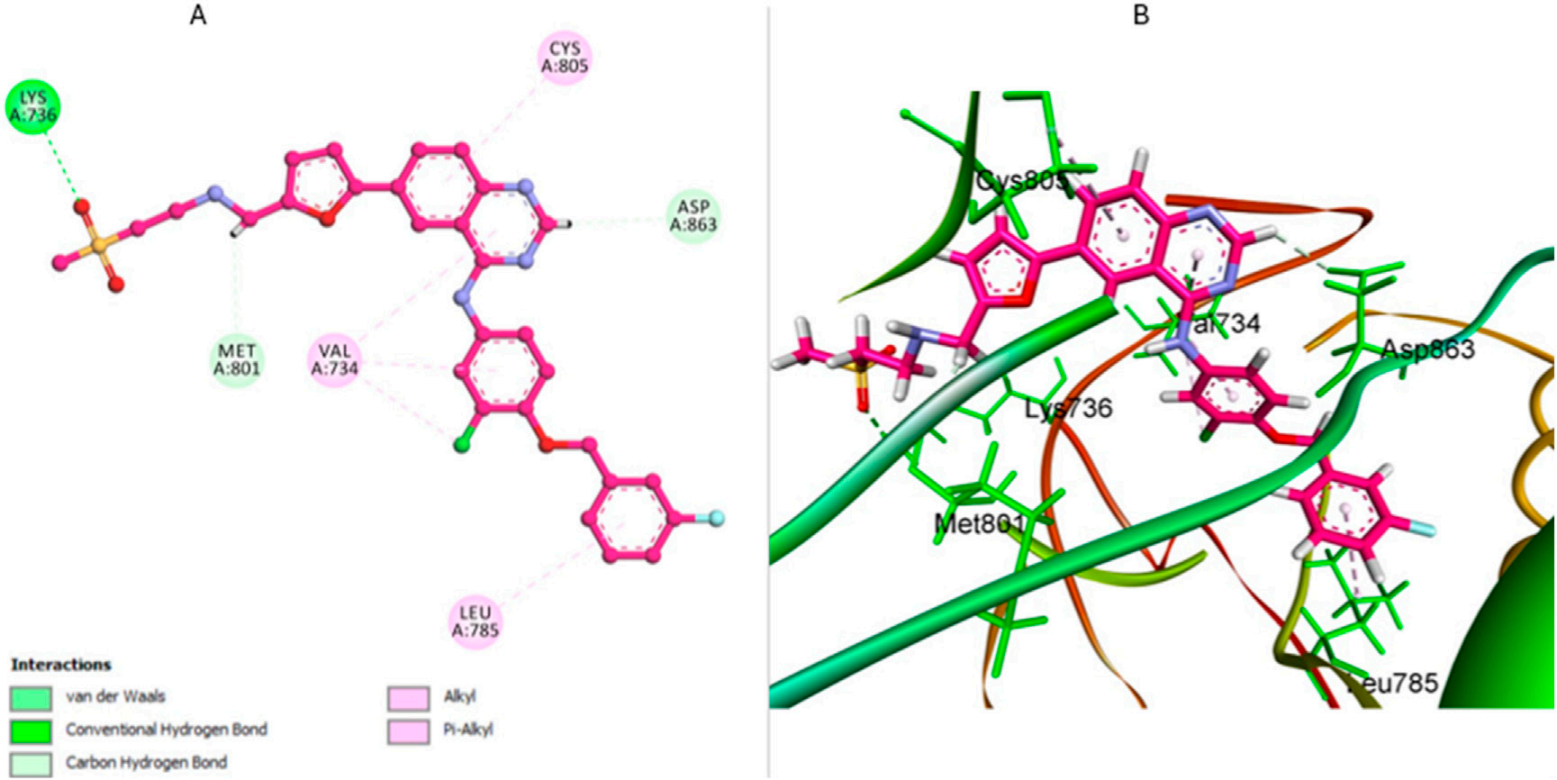
Docking representation of lapatinib within the HER-2 binding site; **(A)** 2D representation **(B)** 3D representation.

The docking analysis of lapatinib, a reference drug for HER-2 inhibition in our *in vitro* studies, provides valuable insights into its binding mode within the HER-2 active site. Lapatinib exhibits a favorable binding energy of −7.47 kcal/mol with an RMSD value of 1.45 Å, indicating a stable pose within the HER-2 binding pocket. The analysis reveals the formation of an essential hydrogen bond between the sulfone oxygen atoms of lapatinib and Lys753. Also, lapatinib can form other carbon-hydrogen bonds with Met801 residue and Asp863, significantly stabilizing the binding site’s ligand (Figure 12). Also, lapatinib forms extensive hydrophobic interactions with key residue Leu785, contributing to a stable hydrophobic core. Pi-alkyl interactions with residue Val734 further enhance the binding stability. These pi-alkyl interactions establish a robust network, ensuring a tight fit of lapatinib within the HER-2 active site and improving its inhibitory potential against HER-2 (Figure 12).

The docking analysis of compound **5a** against HER-2 revealed a binding energy of −7.24 kcal/mol, indicating a strong affinity for the HER-2 active site with an RMSD value of 1.63 Å, confirming a stable pose within the HER-2 binding site—compound **5a** forms several key interactions within the binding pocket, contributing to its stability and binding efficacy.

**FIGURE 13:**
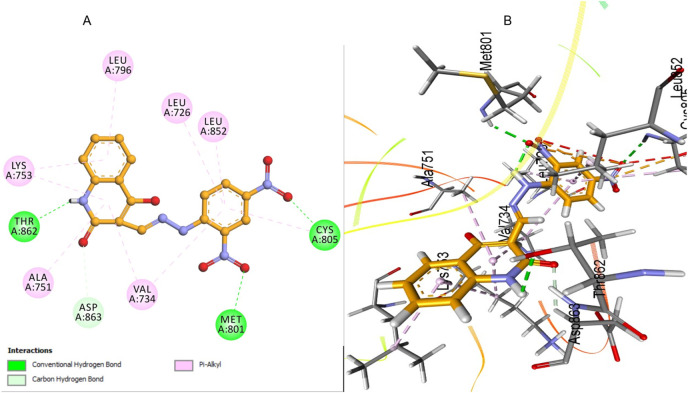
Docking representation of 5a within the HER-2 binding site; **(A)** 2D representation **(B)** 3D representation.

The most notable interactions include two important hydrogen bonds that enhance the stabilization within the HER-2 binding site. The first hydrogen bond is observed between the nitro group and Met801, reinforcing its position and stabilizing the binding conformation. The second hydrogen bond is established between the nitrogen of the quinolone and Thr862, securing the ligand within the pocket (Figure 13). In addition to these hydrogen bonds, 5a engaged in significant hydrophobic interactions between the di-nitro phenyl ring of **5a** with Leu726, Leu852, and Val734, contributing further to the overall conformational stability of the ligand.

The quinolone ring of **5a** also exhibited extensive hydrophobic contacts with residues such as Met801 and Lys753, which enhanced the tight fit of the compound within the HER-2 active site. The combination of these interactions ensured that **5a** maintained a secure and stable orientation within the binding site. When compared to lapatinib, the reference drug used in HER-2 inhibition studies, compound **5a** displayed a comparable binding profile with key differences in interaction patterns. Lapatinib demonstrated a binding energy, slightly higher than that of **5a**, indicating a comparable binding affinity. Both compounds exhibit crucial interactions with residues such as, Met801, and Val734, contributing to the overall binding stability.

Overall, the comparison between **5a** and lapatinib suggests that **5a** has a binding mode that is distinct yet similarly effective to that of the reference drug. Both compounds demonstrate strong hydrophobic interactions and hydrogen bonding, ensuring stable binding within the HER-2 binding pocket. These findings highlight the potential of **5a** as a promising HER-2 inhibitor with binding characteristics comparable to the clinically used reference drug, lapatinib, suggesting its possible utility in targeted HER-2 therapies.

The docking results for compound **5a** align well with the *in vitro* inhibition data, where **5a** demonstrated an IC_50_ of 33 ± 2 nM against HER-2, indicating potent inhibitory activity. In comparison, lapatinib, the reference drug for HER-2 inhibition, showed an IC_50_ of 26 ± 1 nM, slightly more potent than **5a**. When comparing compound 5a and erlotinib, the docking and *in vitro* results differ in their selectivity profiles. Erlotinib displayed an IC_50_ of 80 ± 5 nM against EGFR, highlighting its higher potency against EGFR than HER-2. On the other hand, **5a** demonstrated dual inhibition activity, with an IC_50_ of 87 ± 5 nM against EGFR and a more potent IC_50_ of 33 ± 2 nM against HER-2. This suggests that **5a** has a more balanced inhibitory effect on both targets than erlotinib and lapatinib. These findings highlight **5a** as a potential candidate for further development as a dual HER-2/EGFR inhibitor.

### 2.4 Molecular dynamics discussion of HER-2 for compounds **5a** and lapatinib

**FIGURE 14:**
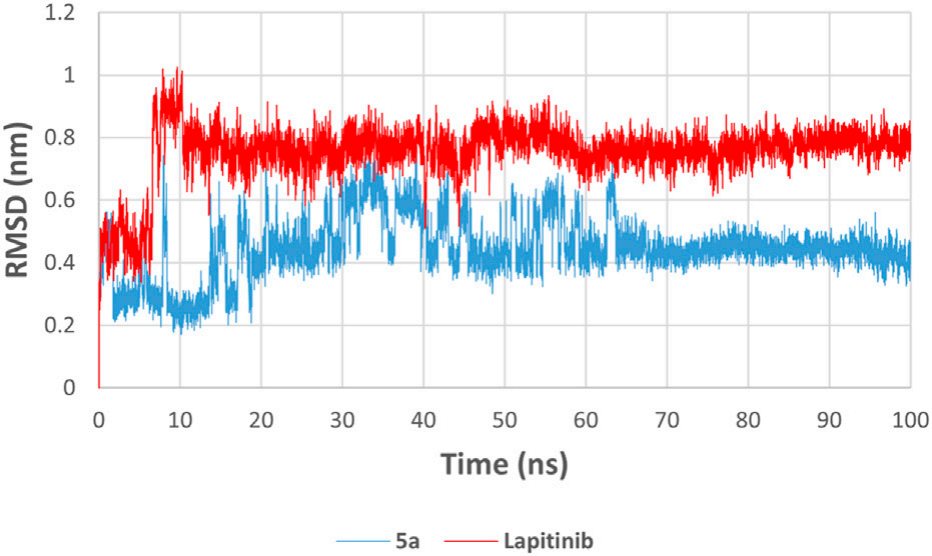
RMSD of HER-2-ligand complexes for **5a** (blue) and lapatinib (red) over a 100 ns molecular dynamics simulation.

Molecular Dynamics (MD) simulations serve as a critical complement to docking studies in drug discovery by providing detailed insights into the stability and dynamics of ligand-protein interactions (Bozorgpour et al., 2023; Kumar et al., 2024). While docking predicts a ligand’s initial binding pose and interactions within the active site, it offers a rigid snapshot of the complex (Stanzione et al., 2021). MD simulations, on the other hand, incorporate time-dependent molecular movements and environmental factors, allowing for a more realistic evaluation of the ligand-protein system (Adelusi et al., 2022). Through MD, key parameters such as stability (*via* RMSD), flexibility (*via* RMSF), compactness (*via* radius of gyration), and binding strength (*via* potential energy) can be assessed, offering a deeper understanding of the behavior of the ligand under physiological conditions (Tripathi et al., 2025). This dynamic perspective validates docking results and identifies critical interactions that stabilize the complex, helping to refine and prioritize drug candidates for experimental validation (Ghahremanian et al., 2022; Haider et al., 2020). By bridging the gap between computational predictions and experimental outcomes, MD simulations enhance the reliability and accuracy of virtual screening workflows (Kumar et al., 2025). The RMSD plot indicates the stability of the **5a** and lapatinib in the HER-2 binding pocket over a simulation period of 100 ns. Compound **5a** (blue) exhibits a significantly lower RMSD value, stabilizing at approximately 0.4–0.5 nm, compared to lapatinib (red), which fluctuates around 0.7–0.9 nm **(** Figure 14). The lower RMSD for 5a suggests a more stable binding conformation and less structural deviation during the simulation. Despite being a clinically established HER-2 inhibitor, Lapatinib shows higher fluctuations, indicating that compound **5a** may form a more rigid and stable complex with HER-2 under the tested conditions.

**FIGURE 15:**
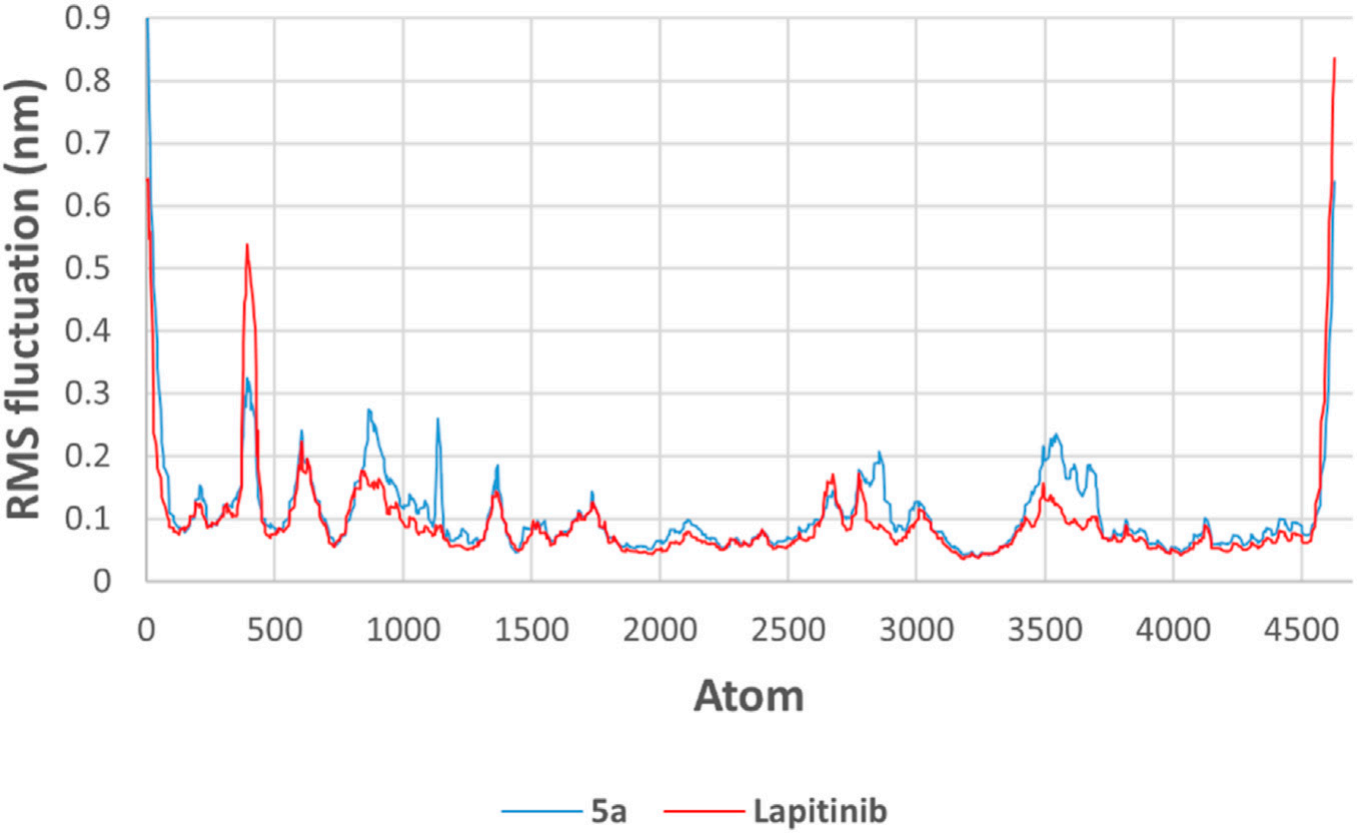
RMSF of HER-2 residues in complexes with **5a** (blue) and lapatinib (red).

The RMSF plot highlights residue-level flexibility within HER-2 upon binding with **5a** and lapatinib. Both compounds exhibit similar fluctuations for most regions, particularly in flexible loop regions and solvent-exposed residues. These findings suggest tighter binding and stabilization of the HER-2 active site of **5a (** Figure 15).

**FIGURE 16:**
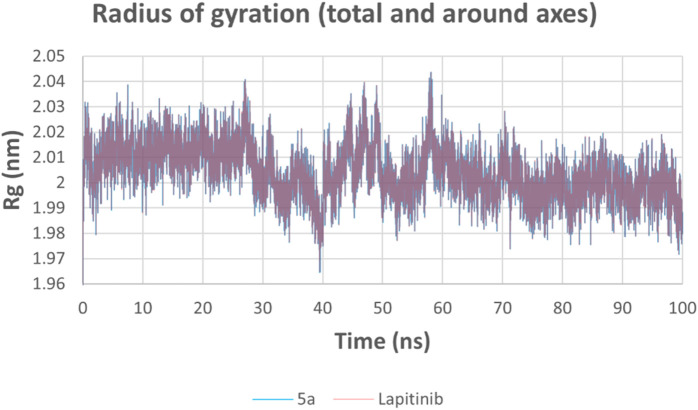
Radius of gyration (Rg) of HER-2-ligand complexes for **5a** (blue) and lapatinib (red).

The Rg plot measures the compactness of the HER-2-ligand complex during the simulation. Both complexes maintain consistent Rg values around 2.00–2.03 nm, indicating that the overall HER-2 protein structure remains stable throughout the simulation for both compounds. There is no significant difference in the fluctuation of Rg between **5a** and lapatinib, suggesting that **5a** stabilizes the HER-2 conformation comparably well (Figure 16).

**FIGURE 17:**
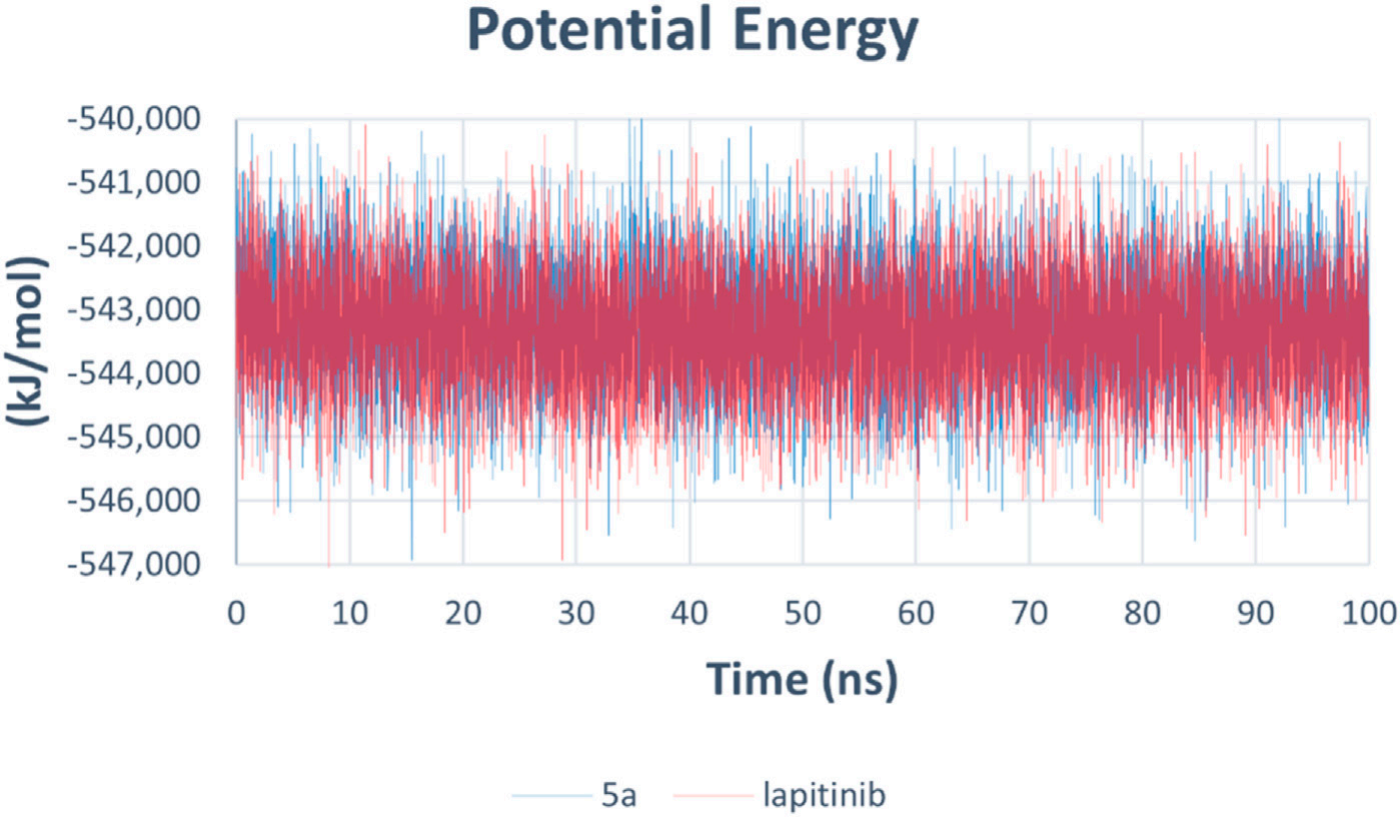
Potential energy of HER-2-ligand complexes for **5a** (blue) and lapatinib (red).

The potential energy analysis evaluates the stability of the HER-2-ligand systems. Both compounds exhibit consistent potential energy values throughout the 100 ns simulation, with no significant conformational disruptions observed (Figure 17).

The molecular dynamics simulations reveal compound **5a** and lapatinib demonstrate stable binding interactions with HER-2. **5a**’s lower RMSD and reduced flexibility indicate its potential as a promising HER-2 inhibitor. These findings highlight the need for further experimental validation of **5a**’s inhibitory efficacy against HER-2, as it shows comparable interaction dynamics relative to the clinically established lapatinib.

### 2.5 ADME studies

SwissADME was employed to predict the pharmacokinetic properties of compounds **5a** and reference drug lapatinib, focusing on their Absorption, Distribution, Metabolism, and Excretion (ADME) characteristics (Khaled et al., 2023). The analysis sheds light on their drug-like behaviors, identifying distinct features and shared attributes that could influence the performance **5a** as a dual EGFR/HER-2 inhibitor. The analysis can also guide its potential for further development.

The ADME profiles of compound **5a** and lapatinib reveal significant differences and similarities. Compound **5a** exhibits a lower molecular weight (369.29 g/mol) than lapatinib (581.06 g/mol), enhancing its likelihood of better absorption and permeability. Additionally, **5a** has fewer rotatable bonds (5 vs 11 for lapatinib), indicating reduced molecular flexibility, which may improve binding stability within the target sites. Regarding solubility, **5a** is moderately soluble, whereas lapatinib is poorly soluble, a key limitation that could impact its bioavailability. Both compounds show low gastrointestinal (GI) absorption. However, the higher polar surface area **5a** (TPSA: 169.12 Å^2^) than lapatinib (TPSA: 114.73 Å^2^) may limit its permeability.

Lapatinib, while clinically effective, inhibits several CYP450 enzymes (CYP2C19, CYP2C9, CYP2D6, and CYP3A4), indicating a higher risk for drug-drug interactions, whereas **5a** shows no CYP450 inhibition, highlighting its safer pharmacokinetic profile. Both compounds have comparable bioavailability scores (0.55), but the lower consensus LogP of **5a** (1.26 vs 5.19 for lapatinib) suggests better hydrophilicity, which may support its drug-likeness. Furthermore, **5a** demonstrates easier synthetic accessibility (score: 3.22) compared to lapatinib (score: 4.05), which could simplify manufacturing processes. A safer profile of **5a**, better solubility, and lack of CYP450 inhibition suggest it could be a promising alternative to lapatinib.

### 2.6 DFT analysis of compound **5a**

**FIGURE 18:**
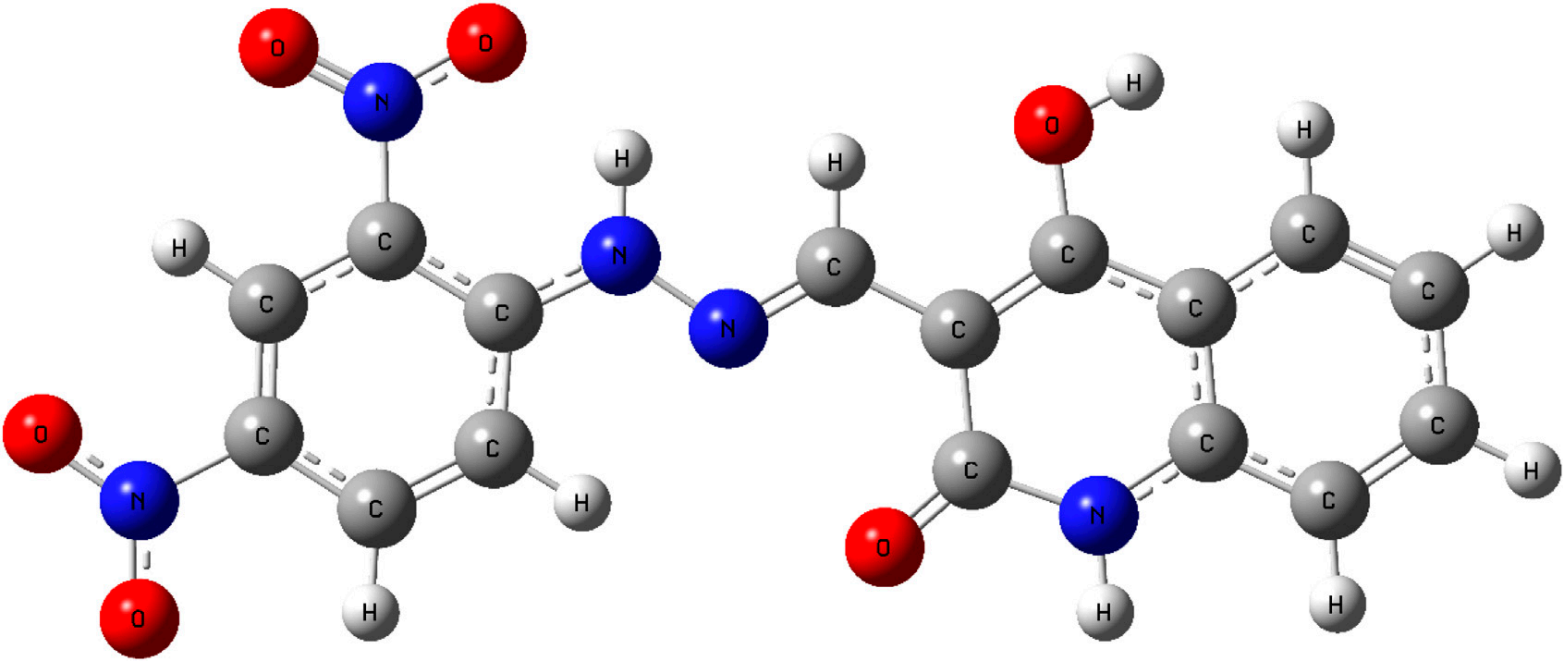
Optimized molecular geometry of compound **5a** at the B3LYP/6-311+G (d,p) level showing planarity and conjugation across the quinoline–hydrazone–di-nitrophenyl scaffold.

To gain deeper insight into the electronic characteristics and reactivity profile of compound **5a**, Density Functional Theory (DFT) calculations were carried out using Gaussian 09 software with visualizations generated through Gauss View 6.0 (Ozcelik et al., 2023). The geometry optimization and vibrational frequency analyses were performed at the B3LYP level of theory with the 6-311+G (d,p) basis set, which incorporates diffuse and polarization functions to capture electron distribution and non-covalent interactions (Ozcelik et al., 2023) more accurately. The optimized structure of compound **5a** was confirmed as a true energy minimum since no imaginary vibrational frequencies were detected (Janani et al., 2021). The calculated geometry reveals that the molecule adopts a nearly planar conformation across the quinoline–hydrazone–di-nitrophenyl scaffold, which facilitates extended π-conjugation and supports the intermolecular interactions observed in the molecular docking studies with EGFR and HER-2 (Figure 18).

**FIGURE 19:**
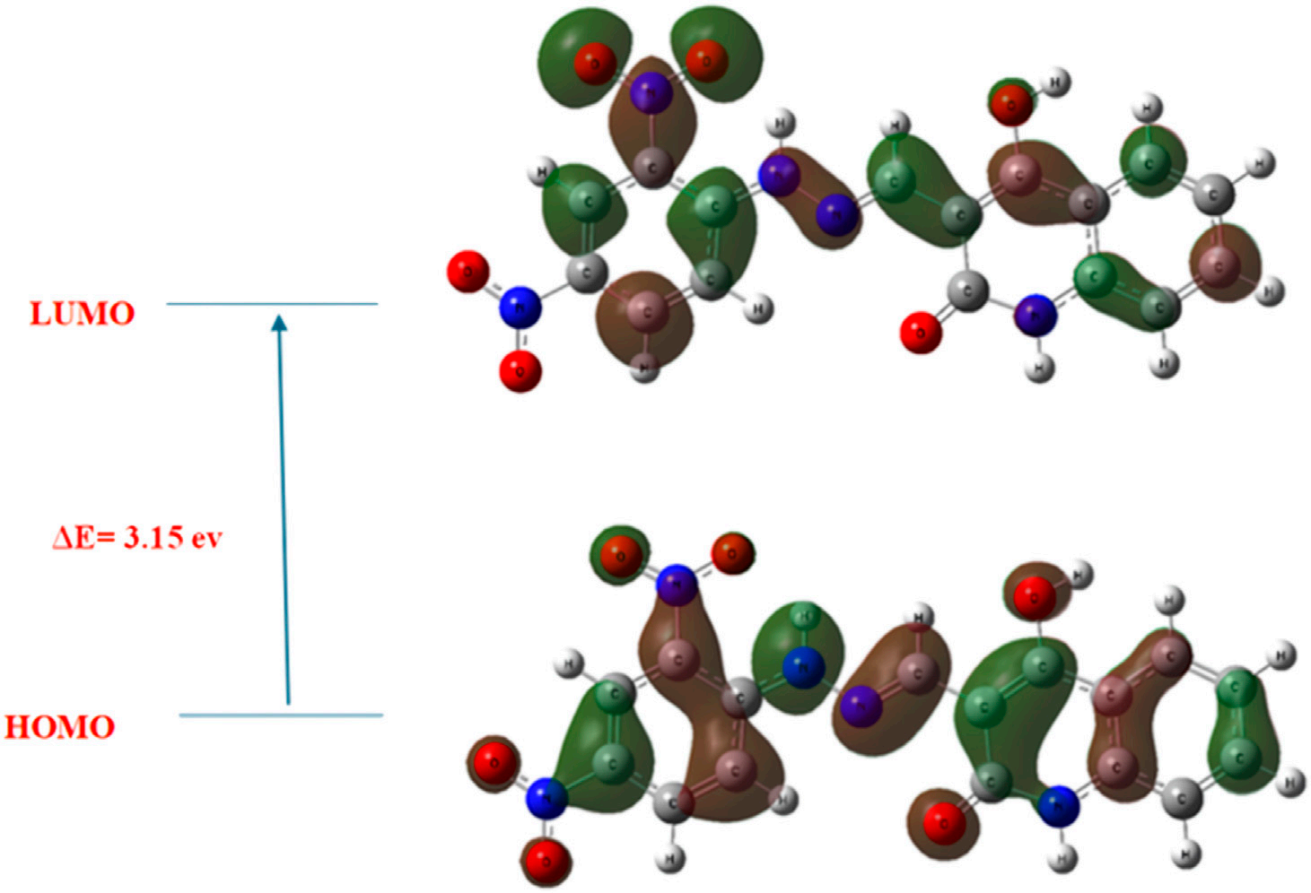
Frontier Molecular Orbitals (HOMO and LUMO) of compound **5a** with an energy gap (ΔE) of 3.15 eV, highlighting electron-rich (HOMO) and electron-deficient (LUMO) regions relevant to EGFR and HER-2 binding.

Frontier Molecular Orbital (FMO) analysis provided further evidence of the dual inhibitory potential of compound **5a** (Figure 19). The HOMO (Highest Occupied Molecular Orbital) was found to be located primarily over the quinoline core and hydrazone linker, indicating their role in electron donation during interaction with key amino acid residues, particularly those forming hydrogen bonds in the EGFR binding site (e.g., Met769 and Lys721). In contrast, the LUMO (Lowest Unoccupied Molecular Orbital) was predominantly located over the di-nitrophenyl ring, especially the nitro substituent, suggesting these regions function as electron acceptors and may be involved in stabilizing interactions with HER-2 residues such as Met801 and Thr862. The HOMO–LUMO energy gap (ΔE) was calculated as 3.15 eV, a moderate value indicating a balance between chemical stability and biological reactivity both of which are desirable properties for bioactive small molecules.

**FIGURE 20:**
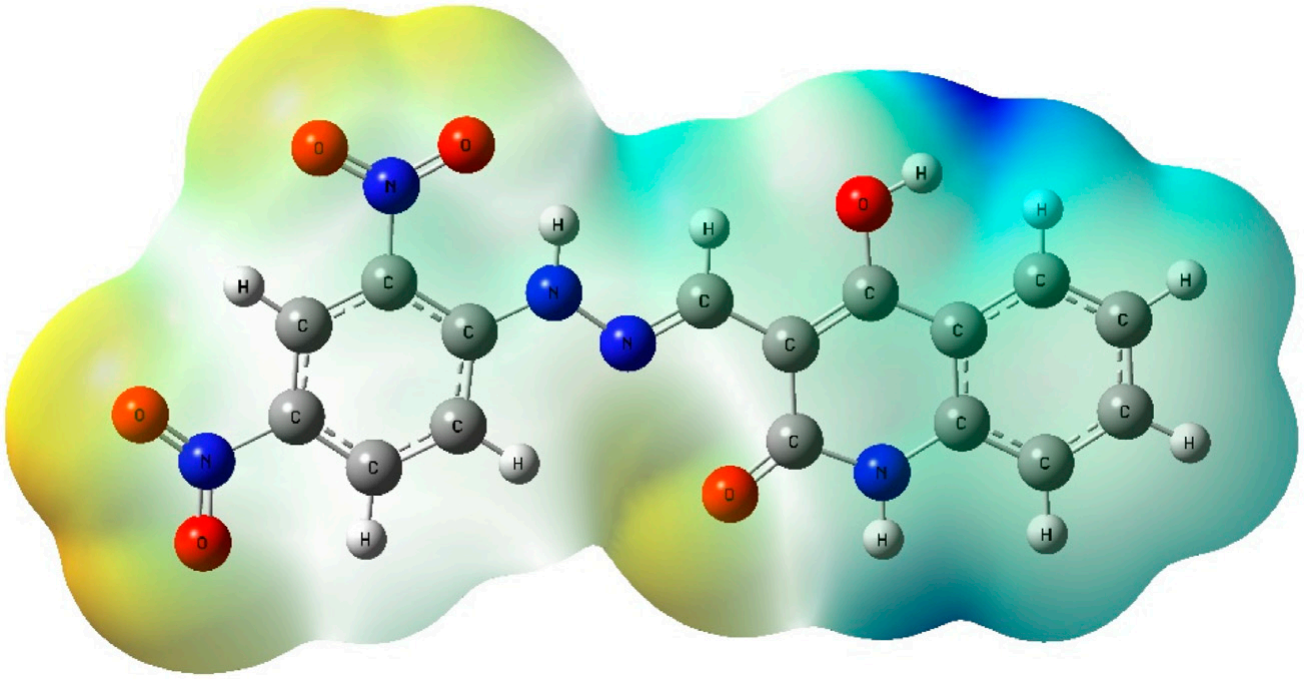
Molecular Electrostatic Potential (ESP) map of compound **5a** indicating zones of nucleophilic (red), electrophilic (blue), and neutral (green) potential character correlating with observed docking interactions in HER-2 and EGFR.

A molecular electrostatic potential (ESP) map was generated to complement the orbital analysis and visualize the distribution of electrostatic charges on the molecular surface. In this ESP surface, red regions indicate areas of highest negative electrostatic potential, blue regions signify the most positive potential, and green represents regions of neutral potential (Figure 20). The red zones were prominently located around the oxygen atoms of the nitro groups and carbonyl functionalities, marking them as favorable sites for hydrogen bond acceptance. Meanwhile, the blue zones were observed around the hydrazone NH and enol OH groups, which serve as potential hydrogen bond donors. These findings correlate closely with the docking results, where such groups engage in crucial hydrogen bonding interactions with residues in both EGFR and HER-2 active sites. Additionally, the extended green zones around the aromatic rings represent regions with relatively neutral electrostatic potential, supporting π-π stacking and hydrophobic interactions observed in the docking models with residues like Leu694 (EGFR) and Val734 (HER-2).

The electronic features derived from DFT calculations provide strong mechanistic support for compound 5a’s dual EGFR/HER-2 inhibitory activity. The HOMO-LUMO orbital distribution highlights the complementary donor-acceptor regions essential for stable protein-ligand interactions. At the same time, the ESP map reveals an electrostatic profile conducive to binding within the ATP-binding pockets of both kinases. Together, these DFT findings reinforce the docking-based conclusions and confirm the structural suitability of compound **5a** for further development as a dual-targeted anticancer agent.

## 3 Conclusion

This comprehensive study reports the successful synthesis, characterization, biological evaluation, and computational analysis of a new class of quinolin-2(1H)-one derivatives designed to function as dual inhibitors of EGFR and HER-2; two critical tyrosine kinases implicated in various cancers, including breast carcinoma. The lead compound, **5a**, demonstrated potent antiproliferative activity across multiple human cancer cell lines, with superior efficacy against the MCF-7 breast cancer cell line compared to the reference drug erlotinib. Enzymatic assays validated its dual-target inhibitory profile, exhibiting potent activity against EGFR and HER-2. Mechanistic investigations confirmed that compound **5a** promotes apoptosis through intrinsic and extrinsic pathways. Furthermore, **5a** induced G0/G1 phase arrest in MCF-7 cells, reinforcing its role in halting cancer cell proliferation. Molecular docking revealed that **5a** forms multiple stabilizing interactions within the active sites of both kinases. Molecular dynamics simulations over 100 ns confirmed the conformational stability of the ligand–protein complexes. Pharmacokinetic predictions from SwissADME indicated that **5a** exhibits favorable drug-like properties. DFT analysis further supported the electronic suitability of **5a** for dual kinase interaction. In summary, compound **5a** emerges as a compelling dual-target anticancer agent, combining potent antiproliferative efficacy, dual enzymatic inhibition, apoptosis induction, and favorable pharmacokinetic and electronic profiles. These results strongly support the continued preclinical development of **5a** as a lead compound for targeted therapy in EGFR/HER-2-overexpressing cancers.

## 4 Experimental

### 4.1 Chemistry

**General Details:** See Appendix A (Supplementary Material).

#### 4.1.1 Materials and methods

4-Hydroxy-2-oxo-1,2-dihydroquinoline-3-carbaldehydes **1a-h** (Tang and Shi, 2008), 4-methylbenzene-sulfonohydrazide (**2**) (Şenkardeş et al., 2020) were prepared as previously described. (2,4-Dinitrophenyl)hydrazine (**4**) was purchased from Sigma Aldrich.

#### 4.1.2 Synthesis of (*E*)-*N*'-((4-hydroxy-7-2-oxo-1,2-dihydroquinolin-3-yl) methylene)benzenesulfonohydrazides 3a-h and (*E*)-3-((2-(2,4-dinitrophenyl) hydrazono)methyl)-4-hydroxyquinolin-2(1*H*)-ones **5a-g**

In a round-bottomed flask, the appropriate quinolone-carbaldehydes **1a-h** (1 mmol) were dissolved in absolute ethanol (10 mL), to which a few drops of glacial acetic acid were added. A solution of 4-methylbenzenesulfonohydrazide (**2**, 186 mg) or (2,4-dinitrophenyl)hydrazine (**4**, 198 mg), dissolved in 10 mL of absolute ethanol, was added to the resultant mixture. The entire reaction was subjected to reflux for 4 hours. Following the completion of the reaction and the disappearance of the starting materials, the precipitates were collected and recrystallized from a mixture of ethanol/DMF in ratio (5:2) to yield the desired products **3a-h** and **5a-g** in good to excellent yields.

##### 4.1.2.1 (*E*)-*N'*-((4-hydroxy-1-methyl-2-oxo-1,2-dihydroquinolin-3-yl)methylene)-4-methylbenzenesulfonohydrazide (**3a**)

Yellow crystals, yield (83%), mp. 245–247 °C; IR (KBr): ν = 3286 (NH), 3012 (Ar-CH), 2857 (ali-CH), 1668 (C=O), 1609 (C=N), 1568 (Ar-C=C), 1344, 1163 (SO_2_) cm^−1^; ^1^H NMR (400 MHz, DMSO-*d*_*6*_): δ_H_ = 2.36 (s, 3H, CH_3_), 3.52 (s, 3H, CH_3_), 7.26–7.30 (m, 1H, Ar-H), 7.45–7.47 (d, 2H, *J* = 8 Hz, Ar-H), 7.46–7.48 (d, 1H, *J* = 8 Hz, Ar-H), 7.65–7.69 (m, 1H, Ar-H), 7.73–7.75 (d, 2H, *J* = 8, Ar-H), 7.95–7.98 (m, 1H, Ar-H), 8.36 (s, 1H, CH = N), 11.71 (br, s, 1H, hydrazono-NH), 12.90 (br, s, 1H, OH) ppm; ^13^C NMR (100 MHz, DMSO-*d*_*6*_): δ_C_ = 21.05 (CH_3_), 28.96 (CH_3_), 101.61 (C-3), 122.15, 124.02, 127.18, 130.10 (Ar-CH), 115.10, 133.16, 134.93, 140.06, 144.27 (Ar-C), 149.69 (CH = N), 160.60 (C-4) 164.47 (C=O) ppm; MS (*m/z*): 391 (M^+^ + H_2_O, 5),154 (100), 92 (13). *Anal. Calcd. For* C_18_H_17_N_3_O_4_S (371.41): C, 58.21; H, 4.61; N, 11.31; S, 8.63. Found: C, 58.16; H, 4.74; N, 11.33; S, 8.58.

##### 4.1.2.2 (*E*)-*N*'-((4-hydroxy-6-methoxy-2-oxo-1,2-dihydroquinolin-3-yl) methylene)-4-methyl benzenesulfonohydrazide (**3b**)

Yellow crystals, yield (90%), mp. 272–273 °C; IR (KBr): ν = 3275 (NH), 3004 (Ar-CH), 2948, 2881 (ali-CH), 1662 (C=O), 1602 (C=N), 1566, 1492 (Ar-C=C), 1416, 1240 (SO_2_) cm^−1^; ^1^H NMR (400 MHz, DMSO-*d*_*6*_): δ_H_ = 2.37 (s, 3H, CH_3_), 3.79 (s, 3H, OCH_3_), 7.20–7.26 (m, 3H, Ar-H), 7.45–7.47 (d, 2H, *J* = 8, Ar-H), 7.73–7.75 (d, 2H, *J* = 8, Ar-H), 8.30 (s, 1H, CH = N), 11.37 (s, 1H, quinolone-NH) 11.68 (br, s, 1H, hydrazono-NH), 12.91 (br, s, 1H, OH) ppm; ^13^C NMR (100 MHz, DMSO-*d*_*6*_): δ_C_ = 21.07 (CH_3_), 55.48 (OCH_3_), 102.13 (C-3), 104.23, 114.71, 117.25, 122.51, 127.16, 130.13 (Ar-CH), 133.90, 134.94, 144.42 (Ar-C), 149.49 (CH = N), 154.29 (C-OMe), 160.88 (C-4) 165.88 (C=O) ppm; MS (*m/z*): 391 (M^+^, 5), 220 (5), 190 (14), 154 (100), 92 (13). Anal. Calcd. For C_18_H_17_N_3_O_5_S (387.41): C, 55.80; H, 4.42; N, 10.85; S, 8.28. Found: C, 55.77; H, 4.37; N, 10.87; S, 8.25.

##### 4.1.2.3 (*E*)-*N*'-((4-hydroxy-6-methyl-2-oxo-1,2-dihydroquinolin-3-yl)methylene)-4-methylbenzenesulfonohydrazide (**3c**)

Yellow crystals, yield (92%), mp. 280–282 °C; IR (KBr): ν = 3196 (NH), 3104 (Ar-CH), 2915 (ali-CH), 1670 (C=O), 1610 (C=N), 1591, 1463 (Ar-C=C), 1404, 1159 (SO_2_) cm^−1^; ^1^H NMR (400 MHz, DMSO-*d*_*6*_): δ_H_ = 2.33 (s, 3H, CH_3_), 2.38 (s, 3H, CH_3_), 7.14–7.25 (m, 2H, Ar-H), 7.46–7.48 (d, 2H, *J* = 8, Ar-H), 7.55–7.59 (m, 1H, Ar-H), 7.74–7.76 (d, 2H, *J* = 8, Ar-H), 8.31 (s, 1H, CH = N), 11.47 (s, 1H, quinolone-NH) 11.66 (br, s, 1H, hydrazono-NH), 12.88 (br, s, 1H, OH) ppm; ^13^C NMR (100 MHz, DMSO-*d*_*6*_): δ_C_ = 20.55 (CH_3_), 21.08 (CH_3_), 98.26 (C-3), 114.90, 122.18, 125.54, 127.67, 129.30 (Ar-CH), 130.11, 132.05, 136.13, 137.22, 143.21 (Ar-C), 162.32 (CH = N) 163.49 (C-4), 167.98 (C=O) ppm; MS (*m/z*): 371 (M^+^, 20), 201 (4), 174 (10), 154 (100), 92 (17). Anal. Calcd. For C_18_H_17_N_3_O_4_S (371.41): C, 58.21; H, 4.61; N, 11.31; S, 8.63. Found: C, 58.13; H, 4.57; N, 11.33; S, 8.59.

##### 4.1.2.4 (*E*)-*N*'-((4-hydroxy-2-oxo-1,2-dihydroquinolin-3-yl)methylene)-4-methyl benzenesulfonohydrazide (**3d**)

Yellow crystals, yield (85%), mp. 255–257 °C; IR (KBr): ν = 3201 (NH), 3011 (Ar-CH), 2952, 2833 (ali-CH), 1656 (C=O), 1613 (C=N), 1591, 1493 (Ar-C=C), 1404, 1159 (SO_2_) cm^−1^; ^1^H NMR (400 MHz, DMSO-*d*_*6*_): δ_H_ = 2.38 (s, 3H, CH_3_), 7.18–7.22 (t, 1H, *J* = 16 Hz, Ar-H), 7.25–7.27 (d, 1H, *J* = 8 Hz, Ar-H), 7.46–7.48 (d, 2H, *J* = 8 Hz, Ar-H), 7.55–7.58 (t, 1H, *J* = 12 Hz, Ar-H), 7.75–7.77 (d, 2H, *J* = 8 Hz, Ar-H), 7.86–7.88 (d, 1H, *J* = 8 Hz, Ar-H), 8.33 (s, 1H, CH = N), 11.48 (s, 1H, quinolone-NH) 11.80 (br, s, 1H, hydrazono-NH), 12.91 (br, s, 1H, OH) ppm; ^13^C NMR (100 MHz, DMSO-*d*_*6*_): δ_C_ = 21.65 (CH_3_), 98.29 (C-3), 114.90, 115.68, 122.01, 123.75, 127.24, 130.12 (Ar-CH), 102.01, 132.93, 139.46, 144.29 (Ar-C), 149.69 (CH = N) 161.38 (C-4), 166.64 (C=O) ppm; MS (*m/z*): 357 (M^+^, 6), 188 (4), 154 (100), 92 (16). Anal. Calcd. For C_17_H_15_N_3_O_4_S (357.38): C, 57.13; H, 4.23; N, 11.76; S, 8.97. Found: C, 55.08; H, 4.30; N, 11.78; S, 8.91.

##### 4.1.2.5 (*E*)-*N*'-((1-Ethyl-4-hydroxy-2-oxo-1,2-dihydroquinolin-3-yl)methylene)-4-methyl benzenesulfonohydrazide (**3e**)

Yellow crystals, yield (82%), mp. 265–267 °C; IR (KBr): ν = 3222 (NH), 3066 (Ar-CH), 2977, 2872 (ali-CH), 1673 (C=O), 1607 (C=N), 1566, 1499 (Ar-C=C), 1335, 1165 (SO_2_) cm^−1^; ^1^H NMR (400 MHz, DMSO-*d*_*6*_): δ_H_ = 1.13–1.16 (t, 3H, *J* = 12, CH_3_), 2.36 (s, 3H, CH_3_), 4.16–4.19 (q, 2H, *J* = 12 Hz, CH_2_), 7.25–7.29 (t, 1H, *J* = 12 Hz, Ar-H), 7.45–7.47 (d, 2H, *J* = 8 Hz, Ar-H), 7.49–7.52 (d, 1H, *J* = 12, Ar-H), 7.65–7.69 (t, 3H, *J* = 16 Hz, Ar-H), 7.75–7.77 (d, 2H, J = 8 Hz, Ar-H), 7.97–7.99 (d, 1H, *J* = 8 Hz, Ar-H), 8.38 (s, 1H, CH = N), 11.74 (br, s, 1H, hydrazono-NH), 12.91 (br, s, 1H, OH) ppm; ^13^C NMR (100 MHz, DMSO-*d*_*6*_): δ_C_ = 12.71 (CH_3_), 21.05 (CH_3_), 36.46 (CH_2_), 101.54 (C-3), 115.21, 122.04, 124.28, 127.18, 130.10, 133.23 (Ar-CH), 114.81, 134.96, 138.96, 144.25 (Ar-C), 149.42 (CH = N), 160.16 (C-4), 164.15 (C=O) ppm. MS (*m/z*): 385 (M^+^, 9), 154 (100), 92 (18). Anal. Calcd. For C_19_H_19_N_3_O_4_S (385.44): C, 59.21; H, 4.97; N, 10.90; S, 8.32. Found: C, 59.17; H, 4.89; N, 10.87; S, 8.27.

##### 4.1.2.6 (*E*)-*N*'-((6-chloro-4-hydroxy-2-oxo-1,2-dihydroquinolin-3-yl)methylene)-4-methyl benzenesulfonohydrazide (**3f**)

Yellow crystals, yield (79%), mp. 270–272 °C; IR (KBr): ν = 3230 (NH), 3047 (Ar-CH), 2900, 2823 (ali-CH), 1665 (C=O), 1595 (C=N), 1589, 1459 (Ar-C=C), 1410, 1187 (SO_2_) cm^−1^; ^1^H NMR (400 MHz, DMSO-*d*_*6*_): δ_H_ = 2.39 (s, 3H, CH_3_), 7.24–7.26 (d, 1H, *J* = 8 Hz, Ar-H), 7.46–7.48 (d, 2H, *J* = 8 Hz, Ar-H), 7.59–7.62 (dd, 1H, J = 12 Hz, Ar-H), 7.73–7.75 (d, 2H, *J* = 8 Hz, Ar-H), 7.81–7.83 (d, 1H, *J* = 8 Hz, Ar-H), 8.28 (s, 1H, CH = N), 11.56 (s, 1H, quinolone-NH) 11.68 (br, s, 1H, hydrazono-NH), 12.86 (br, s, 1H, OH) ppm; ^13^C NMR (100 MHz, DMSO-*d*_*6*_): δ_C_ = 21.09 (CH_3_), 102.56 (C-3), 116.15, 122.77, 126.06, 127.28, 130.15 (Ar-CH), 117.76, 132.77, 134.69, 138.24, 144.41 (Ar-C), 150.14 (CH = N) 161.28 (C-4), 166.36 (C=O) ppm; MS (*m/z*): 371 (M^+^, 100), 201 (21), 188 (30), 154 (100), 91 (4). Anal. Calcd. For C_17_H_14_ClN_3_O_4_S (391.83): C, 52.11; H, 3.60; Cl, 9.05; N, 10.72; S, 8.18. Found: C, 52.07; H, 3.52; Cl, 9.00; N, 10.65; S, 8.20.

##### 4.1.2.7 (*E*)-*N*'-((4-hydroxy-7-methyl-2-oxo-1,2-dihydroquinolin-3-yl)methylene)-4-methyl benzenesulfonohydrazide (**3g**)

Yellow crystals, yield (91%), mp. 224–225 °C; IR (KBr): ν = 3642/3519 (OH), 3182 (NH), 3029 (Ar-CH), 2977, 2864 (ali-CH), 1642 (C=O), 1597 (C=N), 1497, 1484 (Ar-C=C), 1382, 1163 (SO_2_) cm^−1^; ^1^H NMR (400 MHz, DMSO-*d*_*6*_): δ_H_ = 2.34 (s, 3H, CH_3_), 3.17 (s, 3H, CH_3_), 6.90–6.92 (d, 1H, *J* = 8, Ar-H), 7.07–7.09 (d, 1H, *J* = 8 Hz, Ar-H), 7.35–7.38 (t, 1H, *J* = 12 Hz, Ar-H), 7.45–7.47 (d, 2H, *J* = 8 Hz, Ar-H), 7.73–7.75 (d, 2H, *J* = 8 Hz, Ar-H), 8.30 (s, 1H, CH = N), 11.38 (br, s, 1H, quinolone-NH), 11.50 (br, s, 1H, hydrazono-NH), 13.14 (br, s, 1H, OH) ppm; ^13^C NMR (100 MHz, DMSO-*d*_*6*_): δ_C_ = 21.52 (CH_3_), 23.42 (CH_3_), 102.15 (C-3), 114.01, 115.35, 125.18, 127.24, 127.34, (Ar-CH), 114.19, 130.11, 132.38, 138.34, 151.47 (Ar-C), 159.63 (CH = N), 161.57 (C-4), 166.77 (C=O) ppm; Anal. Calcd. For C_18_H_17_N_3_O_4_S (371.41): C, 58.21; H, 4.61; N, 11.31; S, 8.63. Found: C, 58.13; H, 4.57; N, 11.33; S, 8.59.

##### 4.1.2.8 (*E*)-*N*'-((4-hydroxy-8-methyl-2-oxo-1,2-dihydroquinolin-3-yl)methylene)-4-methyl benzenesulfonohydrazide (**3h**)

Yellow crystals, yield (90%), mp. 234–236 °C; ^1^H NMR (400 MHz, DMSO-*d*_*6*_): δ_H_ = 1.90 (s, 3H, CH_3_), 2.37 (s, 3H, CH_3_), 7.09–7.13 (t, 1H, *J* = 16 Hz, Ar-H), 7.41–7.43 (d, 1H, *J* = 8 Hz, Ar-H), 7.45–7.47 (d, 2H, *J* = 8 Hz, Ar-H), 7.74–7.76 (d, 3H, *J* = 8 Hz, Ar-H), 8.32 (s, 1H, CH = N), 11.69 (br, s, 1H, quinolone-NH), 11.95 (br, s, 1H, hydrazono-NH), 12.88 (br, s, 1H, OH) ppm; ^13^C NMR (100 MHz, DMSO-*d*_*6*_): δ_C_ = 17.40 (CH_3_), 21.10 (CH_3_), 101.72 (C-3), 121.55, 121.77, 124.07, 127.19, 130.14 (Ar-CH), 134.05, 134.90, 137.96, 144.29, 149.61 (Ar-C), 161.64 (CH = N), 166.22 (C-4), 172.09 (C=O) ppm; *Anal. Calcd. For* C_18_H_17_N_3_O_4_S (371.41): C, 58.21; H, 4.61; N, 11.31; S, 8.63. Found: C, 58.23; H, 4.55; N, 11.28; S, 8.60.

##### 4.1.2.9 (*E*)-3-((2-(2,4-Dinitrophenyl)hydrazono)methyl)-4-hydroxyquinolin-2(1*H*)-one (**5a**)

Orange crystals, yield (86%), mp. = 327–329 °C; ^1^H NMR (400 MHz, DMSO-*d*_*6*_): δ_H_ = 7.18–7.20 (d, *J* = 8.0 Hz, 2H, quinolinone-H), 7.22–7.77 (m, 3H, quinolinone-H, Ar-H), 7.89–7.91 (d, 2H, *J* = 8.0 Hz, dinitrobenzene-H), 8.35 (s, 1H, CH = N), 11.18 (br, s, 1H, quinolone-NH), 11.33 (br, s, 1H, hydrazono-NH), 12.77 (br, s, 1H, OH) ppm; ^13^C NMR (100 MHz, DMSO-*d*_*6*_): δ_C_ = 101.10 (C-3), 115.10, 121.16, 124.03, 126.19, 130.11, 132.16, 134.06 (Ar-CH), 140.06, 144.28 (Ar-C), 149.69 (CH = N) 161.10 (C-4), 166.43 (C=O) ppm. *Anal. Calcd. For* C_16_H_11_N_5_O_6_ (369.29): C, 52.04; H, 3.00; N, 18.96. Found: C, 51.97; H, 3.05; N, 18.00.

##### 4.1.2.10 (*E*)-3-((2-(2,4-Dinitrophenyl)hydrazono)methyl)-4-hydroxy-6-methyl quinolin-2(1*H*)-one (**5b**)

Orange crystals, yield (89%), mp. 338–340 °C; ^1^H NMR (400 MHz, DMSO-*d*_*6*_): δ_H_ = 2.54 (s, 3H, CH_3_), 7.18–7.41 (m, 4H, quinolinone-H, Ar-H), 7.42–7.43 (d, 2H, quinolinone-H, Ar-H), 8.74 (s, 1H, CH = N), 11.19 (br, s, 1H, quinolone-NH), 11.62 (br, s, 1H, hydrazono-NH), 12.69 (br, s, 1H, OH) ppm; *Anal. Calcd. For* C_17_H_13_N_5_O_6_ (383.32): C, 53.27; H, 3.42; N, 18.27. Found: C, 53.19; H, 3.45; N, 18.18.

##### 4.1.2.11 (*E*)-3-((2-(2,4-Dinitrophenyl)hydrazono)methyl)-4-hydroxy-6-methoxy quinolin-2(1*H*)-one (**5c**)

Orange red crystals, yield (90%), mp. 313–315 °C; ^1^H NMR (400 MHz, DMSO-*d*_*6*_): δ_H_ = 3.84 (s, 3H, OCH_3_), 7.20–7.43 (m, 6H, quinolinone-H, Ar-H), 8.43 (s, 1H, CH = N), 11.20 (br, s, 1H, quinolone-NH), 11.60 (br, s, 1H, hydrazono-NH), 12.93 (br, s, 1H, OH) ppm; *Anal. Calcd. For* C_17_H_13_N_5_O_6_ (399.31): C, 51.13; H, 3.28; N, 17.54. Found: C, 51.05; H, 3.30; N, 17.49.

##### 4.1.2.12 (*E*)-3-((2-(2,4-Dinitrophenyl)hydrazono)methyl)-4-hydroxy-8-methyl quinolin-2(1*H*)-one (**5d**)

Orange red crystals, yield (87%), mp. 338–340 °C; ^1^H NMR (400 MHz, DMSO-*d*_*6*_): δ_H_ = 1.91 (s, 3H, CH_3_), 7.19–7.49 (m, 2H, Ar-H), 7.62–7.71 (m, 2H, Ar-H), 7.87–7.95 (m, 1H, Ar-H), 8.28–8.48 (m, 1H, Ar-H), 8.83–8.90 (d, 1H, Ar-H), 9.19 (s, 1H, CH = N), 10.84 (br, s, 1H, quinolone-NH), 11.95 (br, s, 1H, hydrazono-NH), 12.99 (br, s, 1H, OH) ppm; *Anal. Calcd. For* C_17_H_13_N_5_O_6_ (383.32): C, 53.27; H, 3.42; N, 18.27. Found: C, 53.17; H, 3.45; N, 18.19.

##### 4.1.2.13 (*E*)-3-((2-(2,4-Dinitrophenyl)hydrazono)methyl)-4-hydroxy-1-methyl quinolin-2(1*H*)-one (**5e**)

Orange crystals, yield (77%), mp. 295–297 °C; ^1^H NMR (400 MHz, DMSO-*d*_*6*_): δ_H_ = 3.62 (s, 3H, NCH_3_), 7.36–7.74 (m, 3H, Ar-H), 8.07–8.11 (m, 2H, Ar-H), 8.44–8.46 (d, 1H, *J* = 8.4 Hz, Ar-H), 8.86–8.92 (d, 1H, Ar-H), 9.20 (s, 1H, CH = N), 11.88 (br, s, 1H, hydrazono-NH), 12.67 (br, s, 1H, OH) ppm; 13C NMR (100 MHz, DMSO-d6): δ_C_ = 28.83 (NCH3), 114.79, 115.21, 115. 31, 121.92, 123.69, 129.82 (Ar-CH), 129.91, 132.92, 137.24 (Ar-C), 149.98 (CH = N), 161.09 (C-4), 164.31 (C=O) ppm; *Anal. Calcd. For* C_17_H_13_N_5_O_6_ (383.32): C, 53.27; H, 3.42; N, 18.27. Found: C, 53.29; H, 3.38; N, 18.31.

##### 4.1.2.14 (*E*)-3-((2-(2,4-Dinitrophenyl)hydrazono)methyl)-4-hydroxy-7-methyl quinolin-2(1*H*)-one (5f)

Orange red crystals, yield (88%), mp. 332–334 °C; ^1^H NMR (400 MHz, DMSO-*d*_*6*_): δ_H_ = 3.00 (s, 3H, CH_3_), 7.01–7.39 (m, 6H, quinolinone-H, Ar-H), 8.42 (s, 1H, CH = N), 11.17 (br, s, 1H, quinolone-NH), 11.41 (br, s, 1H, hydrazono-NH), 12.76 (br, s, 1H, OH) ppm; *Anal. Calcd. For* C_17_H_13_N_5_O_6_ (383.32): C, 53.27; H, 3.42; N, 18.27. Found: C, 53.29; H, 3.38; N, 18.30.

##### 4.1.2.15 (*E*)-6-Chloro-3-((2-(2,4-dinitrophenyl)hydrazono)methyl)-4-hydroxy quinolin-2(1*H*)-one (**5g**)

Orange red crystals, yield (76%), mp. 328–330 °C; ^1^H NMR (400 MHz, DMSO-*d*_*6*_): δ_H_ = 7.23–7.43 (m, 6H, quinolinone-H, Ar-H), 8.73 (s, 1H, CH = N), 11.22 (br, s, 1H, quinolone-NH), 11.77 (br, s, 1H, hydrazono-NH), 12.97 (br, s, 1H, OH) ppm; *Anal. calcd. for* C_16_H_10_ClN_5_O_6_ (403.73): C, 47.60; H, 2.50; Cl, 8.78; N, 17.35. Found: C, 47.62; H, 2.48; Cl, 8.71; N, 17.38.

#### 4.1.3 Crystal X-ray structure determination of 3g

Single crystals of **3g** were obtained by recrystallization from CH_3_CH_2_OH. The single-crystal X-ray diffraction study was carried out on a Bruker D8 Venture diffractometer with a Photon II detector at 173 (2) K (λ = 1.54178 Å). Dual space methods (SHELXT) (Sheldrick, 2015) were used for structure solution, and refinement was carried out using SHELXL-2014 (full-matrix least-squares on F2). Hydrogen atoms were refined using a riding model (H(N, O) free). A semi-empirical absorption correction was applied. The methyl group is disordered (5-methyl vs 7-methyl, approximately 73:27; see cif-file for details).

Compound **3g**: C_18_H_17_N_3_O_4_S·H_2_O, Mr = 389.42 g mol^−1^, yellow crystals, size 0.20 × 0.12 × 0.08 mm, Monoclinic, *P*2_1_/*n (no.14)*, a = 7.8378 (5) Å, b = 9.1161 (6) Å, c = 26.7859 (17) Å, β = 97.797 (2)°, V = 1896.2 (2) Å^3^, λ = 1.54178 Å, Z = 4, D_calcd_ = 1.364 Mg m^−3^, *F* (000) = 816, µ = 1.82 mm^−1^, T = 173 K, 21,753 collected reflection (2θ_max_ = 144.0°), of which 3729 were reflection unique (R_int_ = 0.030), 261 parameters, 216 restraints, R1 [for 3401 I > 2σ(I)] = 0.043, wR2 (for all data) = 0.125, S = 1.03, largest diff. peak and hole = 0.30 e Å^−3^/-0.33 e Å^−3^. CCDC 2451182 (**3g**) contains the supplementary crystallographic data for this paper. These data can be obtained free from The Cambridge Crystallographic Data Centre *via* www.ccdc.cam.ac.uk/data_request/cif.

### 4.2 Biology

#### 4.2.1 Cell viability assay

The MTT assay was used to assess the viability of **3a-h** and **5a-g** cells after 4 days of incubation with MCF-10A (a normal human mammary gland cell line) (El-Sherief et al., 2019; Ramadan et al., 2020). For more experimental details, refer to Supplementary Appendix A.

#### 4.2.2 Antiproliferative assay

Compounds **3a-h** and **5a-g** were tested for their antiproliferative activities on four human cancer cell lines: colon (HT-29), pancreatic (Panc-1), lung (A-549), and breast (MCF-7) using the MTT assay (Al-Wahaibi et al., 2022; Mahmoud et al., 2023). Erlotinib was applied as a reference. See Supplementary Appendix A for more experimental details.

#### 4.2.3 EGFR inhibitory assay

The most efficient antiproliferative derivatives, **3c**, **3d**, **3f**, **5a,** and **5b,** were evaluated for their ability to inhibit EGFR using the EGFR-TK test, with Erlotinib as the reference drug (Abou‐Zied et al., 2023). Refer to Supplementary Appendix A for more details.

#### 4.2.4 HER-2 inhibitory assay

Compounds **3c**, **3d**, **3f**, **5a,** and **5b** were evaluated for their capacity to inhibit HER-2 by a kinase assay (Al-Wahaibi et al., 2025). Lapatinib functioned as the reference drug. See Supplementary Appendix A for more experimental details.

#### 4.2.5 Caspases-3, -8, and -9 activation assay

The MCF-7 human breast cancer cell line was acquired from ATCC. RPMI 1640 with 10% FBS was used to assist the cells grow at 37 °C, and then the cells were treated with the compounds being studied to check the activity of caspase-3, -8, and -9 (Hisham et al., 2019). Refer to Supplementary Appendix A.

#### 4.2.6 Evaluation of Bax and Bcl-2 levels

RNA isolation was performed using the RNeasy extraction kit with up to 1 × 10^7 cells. They were disturbed and homogenized in Buffer RLT (Mitupatum et al., 2016). See Supplementary Appendix A for more details.

#### 4.2.7 Cytochrome C assay

Cells were obtained from the American Type Culture Collection and grown at 37 °C in RPMI 1640 supplemented with 10% fetal bovine serum before being stimulated with **3d** and **5a** to test cytochrome C (Abdelbaset et al., 2019). Refer to Supplementary Appendix A for more details on the experimental process.

#### 4.2.8 Flow cytometry and cell cycle analysis

Apoptosis was detected using flow cytometry with an annexin-V-fluorescein isothiocyanate (FITC) and propidium iodide (PI) staining kit (BD Pharmingen, San Diego, United States) (Mohamed et al., 2024). See Supplementary Appendix A for more details.

## Data Availability

The original contributions presented in the study are included in the article/Supplementary Material, further inquiries can be directed to the corresponding authors.
