# Correction: Design, synthesis, antiproliferative assessments, and computational studies of new quinolin-2(1*H*)-ones as dual EGFR/HER-2 inhibitors

**DOI:** 10.3389/fchem.2025.1748491

**Published:** 2025-12-10

**Authors:** Lamya H. Al-Wahaibi, Hesham A. Abou-Zied, Martin Nieger, Stefan Bräse, Bahaa G. M. Youssif, Hendawy N. Tawfeek

**Affiliations:** 1 Department of Chemistry, College of Sciences, Princess Nourah bint Abdulrahman University, Riyadh, Saudi Arabia; 2 Medicinal Chemistry Department, Faculty of Pharmacy, Deraya University, Minia, Egypt; 3 Department of Chemistry, University of Helsinki, Helsinki, Finland; 4 Institute of Biological and Chemical Systems, IBCS-FMS, Karlsruhe Institute of Technology, Karlsruhe, Germany; 5 Department of Pharmaceutical Organic Chemistry, Faculty of Pharmacy, Assiut University, Assiut, Egypt; 6 Chemistry Department, Faculty of Science, Minia University, El-Minia, Egypt; 7 Unit of Occupational of Safety and Health, Administration Office of Minia University, El-Minia, Egypt

**Keywords:** quinoline, kinases, X-ray, DFT, anticancer, EGFR, HER-2

An incorrect **Funding** statement was provided. The funding details were erroneously given in the **Acknowledgments** statement. The correct **Funding** statement reads:

The authors declare that financial support was received for the research and/or publication of this article. This work was funded by Princess Nourah bint Abdulrahman University Researchers Supporting Project Number (PNURSP2025R3), Princess Nourah bint Abdulrahman University, Riyadh, Saudi Arabia, and the KIT-Publication Fund of the Karlsruhe Institute of Technology.

The middle initial for author Hendawy N. Tawfeek was missing and this author’s surname was also misspelled as “Tawfik”.

The original article has been updated.

