# Supplementary material for: Design, synthesis, antiproliferative assessments, and computational studies of new quinolin-2(1H)-ones as dual EGFR/HER-2 inhibitors
Source: Front Chem. 2025 Sep 16;13:1638489. doi: 10.3389/fchem.2025.1638489 (PMC12481029; doi:10.3389/fchem.2025.1638489)
Supplement: Supplementary file 1 [file DataSheet1.docx]

**Design, synthesis, antiproliferative assessments, and computational studies of new** **quinolin-2(1*H*)-ones as dual EGFR/HER-2 inhibitors**

Lamya H. Al-Wahaibi1, Hesham A. Abou-Zied2, Martin Nieger3, Stefan Bräse4, Bahaa G. M. Youssif5, Hendawy Tawfik6,7

1Department of Chemistry, College of Sciences, Princess Nourah bint Abdulrahman University, Riyadh 11671, Saudi Arabia; 2Medicinal Chemistry Department, Faculty of Pharmacy, Deraya University, Minia, Egypt; 3Department of Chemistry, University of Helsinki, PO Box 55, A. I. Virtasen Aukio 1, 00014, Helsinki, Finland, 4Institute of Biological and Chemical Systems, IBCS-FMS, Karlsruhe Institute of Technology, 76131 Karlsruhe, Germany; 5Department of Pharmaceutical Organic Chemistry, Faculty of Pharmacy, Assiut University, Assiut 71526, Egypt,  6Chemistry Department, Faculty of Science, Minia University, El Minia, 61519 Egypt; 7Unit of Occupational of Safety and Health, Administration Office of Minia University, El-Minia 61519, Egypt

**To whom correspondence should be addressed:*

**Bahaa G. M. Youssif**, Ph.D. Pharmaceutical Organic Chemistry Department, Faculty of Pharmacy, Assiut University, Assiut 71526, Egypt.

**Tel**.: (002)-01098294419

***E-mail* address**: [bgyoussif2@gmail.com](mailto:bgyoussif2@gmail.com)

**Stefan Bräse**

Institute of Biological and Chemical Systems, IBCS-FMS, Karlsruhe Institute of Technology, 76131 Karlsruhe, Germany. ***e-mail***: [braese@kit.edu](mailto:braese@kit.edu)

***Crystal X-ray structure determination of* *3g***

Single crystals of **3g** were obtained by recrystallization from CH3CH2OH.The single-crystal X-ray diffraction study was carried out on a Bruker D8 Venture diffractometer with a PhotonII detector at 173(2) K (λ = 1.54178 Å). Dual space methods (SHELXT) [G. M. Sheldrick, Acta Crystallogr. 2015, **A71**, 3-8] were used for structure solution and refinement was carried out using SHELXL-2014 (full-matrix least-squares on F2) [G. M. Sheldrick, Acta Crystallogr. 2015, **C71**, 3-8]. Hydrogen atoms were refined using a riding model (H(N, O) free). A semi-empirical absorption corrections was applied. The methyl group is disordered (5-methyl vs. 7-methyl, approx. ratio 73:27, see cif-file for details).

CCDC 2451182 (**3g**) contains the supplementary crystallographic data for this paper. These data can be obtained free of charge from The Cambridge Crystallographic Data Centre via [www.ccdc.cam.ac.uk/data_request/cif](http://www.ccdc.cam.ac.uk/data_request/cif).








**Figure 1**. Molecular structure of **3g** (top: methylgroup at C7 with approx. 27% occupation; bottom: methylgroup at C5 with approx. 73% occupation. displacement parameters are drawn at 30% probability level)

**Table 1.** *Crystal data for* ***3g***

| C18H17N3O4S·H2O | *F*(000) = 816 |
| --- | --- |
| *Mr* = 389.42 | *D*x = 1.364 Mg m-3 |
| Monoclinic, *P*21/*n (no.14)* | Cu *K*α radiation,  = 1.54178 Å |
| *a* = 7.8378 (5) Å | Cell parameters from 9865 reflections |
| *b* = 9.1161 (6) Å |  = 3.3–72.0° |
| *c* = 26.7859 (17) Å |  = 1.82 mm-1 |
| β = 97.797 (2)° | *T* = 173 K |
| *V* = 1896.2 (2) Å3 | Blocks, yellow |
| *Z* = 4 | 0.20 × 0.12 × 0.08 mm |

**Table 2.** *Data collection for* ***3g***

| Bruker D8 VENTURE diffractometer with PhotonII CPAD detector | 3401 reflections with *I* > 2σ(*I*) |
| --- | --- |
| Radiation source: fine-focus sealed tube, IμS microfocus | *R*int = 0.030 |
| rotation in  and , 1°, shutterless scans | max = 72.0°, min = 3.3° |
| Absorption correction: multi-scan  *SADABS2016*/2 (Krause, L., Herbst-Irmer, R., Sheldrick G.M. & Stalke D., J. Appl. Cryst. 48 (2015) 3-10) | *h* = -99 |
| *T*min = 0.753, *T*max = 0.889 | *k* = -1110 |
| 21753 measured reflections | *l* = -3133 |
| 3729 independent reflections |  |

**Table 3.** *Refinement for* ***3g***

| Refinement on *F*2 | Primary atom site location: dual |
| --- | --- |
| Least-squares matrix: full | Secondary atom site location: difference Fourier map |
| *R*[*F*2 > 2σ(*F*2)] = 0.043 | Hydrogen site location: mixed |
| *wR*(*F*2) = 0.125 | H atoms treated by a mixture of independent and constrained refinement |
| *S* = 1.03 | *w* = 1/[2(*F*o2) + (0.0664*P*)2 + 0.7*P*]  where *P* = (*F*o2 + 2*F*c2)/3 |
| 3729 reflections | (Δ/σ)max = 0.001 |
| 261 parameters | max = 0.30 e Å-3 |
| 216 restraints | min = -0.33 e Å-3 |

**Table 4.** *Fractional atomic coordinates and isotropic or equivalent isotropic displacement parameters (Å2) for* ***3g***

|  | *x* | *y* | *z* | *U*iso*/*U*eq | Occ. (<1) |
| --- | --- | --- | --- | --- | --- |
| N1 | 0.3494 (2) | 0.9135 (2) | 0.54498 (6) | 0.0578 (4) |  |
| H1 | 0.391 (3) | 0.930 (3) | 0.5755 (6) | 0.069* |  |
| C2 | 0.3669 (2) | 0.7776 (2) | 0.52547 (7) | 0.0568 (5) |  |
| O2 | 0.4347 (2) | 0.67568 (18) | 0.55225 (5) | 0.0679 (4) |  |
| C3 | 0.3014 (2) | 0.7599 (2) | 0.47258 (7) | 0.0557 (5) |  |
| C4 | 0.2209 (2) | 0.8758 (3) | 0.44555 (7) | 0.0602 (5) |  |
| O4 | 0.1603 (2) | 0.86079 (19) | 0.39674 (5) | 0.0724 (4) |  |
| H4 | 0.179 (4) | 0.774 (2) | 0.3887 (13) | 0.109* |  |
| C4A | 0.1974 (2) | 1.0159 (3) | 0.46847 (7) | 0.0607 (5) |  |
| C5 | 0.1077 (3) | 1.1396 (3) | 0.44366 (9) | 0.0770 (7) |  |
| H5 | 0.0572 | 1.1329 | 0.4094 | 0.092* | 0.264 (4) |
| C20 | 0.0194 (4) | 1.1448 (4) | 0.39167 (12) | 0.0710 (7)* | 0.736 (4) |
| H20A | -0.0349 | 1.2411 | 0.3852 | 0.107* | 0.736 (4) |
| H20B | -0.0690 | 1.0682 | 0.3869 | 0.107* | 0.736 (4) |
| H20C | 0.1031 | 1.1288 | 0.3682 | 0.107* | 0.736 (4) |
| C6 | 0.0964 (3) | 1.2668 (3) | 0.47023 (11) | 0.0826 (7) |  |
| H6 | 0.0377 | 1.3488 | 0.4541 | 0.099* |  |
| C7 | 0.1682 (3) | 1.2780 (3) | 0.51958 (11) | 0.0783 (6) |  |
| H7 | 0.1587 | 1.3681 | 0.5368 | 0.094* | 0.736 (4) |
| C21 | 0.1358 (12) | 1.4105 (8) | 0.5381 (3) | 0.0710 (7)* | 0.264 (4) |
| H21A | 0.0818 | 1.4734 | 0.5108 | 0.107* | 0.264 (4) |
| H21B | 0.2439 | 1.4552 | 0.5536 | 0.107* | 0.264 (4) |
| H21C | 0.0578 | 1.3996 | 0.5636 | 0.107* | 0.264 (4) |
| C8 | 0.2535 (3) | 1.1633 (3) | 0.54498 (9) | 0.0668 (5) |  |
| H8 | 0.3024 | 1.1736 | 0.5792 | 0.080* |  |
| C8A | 0.2669 (2) | 1.0304 (2) | 0.51936 (8) | 0.0581 (5) |  |
| C9 | 0.3241 (2) | 0.6178 (3) | 0.45070 (7) | 0.0576 (5) |  |
| H9 | 0.3785 | 0.5413 | 0.4710 | 0.069* |  |
| N10 | 0.2710 (2) | 0.5939 (2) | 0.40370 (6) | 0.0582 (4) |  |
| N11 | 0.2938 (2) | 0.4520 (2) | 0.38632 (6) | 0.0576 (4) |  |
| H11 | 0.369 (3) | 0.397 (2) | 0.4052 (8) | 0.069* |  |
| S12 | 0.30412 (5) | 0.44438 (6) | 0.32543 (2) | 0.05162 (16) |  |
| O121 | 0.14979 (16) | 0.50549 (19) | 0.29927 (5) | 0.0658 (4) |  |
| O122 | 0.34507 (18) | 0.29427 (16) | 0.31707 (5) | 0.0621 (4) |  |
| C13 | 0.4771 (2) | 0.55682 (19) | 0.31426 (6) | 0.0434 (4) |  |
| C14 | 0.6402 (2) | 0.5286 (2) | 0.33969 (6) | 0.0514 (4) |  |
| H14 | 0.6580 | 0.4509 | 0.3635 | 0.062* |  |
| C15 | 0.7765 (2) | 0.6156 (2) | 0.32981 (7) | 0.0543 (4) |  |
| H15 | 0.8886 | 0.5965 | 0.3469 | 0.065* |  |
| C16 | 0.7527 (2) | 0.7299 (2) | 0.29550 (6) | 0.0504 (4) |  |
| C17 | 0.5877 (2) | 0.7553 (2) | 0.27075 (7) | 0.0541 (4) |  |
| H17 | 0.5693 | 0.8328 | 0.2469 | 0.065* |  |
| C18 | 0.4500 (2) | 0.6699 (2) | 0.28023 (6) | 0.0509 (4) |  |
| H18 | 0.3377 | 0.6893 | 0.2634 | 0.061* |  |
| C19 | 0.9016 (3) | 0.8240 (3) | 0.28479 (9) | 0.0721 (6) |  |
| H19A | 0.9796 | 0.7657 | 0.2671 | 0.108* |  |
| H19B | 0.9639 | 0.8602 | 0.3166 | 0.108* |  |
| H19C | 0.8581 | 0.9074 | 0.2637 | 0.108* |  |
| O1W | 0.5210 (4) | 0.0352 (3) | 0.35231 (8) | 0.1214 (9) |  |
| H1W1 | 0.487 (7) | 0.116 (2) | 0.3390 (14) | 0.182* |  |
| H1W2 | 0.506 (7) | -0.036 (3) | 0.3321 (12) | 0.182* |  |

**Table 5.** *Atomic displacement parameters (Å2) for* ***3g***

|  | *U*11 | *U*22 | *U*33 | *U*12 | *U*13 | *U*23 |
| --- | --- | --- | --- | --- | --- | --- |
| N1 | 0.0469 (8) | 0.0820 (11) | 0.0457 (8) | -0.0042 (7) | 0.0102 (7) | -0.0126 (8) |
| C2 | 0.0421 (9) | 0.0831 (13) | 0.0469 (9) | -0.0058 (8) | 0.0126 (7) | -0.0105 (9) |
| O2 | 0.0678 (9) | 0.0870 (10) | 0.0476 (7) | 0.0074 (7) | 0.0030 (6) | -0.0123 (7) |
| C3 | 0.0393 (8) | 0.0854 (13) | 0.0447 (9) | -0.0130 (8) | 0.0144 (7) | -0.0093 (9) |
| C4 | 0.0435 (9) | 0.0920 (14) | 0.0469 (9) | -0.0212 (9) | 0.0131 (7) | 0.0004 (9) |
| O4 | 0.0749 (10) | 0.0952 (11) | 0.0466 (7) | -0.0164 (9) | 0.0066 (7) | 0.0010 (7) |
| C4A | 0.0450 (9) | 0.0847 (13) | 0.0550 (10) | -0.0197 (9) | 0.0160 (8) | 0.0039 (9) |
| C5 | 0.0608 (12) | 0.1041 (17) | 0.0677 (13) | -0.0246 (12) | 0.0147 (10) | 0.0225 (12) |
| C6 | 0.0712 (14) | 0.0856 (16) | 0.0945 (18) | -0.0094 (12) | 0.0237 (13) | 0.0261 (14) |
| C7 | 0.0696 (14) | 0.0762 (14) | 0.0956 (17) | -0.0050 (11) | 0.0352 (12) | 0.0119 (12) |
| C8 | 0.0553 (11) | 0.0798 (14) | 0.0701 (13) | -0.0076 (10) | 0.0252 (10) | -0.0018 (10) |
| C8A | 0.0402 (9) | 0.0804 (13) | 0.0569 (10) | -0.0113 (8) | 0.0183 (8) | -0.0004 (9) |
| C9 | 0.0410 (9) | 0.0887 (13) | 0.0453 (9) | -0.0127 (9) | 0.0138 (7) | -0.0095 (9) |
| N10 | 0.0435 (8) | 0.0878 (11) | 0.0459 (8) | -0.0167 (7) | 0.0159 (6) | -0.0135 (7) |
| N11 | 0.0496 (9) | 0.0824 (11) | 0.0425 (8) | -0.0161 (7) | 0.0118 (6) | -0.0082 (7) |
| S12 | 0.0370 (2) | 0.0780 (3) | 0.0392 (2) | -0.00889 (18) | 0.00299 (16) | -0.01083 (18) |
| O121 | 0.0356 (6) | 0.1025 (11) | 0.0569 (8) | -0.0057 (7) | -0.0025 (5) | -0.0134 (7) |
| O122 | 0.0610 (8) | 0.0750 (9) | 0.0492 (7) | -0.0138 (6) | 0.0034 (6) | -0.0113 (6) |
| C13 | 0.0342 (7) | 0.0647 (10) | 0.0315 (7) | 0.0020 (6) | 0.0047 (6) | -0.0067 (6) |
| C14 | 0.0378 (8) | 0.0739 (12) | 0.0420 (8) | 0.0028 (7) | 0.0033 (7) | 0.0123 (8) |
| C15 | 0.0323 (8) | 0.0836 (13) | 0.0459 (9) | 0.0021 (8) | 0.0017 (6) | 0.0121 (8) |
| C16 | 0.0449 (9) | 0.0695 (11) | 0.0379 (8) | 0.0006 (8) | 0.0092 (6) | 0.0030 (7) |
| C17 | 0.0533 (10) | 0.0652 (11) | 0.0427 (9) | 0.0078 (8) | 0.0031 (7) | 0.0079 (8) |
| C18 | 0.0392 (8) | 0.0710 (11) | 0.0405 (8) | 0.0098 (7) | -0.0022 (6) | -0.0011 (7) |
| C19 | 0.0579 (11) | 0.0983 (16) | 0.0603 (12) | -0.0134 (11) | 0.0093 (9) | 0.0194 (11) |
| O1W | 0.167 (2) | 0.1033 (15) | 0.0799 (13) | 0.0425 (15) | -0.0324 (14) | -0.0403 (11) |

**Table 6.** *Geometric parameters (Å, º) for* ***3g***

| N1—C2 | 1.358 (3) | C8—C8A | 1.404 (3) |
| --- | --- | --- | --- |
| N1—C8A | 1.380 (3) | C8—H8 | 0.9500 |
| N1—H1 | 0.850 (16) | C9—N10 | 1.290 (2) |
| C2—O2 | 1.248 (3) | C9—H9 | 0.9500 |
| C2—C3 | 1.449 (3) | N10—N11 | 1.394 (2) |
| C3—C4 | 1.384 (3) | N11—S12 | 1.6455 (15) |
| C3—C9 | 1.443 (3) | N11—H11 | 0.882 (16) |
| C4—O4 | 1.336 (2) | S12—O121 | 1.4263 (14) |
| C4—C4A | 1.440 (3) | S12—O122 | 1.4301 (16) |
| O4—H4 | 0.836 (18) | S12—C13 | 1.7574 (17) |
| C4A—C8A | 1.404 (3) | C13—C18 | 1.374 (3) |
| C4A—C5 | 1.443 (3) | C13—C14 | 1.389 (2) |
| C5—C6 | 1.370 (4) | C14—C15 | 1.385 (3) |
| C5—C20 | 1.469 (4) | C14—H14 | 0.9500 |
| C5—H5 | 0.9500 | C15—C16 | 1.385 (3) |
| C20—H20A | 0.9800 | C15—H15 | 0.9500 |
| C20—H20B | 0.9800 | C16—C17 | 1.390 (2) |
| C20—H20C | 0.9800 | C16—C19 | 1.507 (3) |
| C6—C7 | 1.369 (4) | C17—C18 | 1.382 (3) |
| C6—H6 | 0.9500 | C17—H17 | 0.9500 |
| C7—C21 | 1.344 (7) | C18—H18 | 0.9500 |
| C7—C8 | 1.371 (3) | C19—H19A | 0.9800 |
| C7—H7 | 0.9500 | C19—H19B | 0.9800 |
| C21—H21A | 0.9800 | C19—H19C | 0.9800 |
| C21—H21B | 0.9800 | O1W—H1W1 | 0.842 (5) |
| C21—H21C | 0.9800 | O1W—H1W2 | 0.840 (5) |
|  |  |  |  |
| C2—N1—C8A | 125.30 (17) | C8A—C8—H8 | 120.7 |
| C2—N1—H1 | 118.9 (16) | N1—C8A—C8 | 119.01 (19) |
| C8A—N1—H1 | 115.8 (16) | N1—C8A—C4A | 120.28 (19) |
| O2—C2—N1 | 121.00 (17) | C8—C8A—C4A | 120.7 (2) |
| O2—C2—C3 | 123.07 (19) | N10—C9—C3 | 120.5 (2) |
| N1—C2—C3 | 115.92 (19) | N10—C9—H9 | 119.8 |
| C4—C3—C9 | 123.06 (17) | C3—C9—H9 | 119.8 |
| C4—C3—C2 | 120.14 (19) | C9—N10—N11 | 116.34 (19) |
| C9—C3—C2 | 116.80 (19) | N10—N11—S12 | 113.34 (13) |
| O4—C4—C3 | 120.7 (2) | N10—N11—H11 | 116.4 (16) |
| O4—C4—C4A | 117.3 (2) | S12—N11—H11 | 115.2 (16) |
| C3—C4—C4A | 122.00 (18) | O121—S12—O122 | 119.36 (9) |
| C4—O4—H4 | 107 (2) | O121—S12—N11 | 108.53 (9) |
| C8A—C4A—C4 | 116.2 (2) | O122—S12—N11 | 103.76 (9) |
| C8A—C4A—C5 | 118.3 (2) | O121—S12—C13 | 108.13 (9) |
| C4—C4A—C5 | 125.4 (2) | O122—S12—C13 | 109.68 (8) |
| C6—C5—C4A | 119.0 (2) | N11—S12—C13 | 106.66 (8) |
| C6—C5—C20 | 114.2 (3) | C18—C13—C14 | 120.92 (16) |
| C4A—C5—C20 | 126.8 (3) | C18—C13—S12 | 119.82 (12) |
| C6—C5—H5 | 120.5 | C14—C13—S12 | 119.26 (13) |
| C4A—C5—H5 | 120.5 | C15—C14—C13 | 118.87 (16) |
| C5—C20—H20A | 109.5 | C15—C14—H14 | 120.6 |
| C5—C20—H20B | 109.5 | C13—C14—H14 | 120.6 |
| H20A—C20—H20B | 109.5 | C14—C15—C16 | 121.33 (16) |
| C5—C20—H20C | 109.5 | C14—C15—H15 | 119.3 |
| H20A—C20—H20C | 109.5 | C16—C15—H15 | 119.3 |
| H20B—C20—H20C | 109.5 | C15—C16—C17 | 118.32 (17) |
| C7—C6—C5 | 121.2 (3) | C15—C16—C19 | 121.10 (17) |
| C7—C6—H6 | 119.4 | C17—C16—C19 | 120.58 (17) |
| C5—C6—H6 | 119.4 | C18—C17—C16 | 121.19 (17) |
| C21—C7—C6 | 110.5 (5) | C18—C17—H17 | 119.4 |
| C21—C7—C8 | 127.3 (5) | C16—C17—H17 | 119.4 |
| C6—C7—C8 | 122.1 (3) | C13—C18—C17 | 119.37 (15) |
| C6—C7—H7 | 118.9 | C13—C18—H18 | 120.3 |
| C8—C7—H7 | 118.9 | C17—C18—H18 | 120.3 |
| C7—C21—H21A | 109.5 | C16—C19—H19A | 109.5 |
| C7—C21—H21B | 109.5 | C16—C19—H19B | 109.5 |
| H21A—C21—H21B | 109.5 | H19A—C19—H19B | 109.5 |
| C7—C21—H21C | 109.5 | C16—C19—H19C | 109.5 |
| H21A—C21—H21C | 109.5 | H19A—C19—H19C | 109.5 |
| H21B—C21—H21C | 109.5 | H19B—C19—H19C | 109.5 |
| C7—C8—C8A | 118.7 (2) | H1W1—O1W—H1W2 | 112.8 (14) |
| C7—C8—H8 | 120.7 |  |  |
|  |  |  |  |
| C8A—N1—C2—O2 | -175.71 (17) | C4—C4A—C8A—N1 | -1.2 (2) |
| C8A—N1—C2—C3 | 3.8 (3) | C5—C4A—C8A—N1 | 178.10 (16) |
| O2—C2—C3—C4 | 177.18 (17) | C4—C4A—C8A—C8 | 178.97 (16) |
| N1—C2—C3—C4 | -2.3 (2) | C5—C4A—C8A—C8 | -1.8 (3) |
| O2—C2—C3—C9 | -2.6 (3) | C4—C3—C9—N10 | 1.1 (3) |
| N1—C2—C3—C9 | 177.93 (15) | C2—C3—C9—N10 | -179.21 (16) |
| C9—C3—C4—O4 | -0.4 (3) | C3—C9—N10—N11 | -178.10 (14) |
| C2—C3—C4—O4 | 179.85 (16) | C9—N10—N11—S12 | -155.07 (13) |
| C9—C3—C4—C4A | 179.00 (15) | N10—N11—S12—O121 | -57.88 (14) |
| C2—C3—C4—C4A | -0.7 (3) | N10—N11—S12—O122 | 174.21 (12) |
| O4—C4—C4A—C8A | -178.08 (16) | N10—N11—S12—C13 | 58.41 (14) |
| C3—C4—C4A—C8A | 2.5 (2) | O121—S12—C13—C18 | -7.00 (16) |
| O4—C4—C4A—C5 | 2.7 (3) | O122—S12—C13—C18 | 124.68 (14) |
| C3—C4—C4A—C5 | -176.75 (17) | N11—S12—C13—C18 | -123.56 (15) |
| C8A—C4A—C5—C6 | 1.2 (3) | O121—S12—C13—C14 | 173.76 (14) |
| C4—C4A—C5—C6 | -179.59 (18) | O122—S12—C13—C14 | -54.56 (16) |
| C8A—C4A—C5—C20 | -177.0 (2) | N11—S12—C13—C14 | 57.20 (16) |
| C4—C4A—C5—C20 | 2.2 (3) | C18—C13—C14—C15 | -0.7 (3) |
| C4A—C5—C6—C7 | -0.1 (3) | S12—C13—C14—C15 | 178.52 (14) |
| C20—C5—C6—C7 | 178.3 (2) | C13—C14—C15—C16 | 0.4 (3) |
| C5—C6—C7—C21 | -177.9 (5) | C14—C15—C16—C17 | -0.3 (3) |
| C5—C6—C7—C8 | -0.5 (3) | C14—C15—C16—C19 | -179.8 (2) |
| C21—C7—C8—C8A | 176.9 (5) | C15—C16—C17—C18 | 0.5 (3) |
| C6—C7—C8—C8A | -0.1 (3) | C19—C16—C17—C18 | -179.96 (19) |
| C2—N1—C8A—C8 | 177.77 (17) | C14—C13—C18—C17 | 0.9 (3) |
| C2—N1—C8A—C4A | -2.1 (3) | S12—C13—C18—C17 | -178.33 (14) |
| C7—C8—C8A—N1 | -178.67 (18) | C16—C17—C18—C13 | -0.8 (3) |
| C7—C8—C8A—C4A | 1.2 (3) |  |  |

**Table 7.** *Hydrogen-bond geometry (Å, º) for* ***3g***

| *D*—H···*A* | *D*—H | H···*A* | *D*···*A* | *D*—H···*A* |
| --- | --- | --- | --- | --- |
| N1—H1···O1*W*i | 0.85 (2) | 1.99 (2) | 2.839 (3) | 178 (2) |
| O4—H4···N10 | 0.84 (2) | 1.82 (2) | 2.582 (3) | 151 (3) |
| C9—H9···O2i | 0.95 | 2.59 | 3.283 (3) | 130 |
| N11—H11···O2i | 0.88 (2) | 1.90 (2) | 2.764 (2) | 165 (2) |
| C18—H18···O122ii | 0.95 | 2.60 | 3.434 (2) | 146 |
| O1*W*—H1*W*1···O122 | 0.84 (1) | 2.01 (2) | 2.831 (3) | 164 (5) |

Symmetry codes: (i) -*x*+1, -*y*+1, -*z*+1; (ii) -*x*+1/2, *y*+1/2, -*z*+1/2.

**Spectral data**


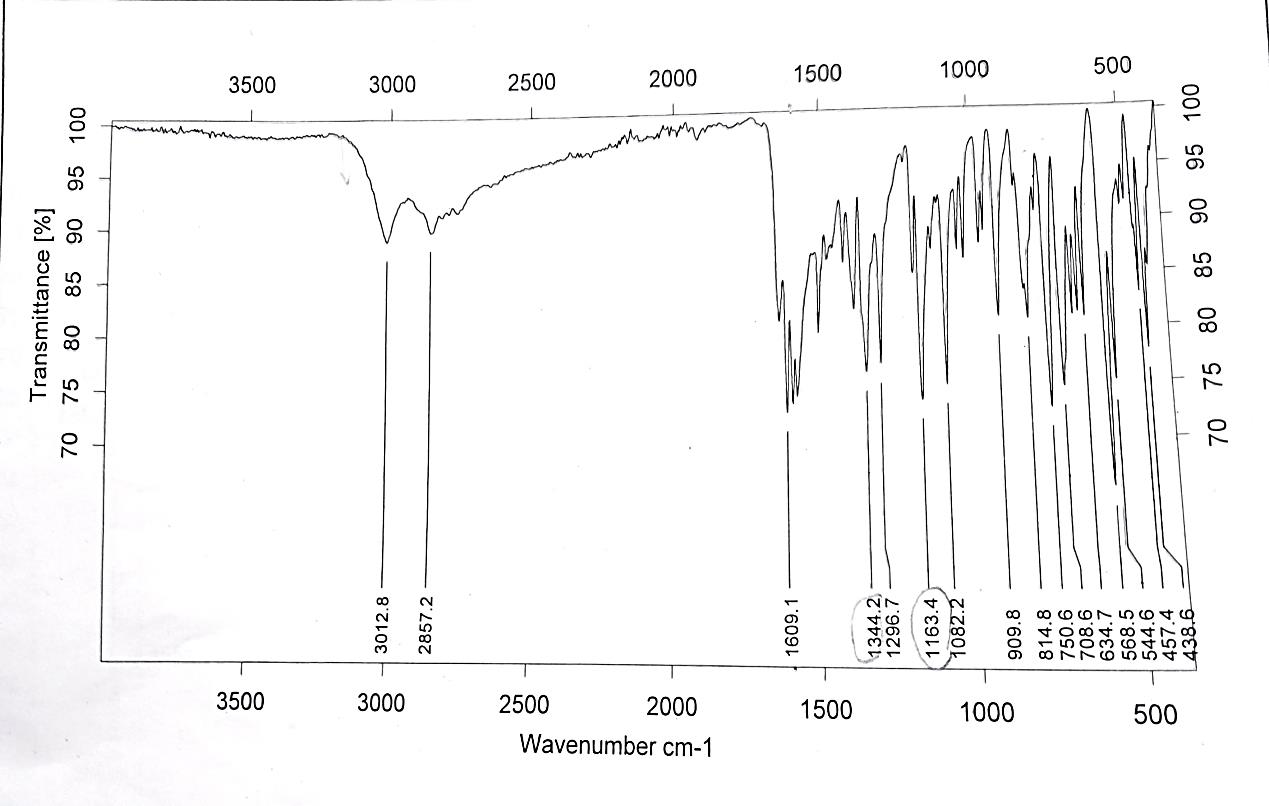


**S1. IR for compound 3a**

**
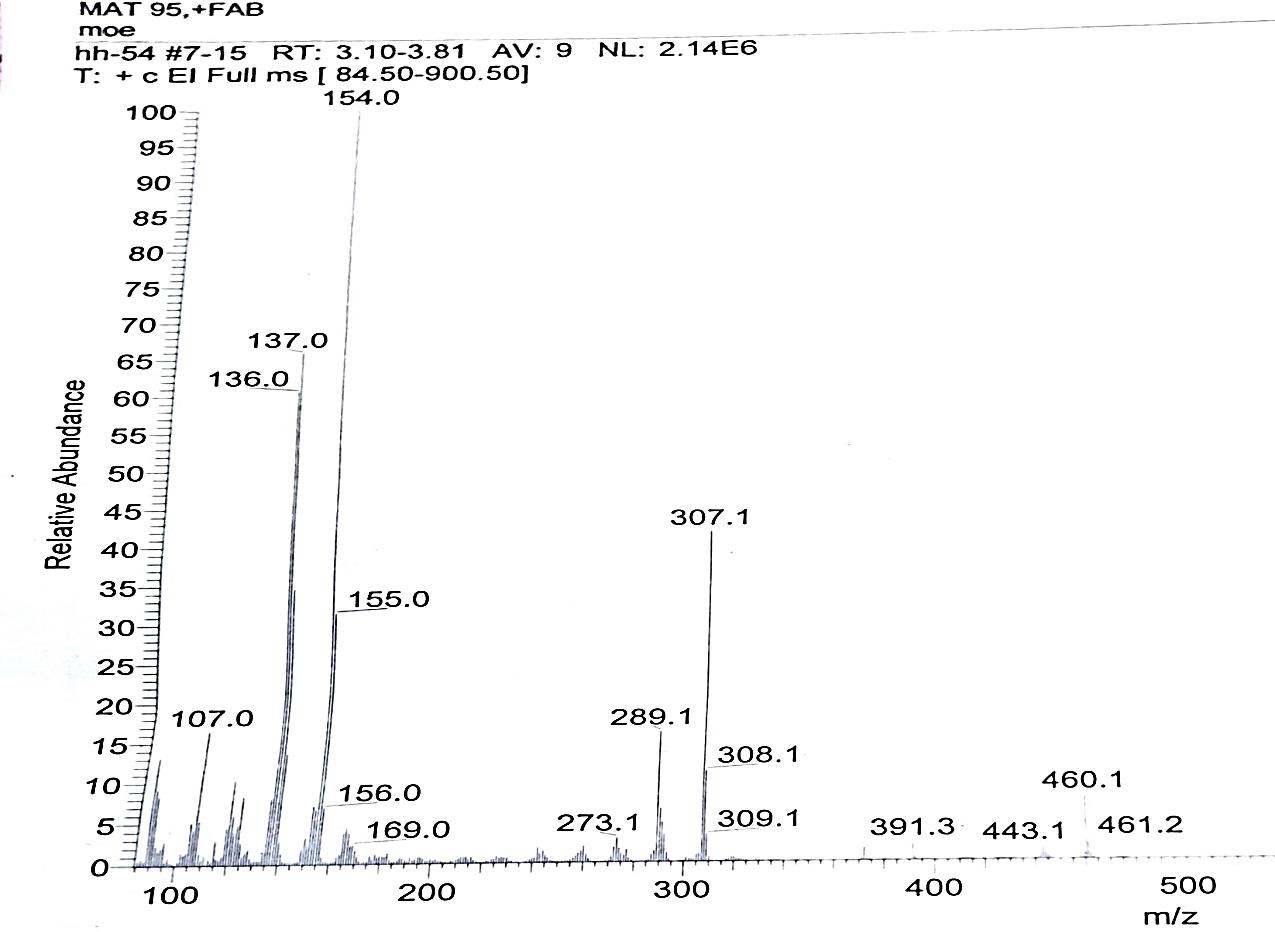
**

**S2. Mass for compound 3a**


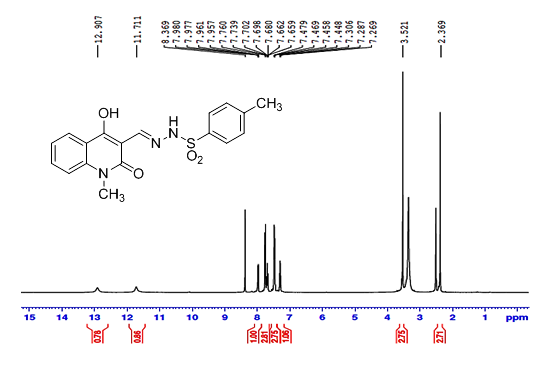


**S3. 1H NMR for compound 3a**

**
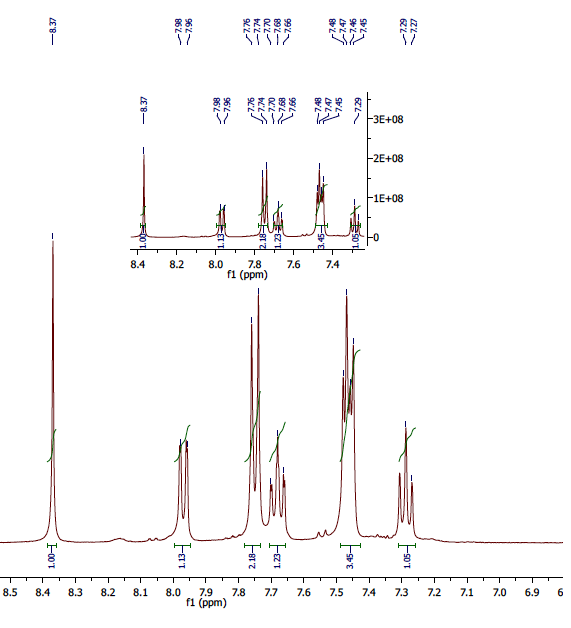
**

**S4.** Expanded 1H NMR spectrum of compound **3a**


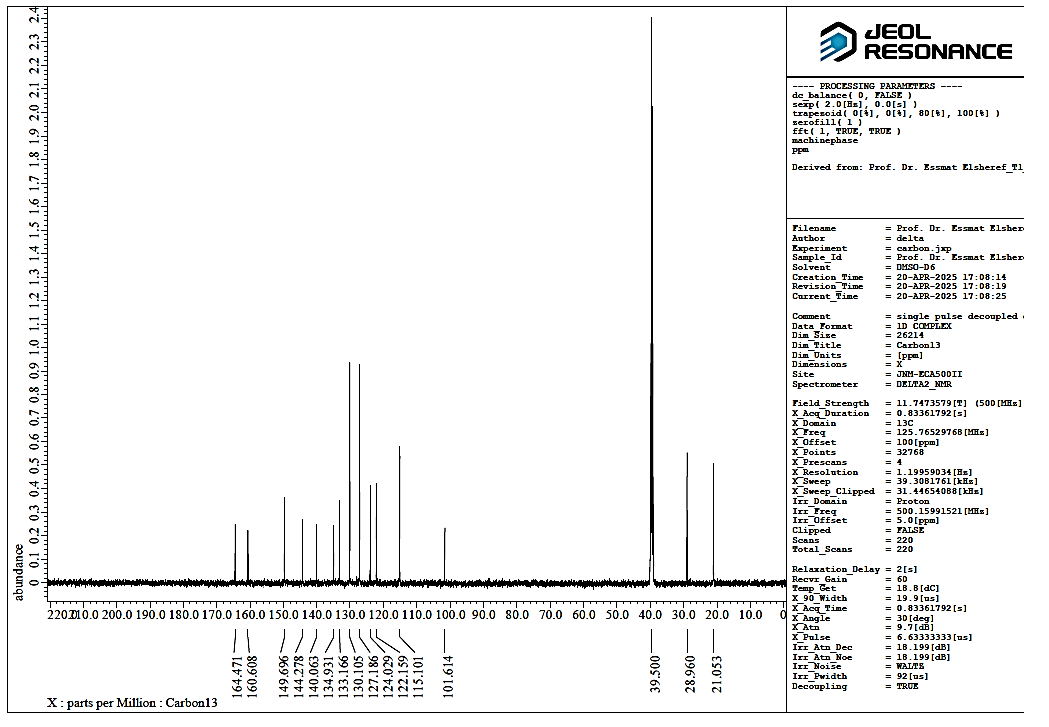


**S5. 13C NMR spectrum for compound 3a**


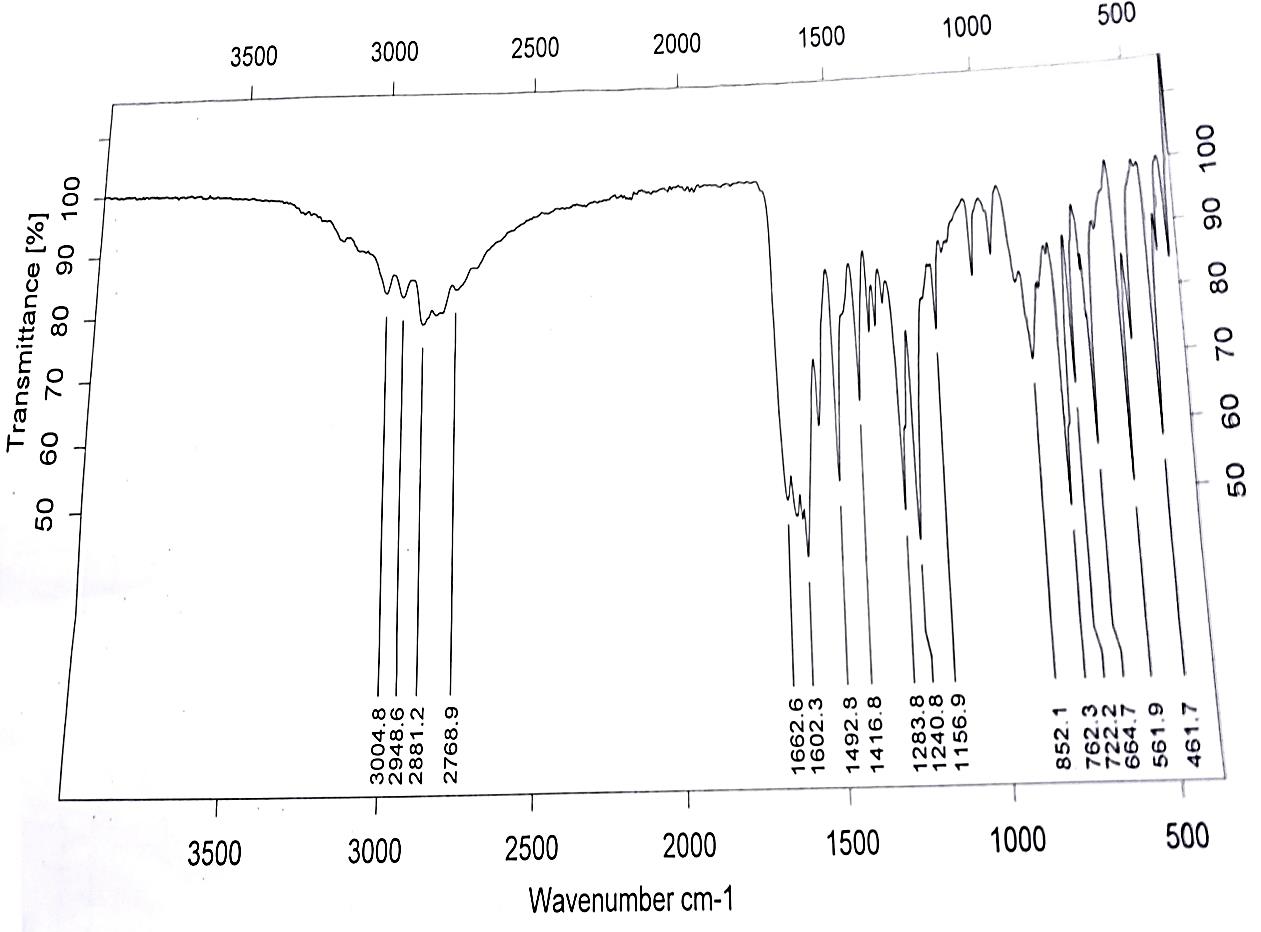


**S6. IR spectrum for compound 3b**

**
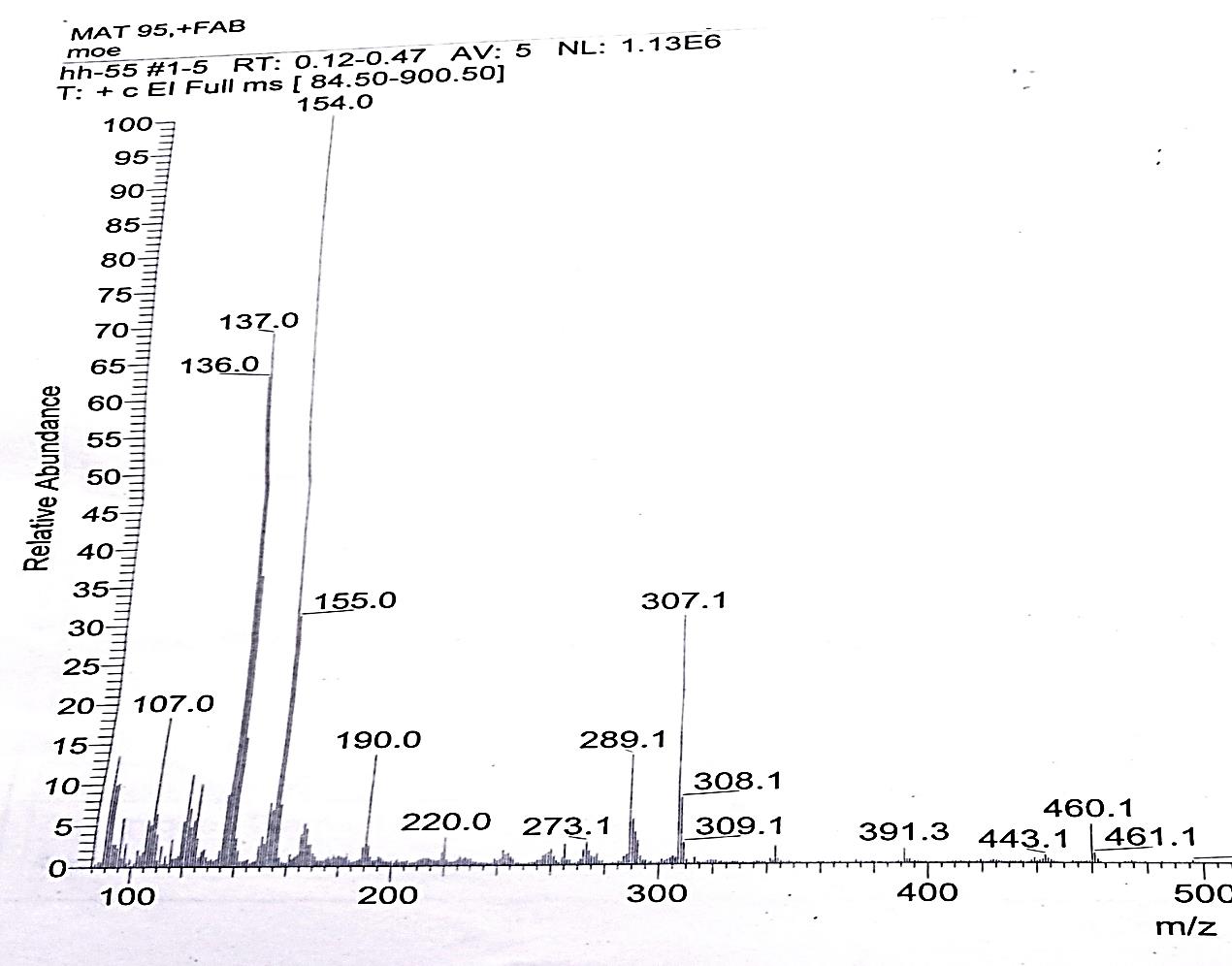
**

**S7. Mass spectrum for compound 3b**


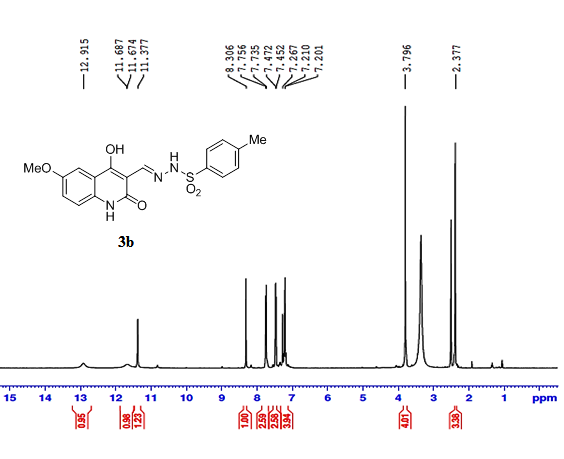


**S8. 1H NMR for compound 3b**

**
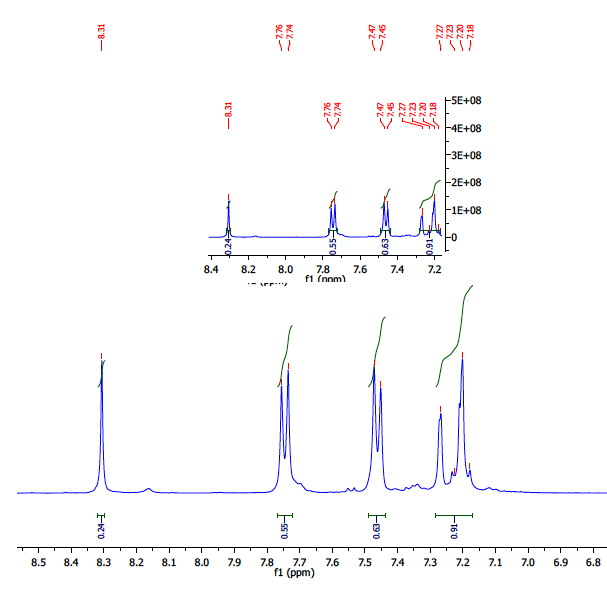
**

**S9**. Expanded 1H NMR spectrum of compound **3b**


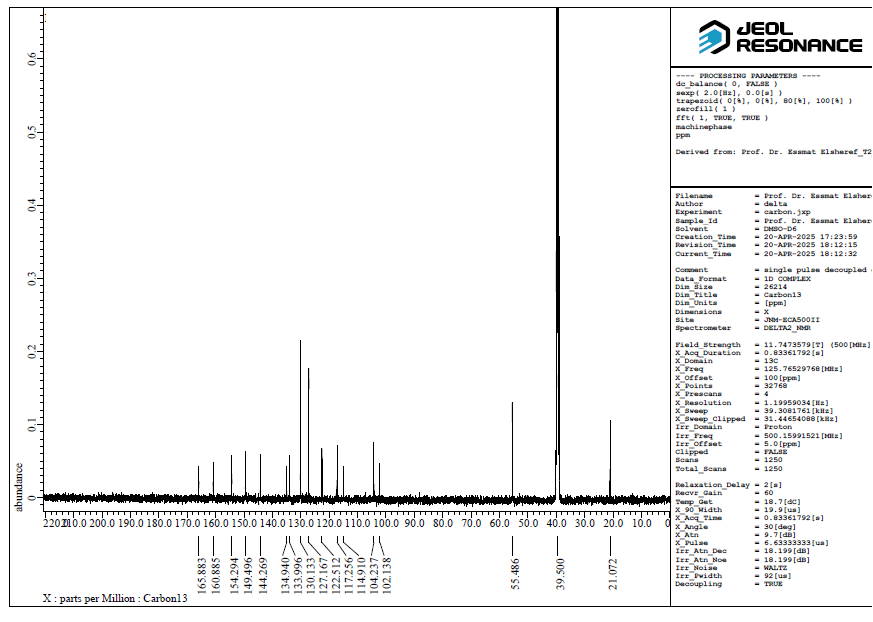


**S10**. 13C NMR of compound **3b**


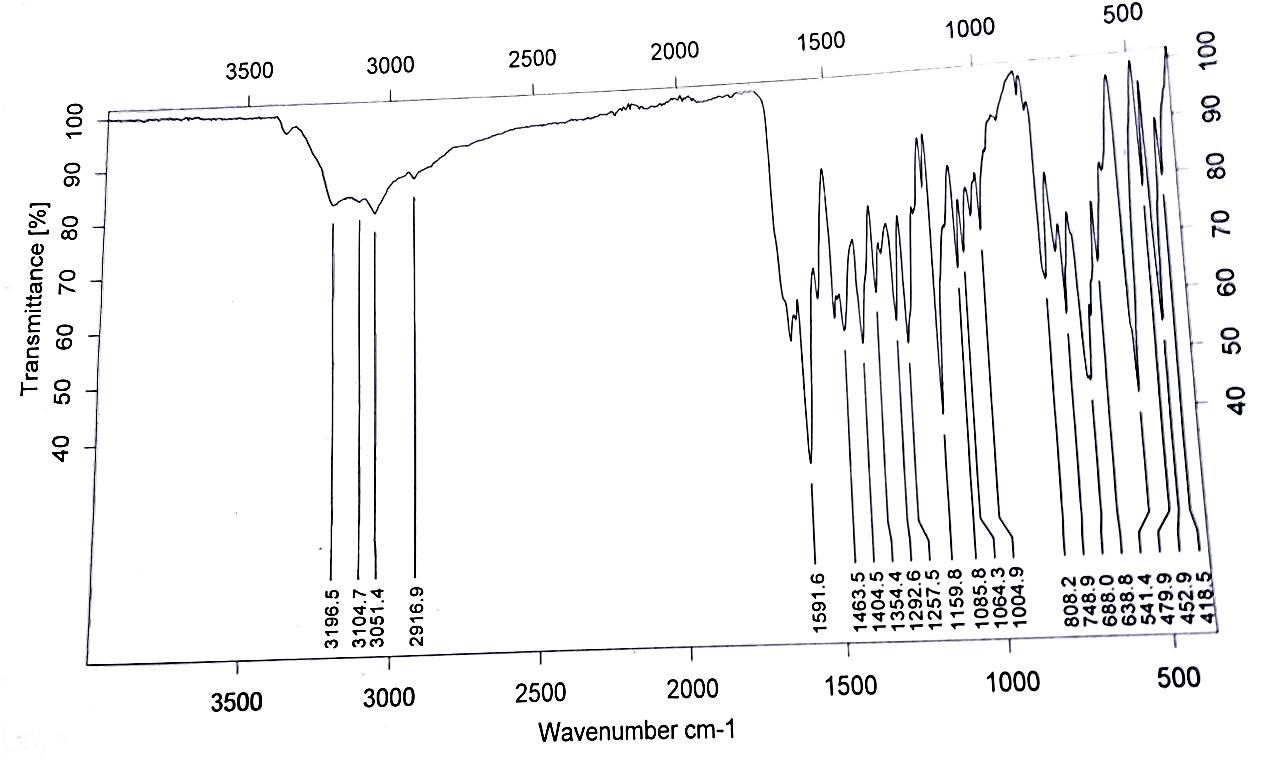


**S11. IR spectrum for compound 3c**

**
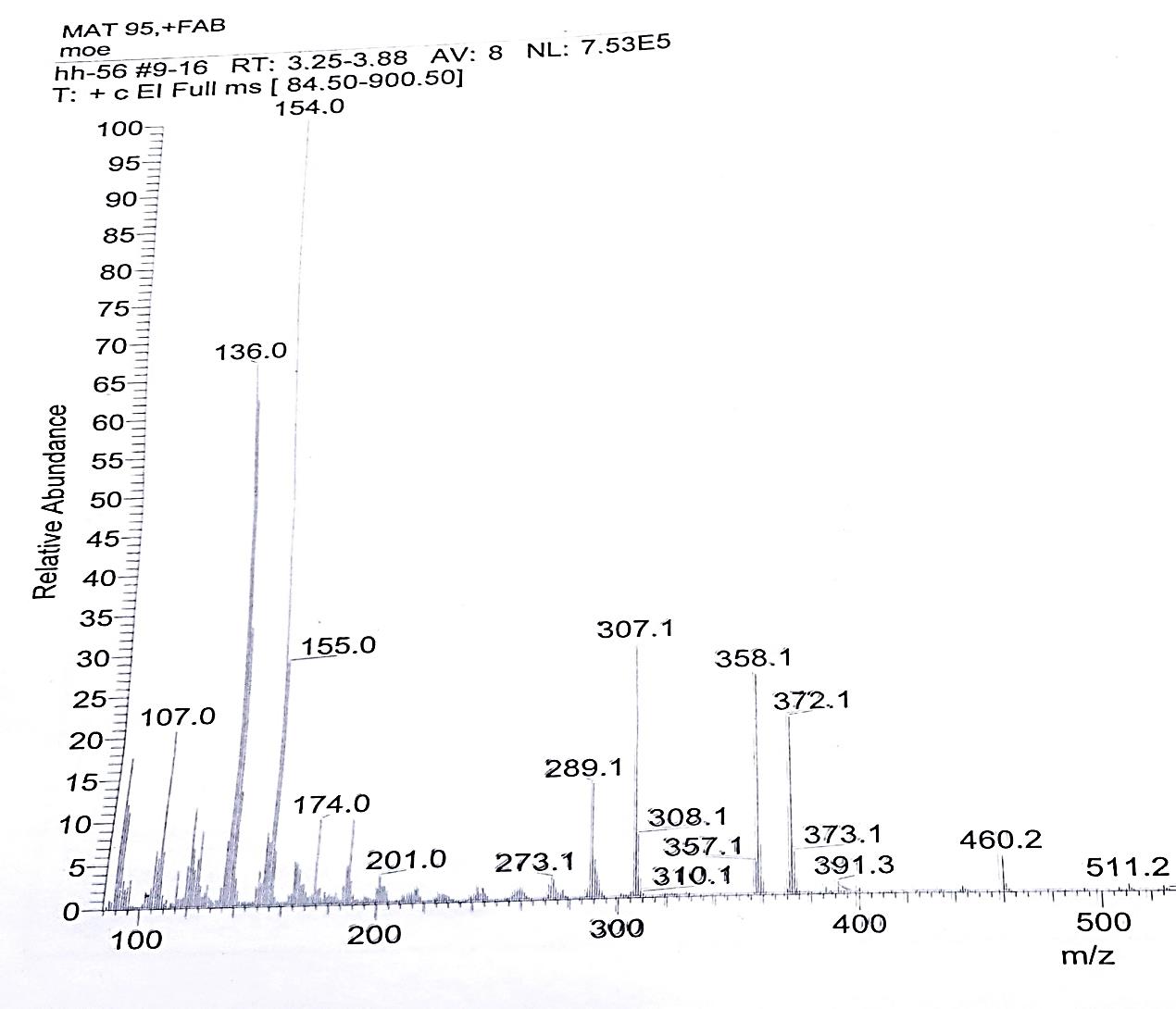
**

**S12. Mass spectrum for compound 3c**


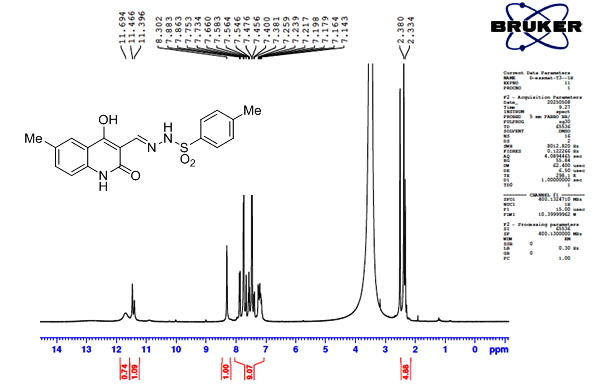


**S13.** 1H NMR Compound **3c**


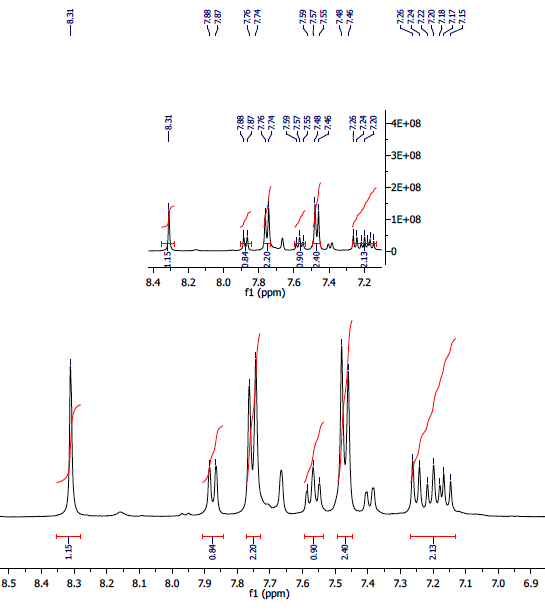


**S14**. Expanded 1H NMR spectrum of compound **3c**


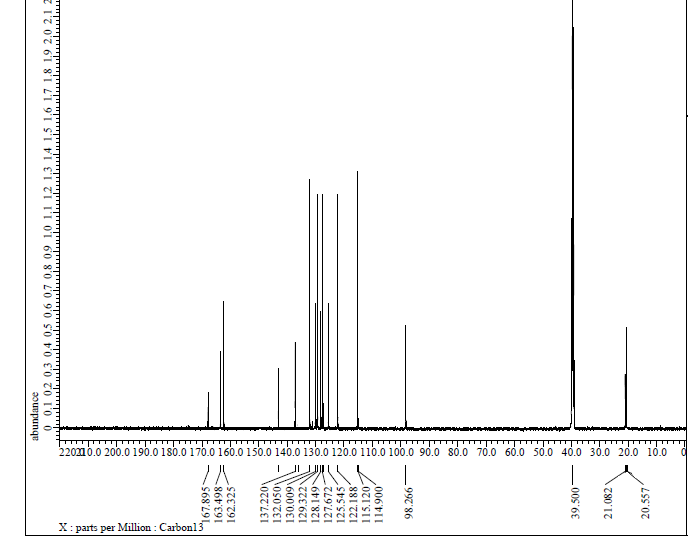


**S15**. 13C NMR of compound **3c**


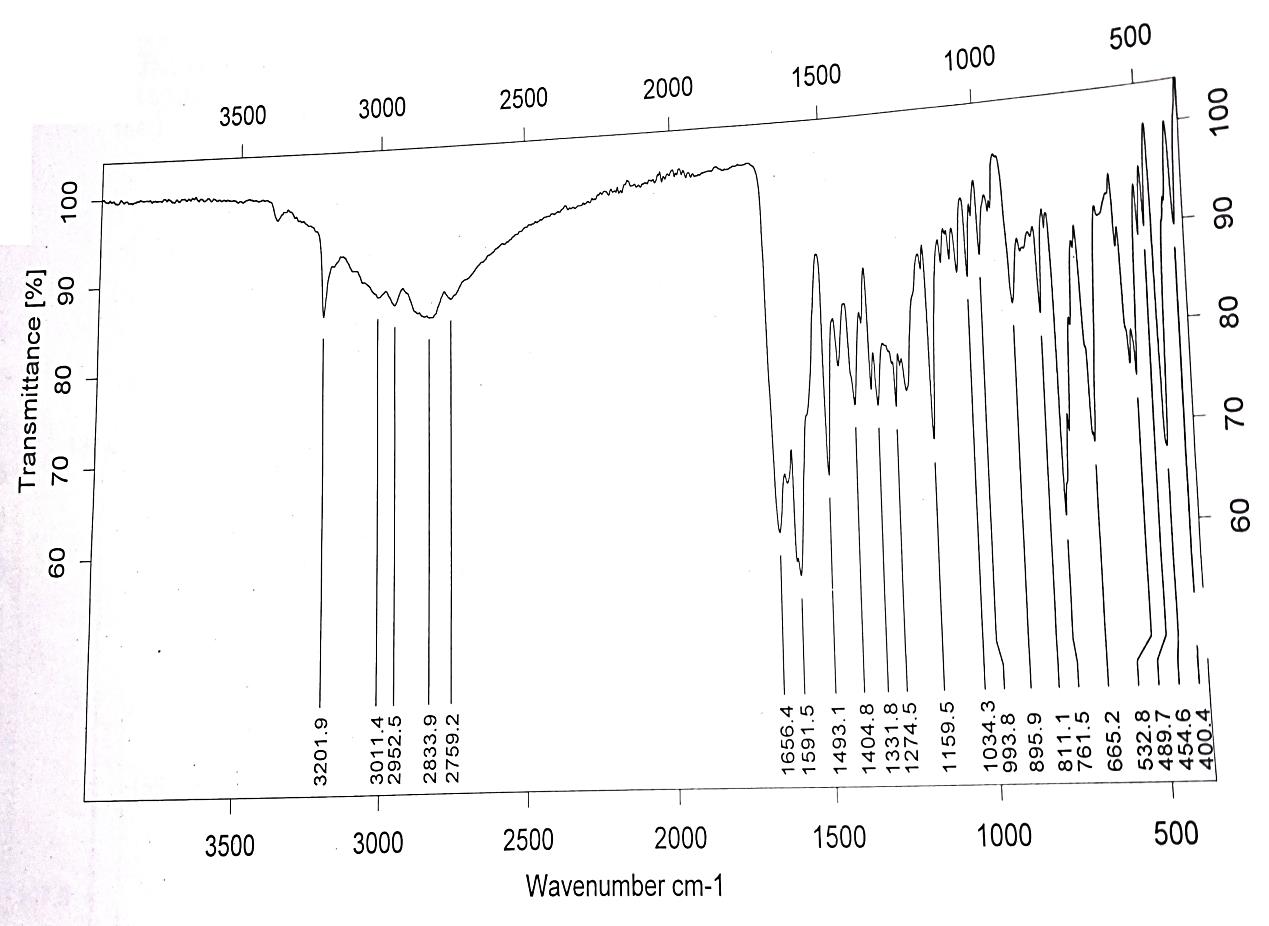


**S16. IR spectrum for compound 3d**


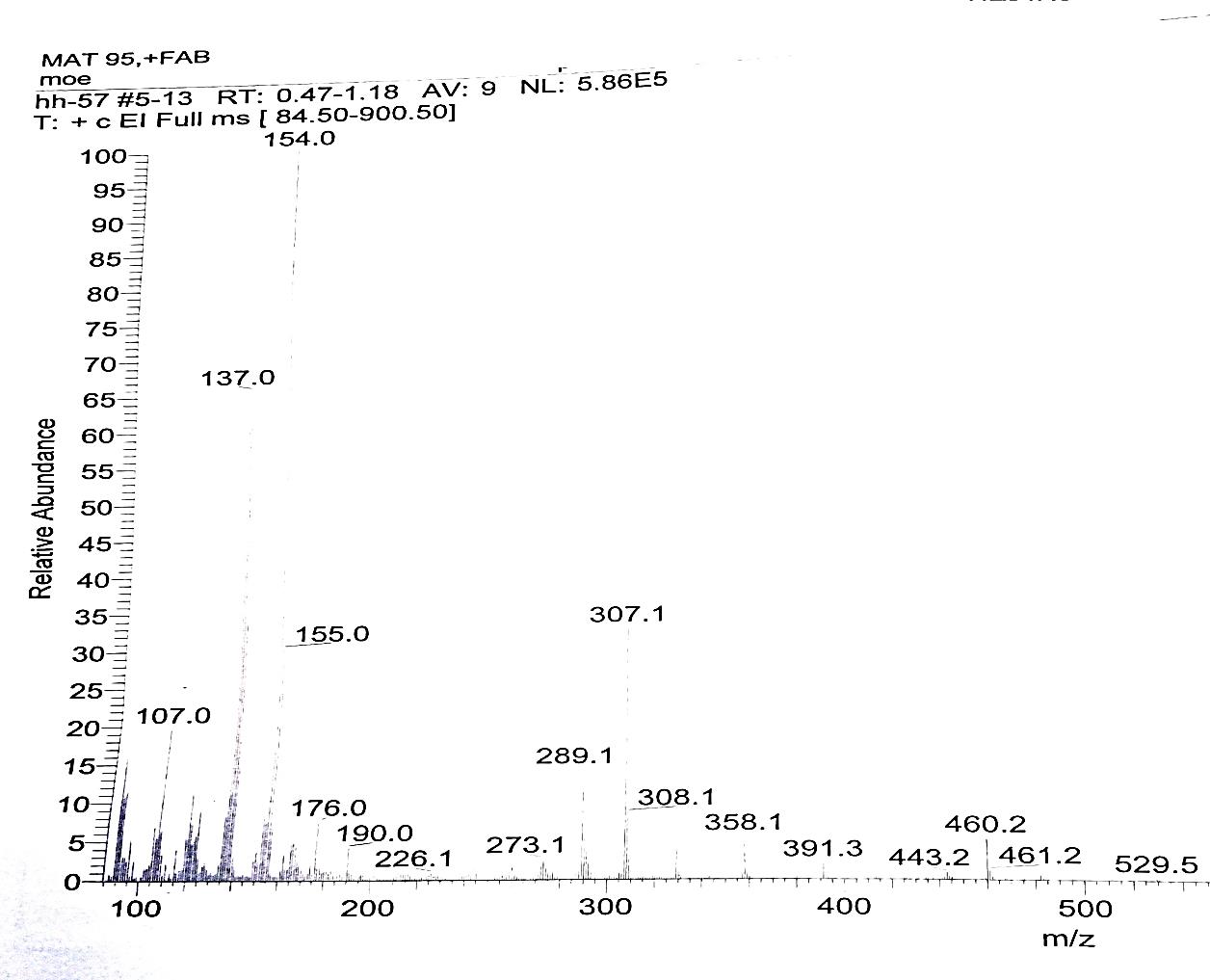


**S17. Mass spectrum for compound 3d**


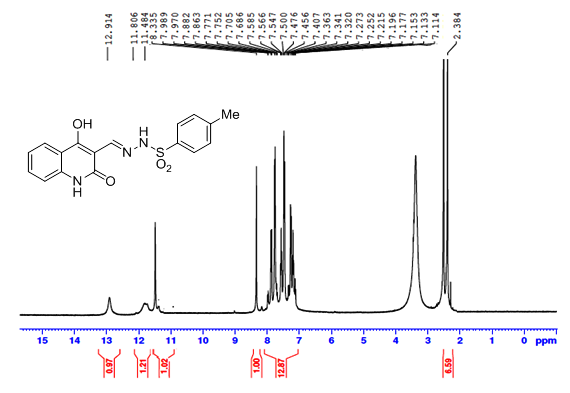


**S18.** 1H NMR Compound **3d**

**S19**. Expanded 1H NMR spectrum of compound **3d**
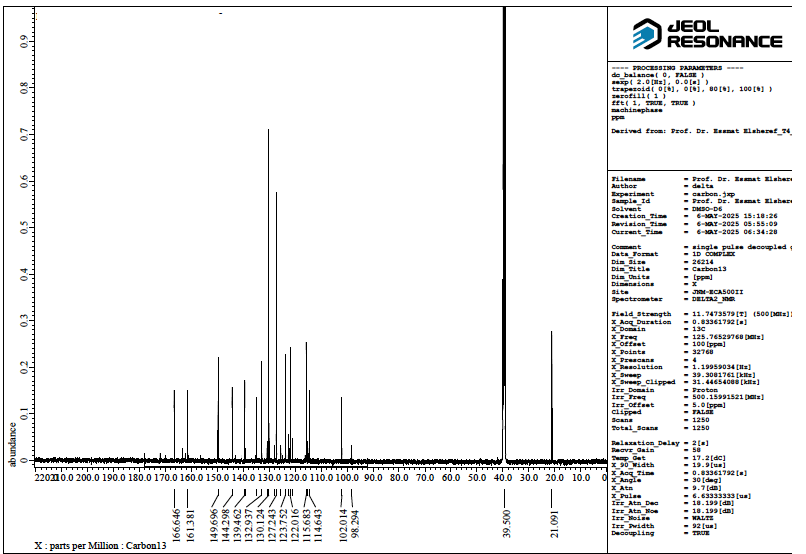


**S20.** 13C NMR Compound **3d**


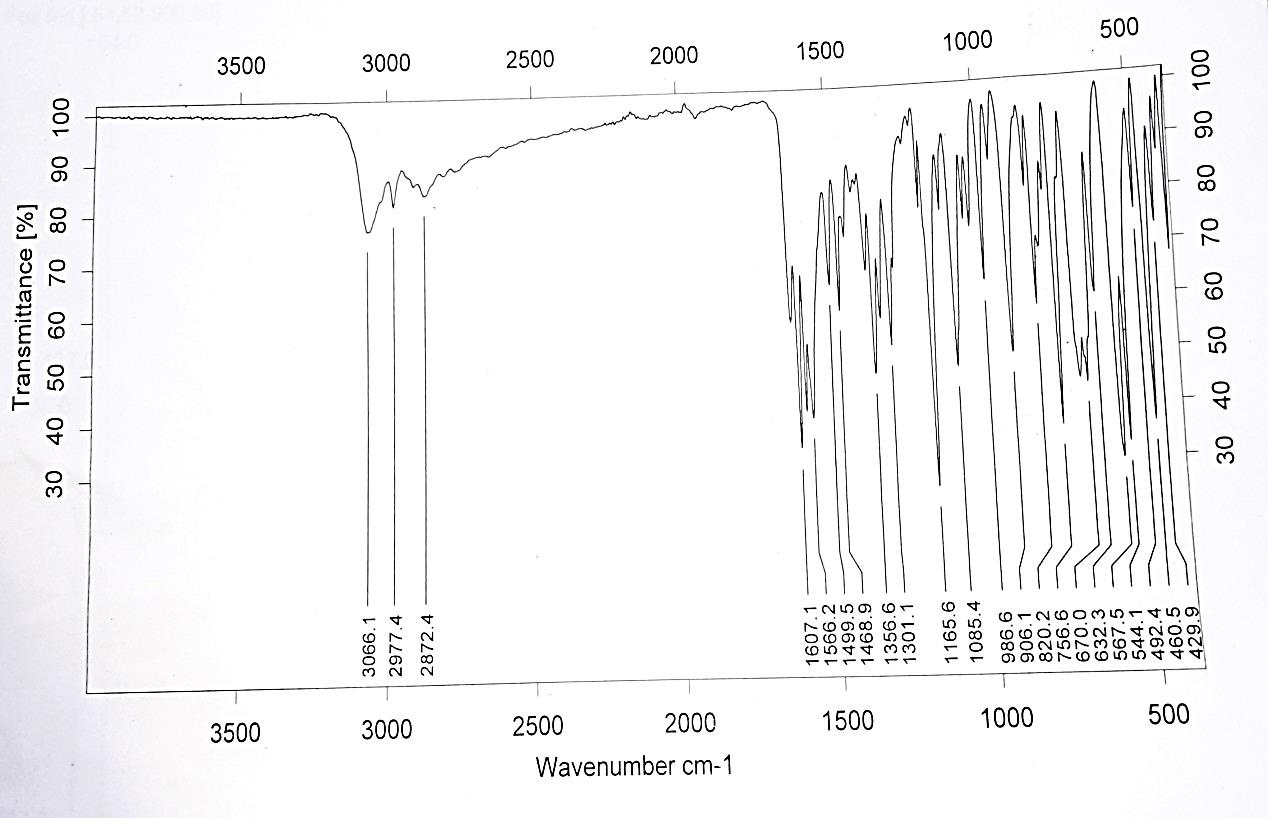


**S21. IR spectrum for compound 3e**

**
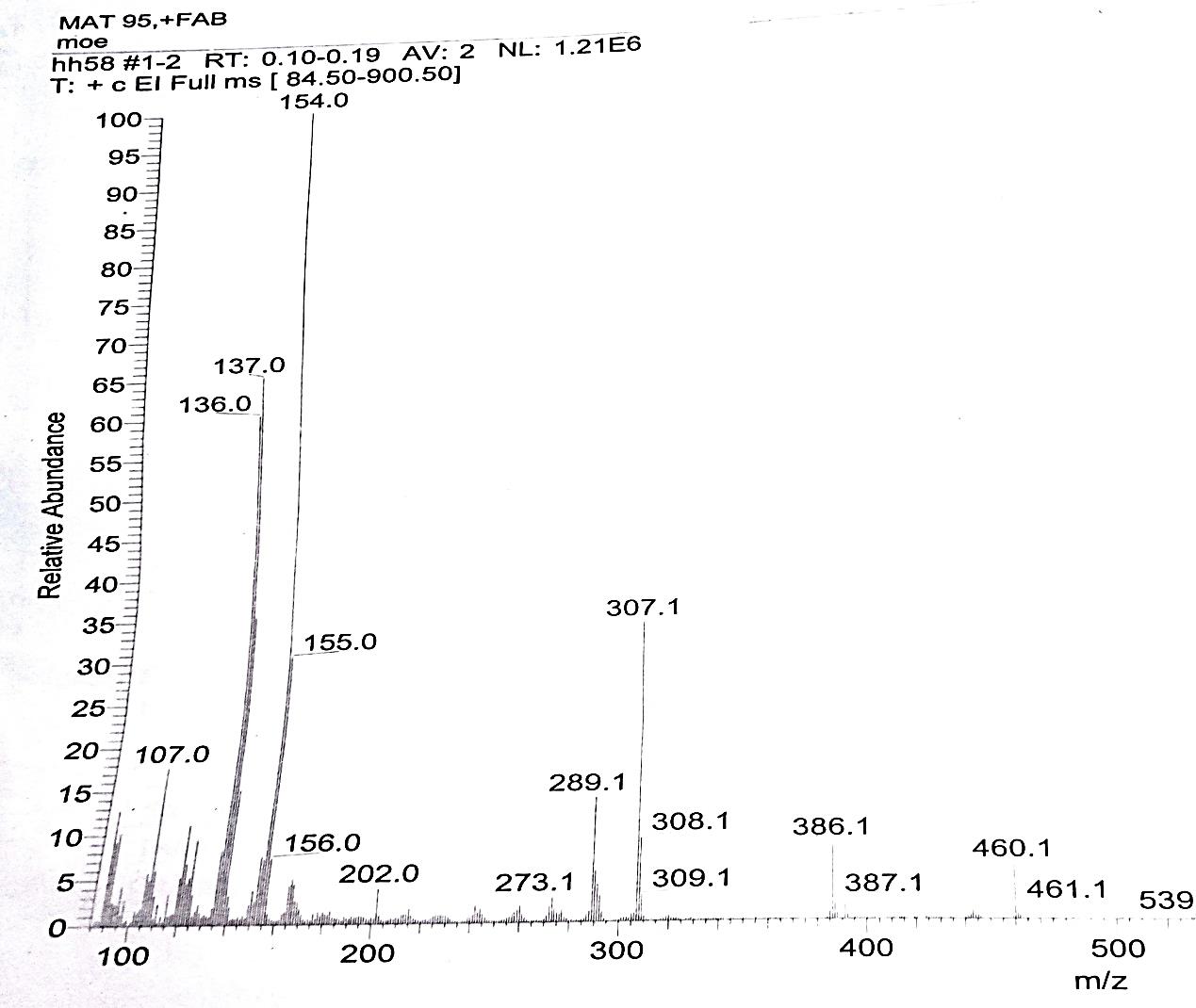
**

**S22. Mass spectrum for compound 3e**


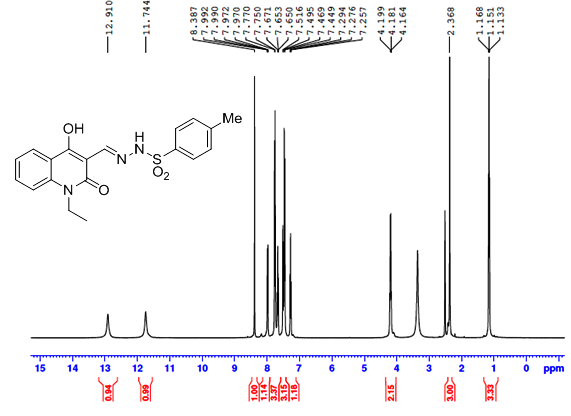


**S23.** 1H NMR Compound **3e**

**
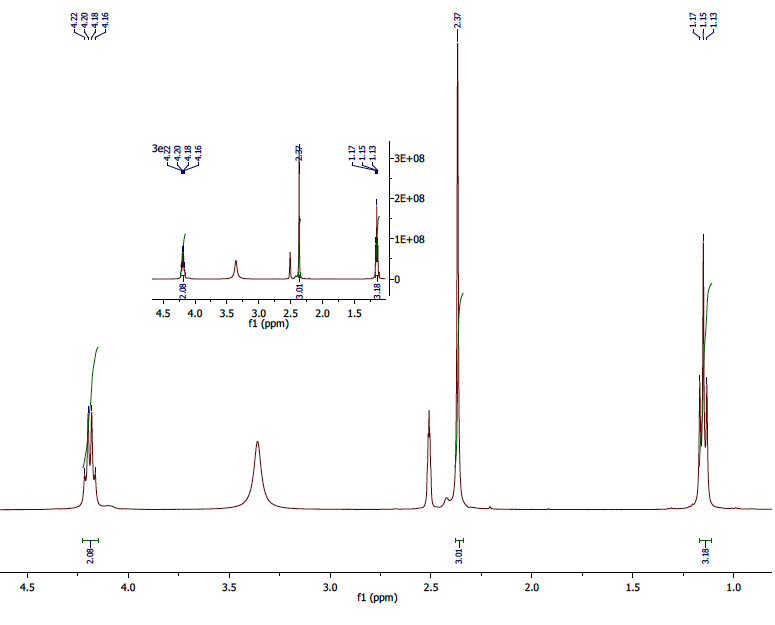
**

**S24**. Expanded 1H NMR spectrum of compound **3**

**
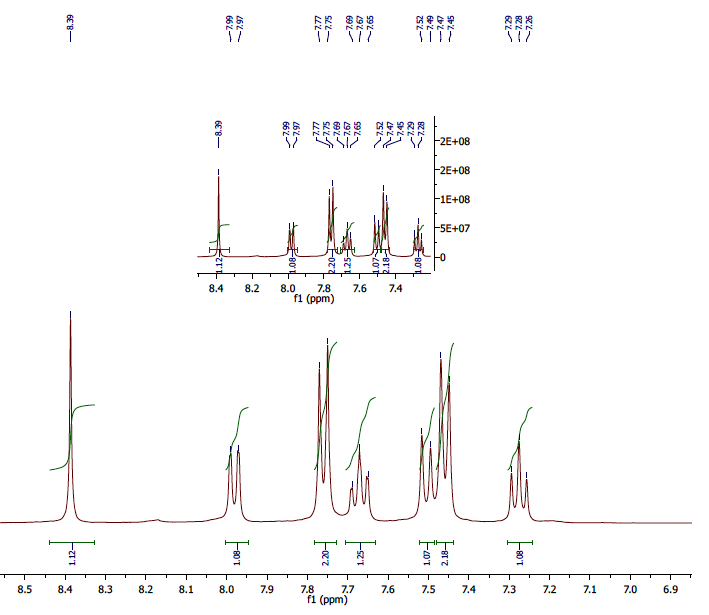
**

**S25**. Expanded 1H NMR spectrum of compound **3e**

**
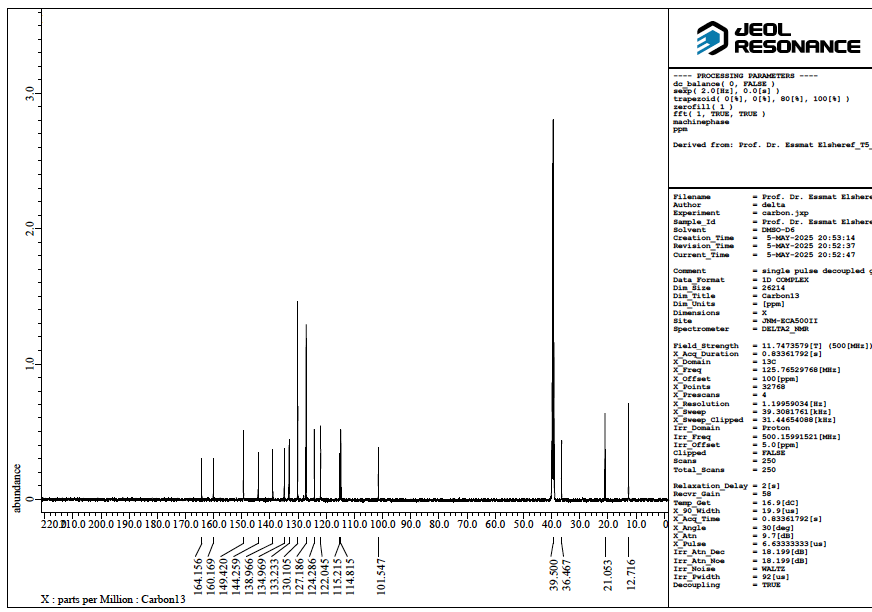
**

**S26.** 13C NMR Compound **3e**

**
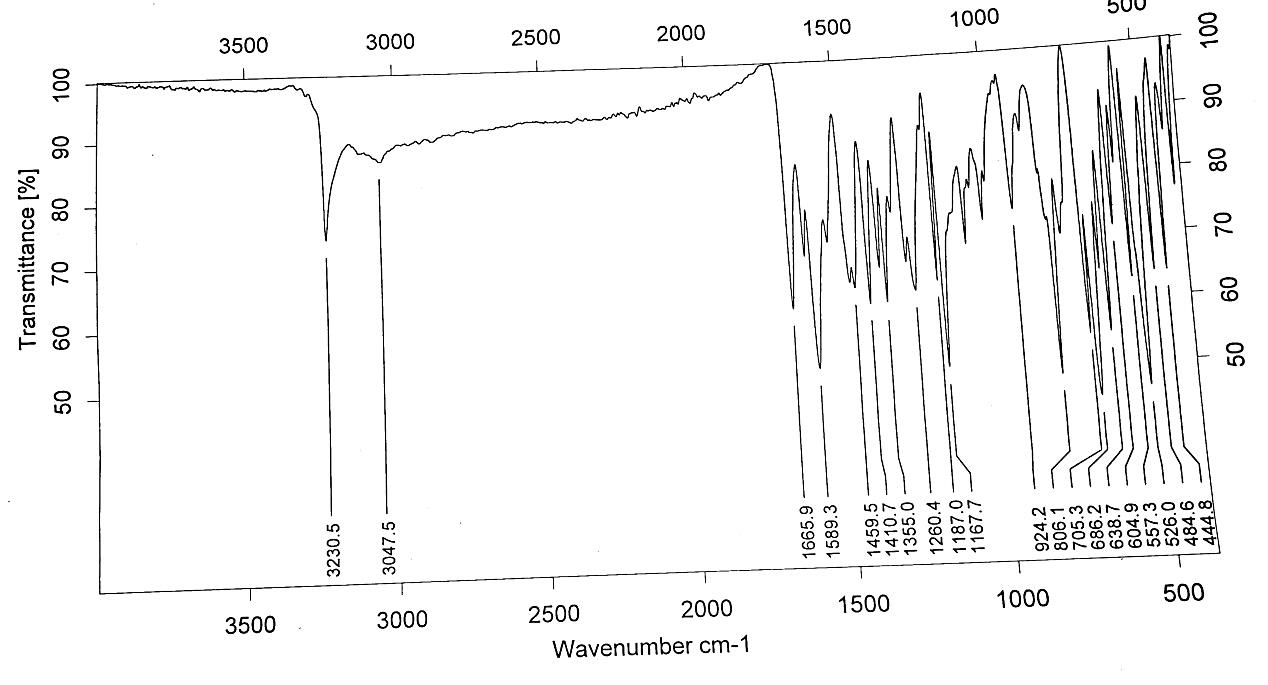
**

**S27. Mass spectrum for compound 3f**

**
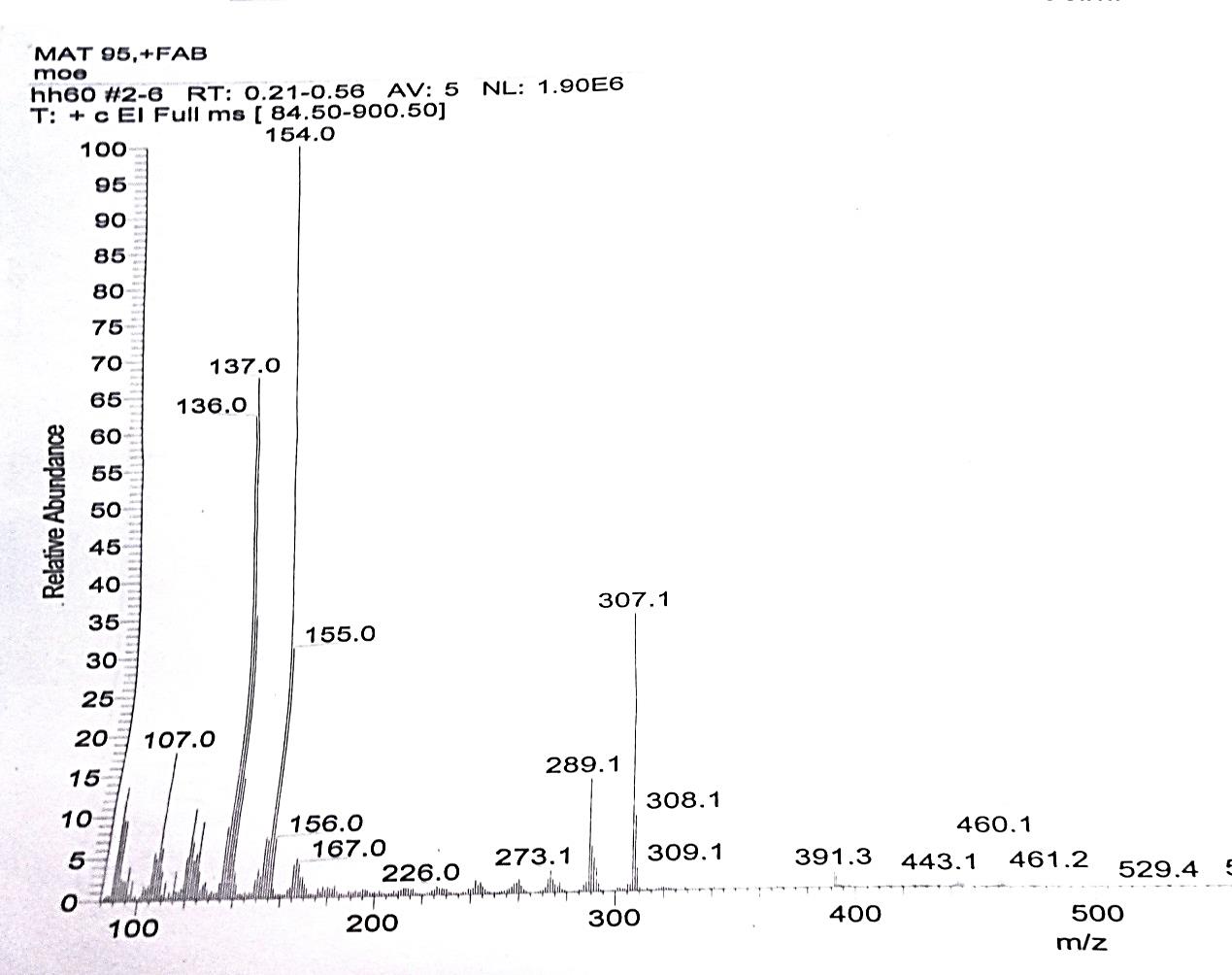
**

**S28. Mass spectrum for compound 3f**


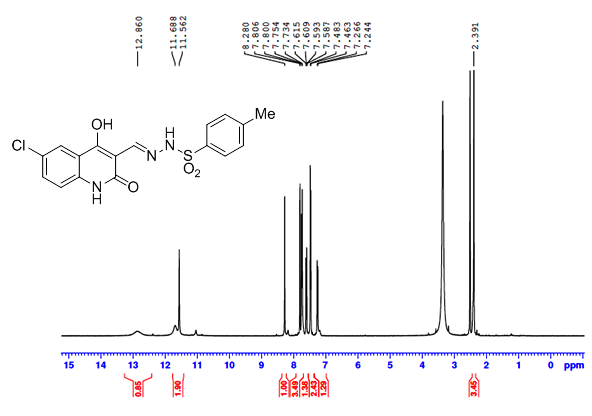


**S29.** 1H NMR Compound **3f**

**
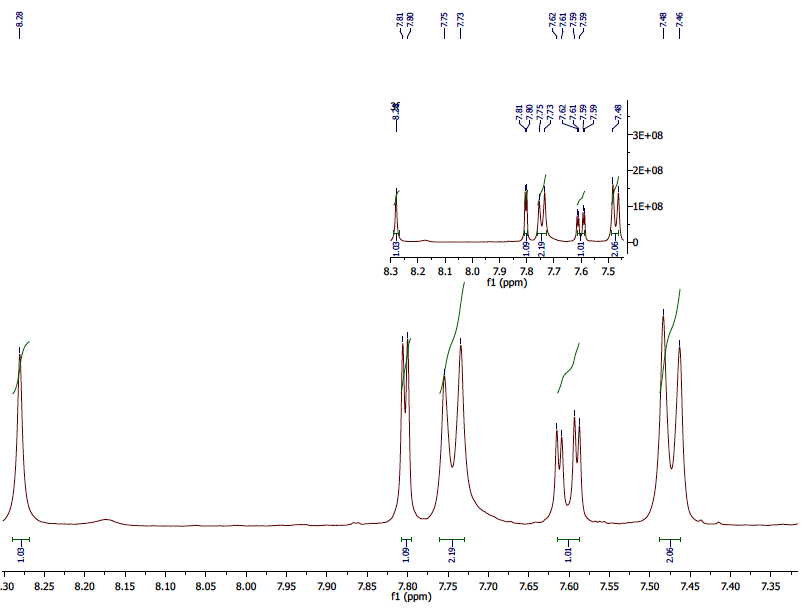
**

**S30**. Expanded 1H NMR spectrum of compound **3f**


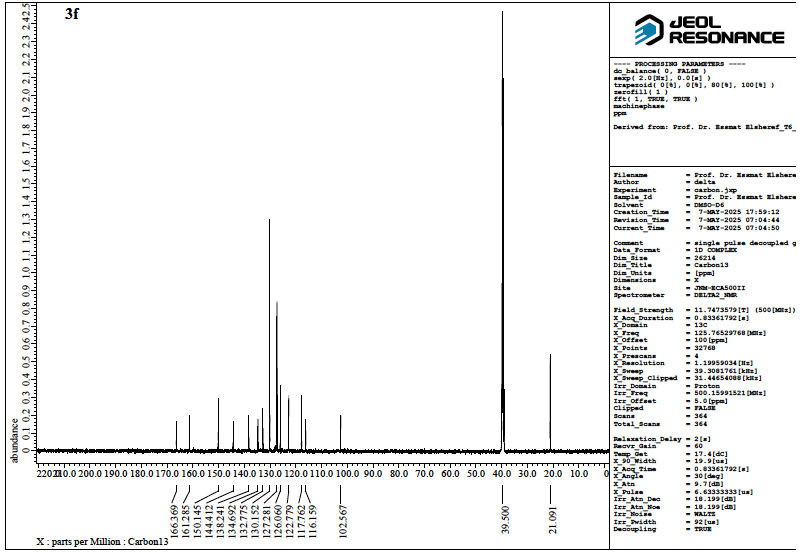


**S31.** 13C NMR Compound **3f**


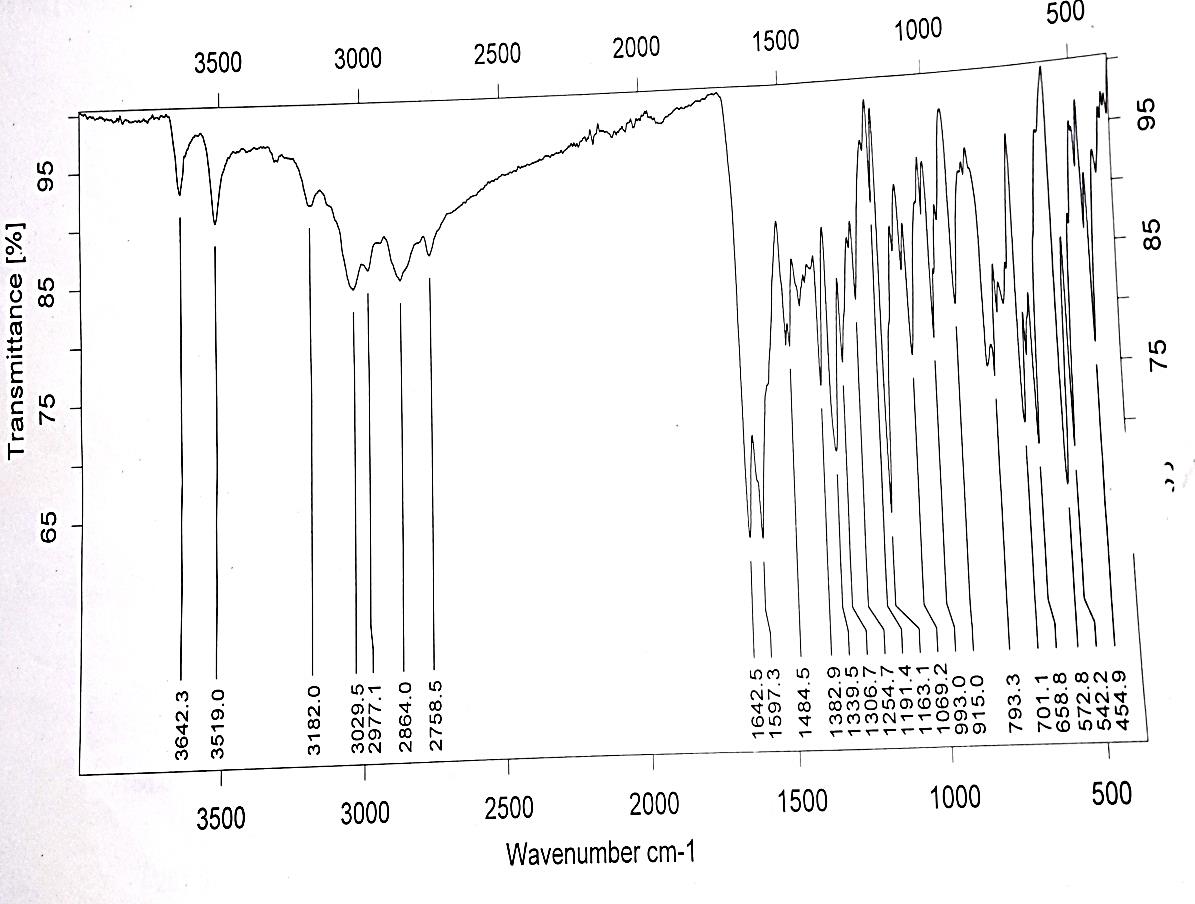


**S32. IR spectrum for compound 3g**

**
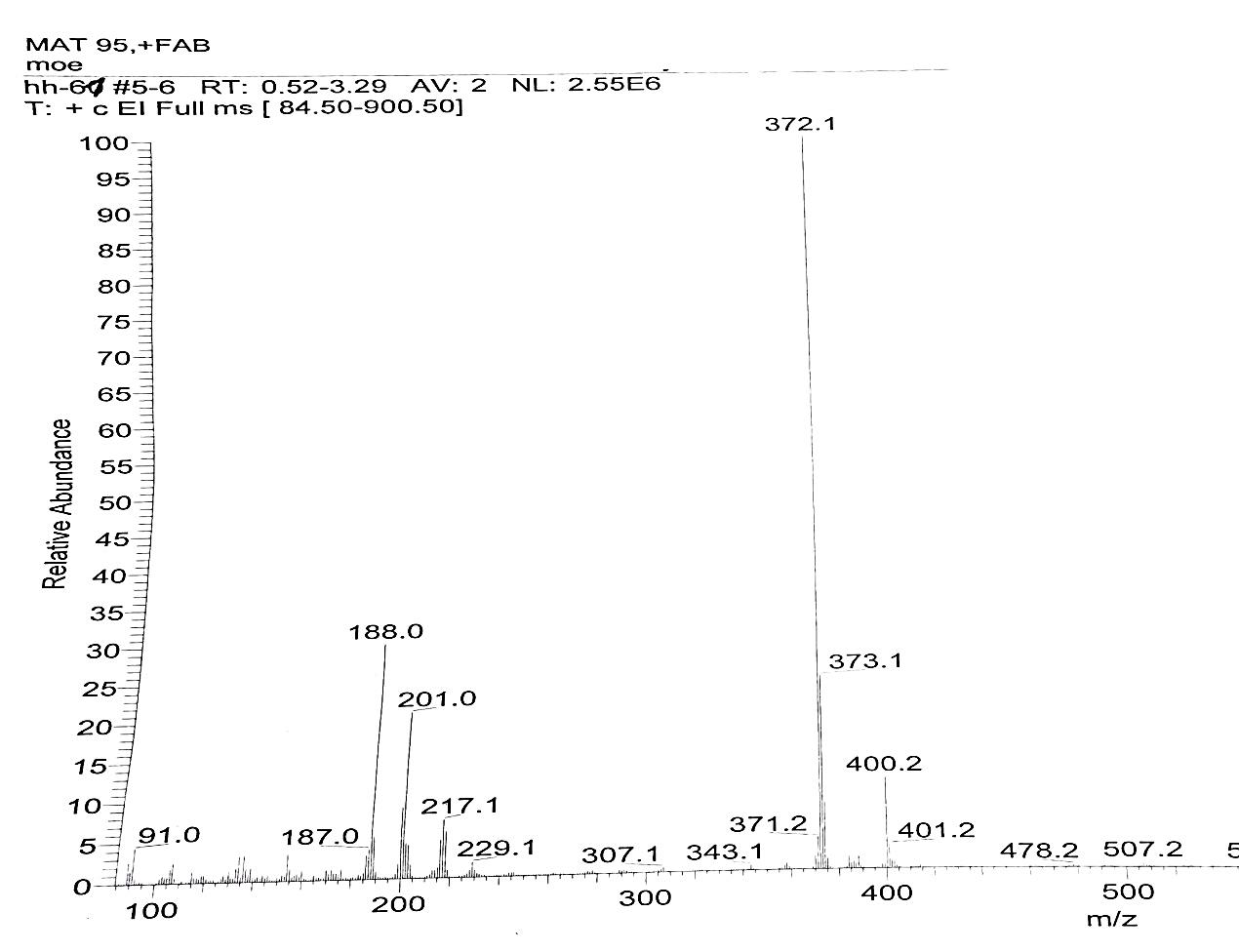
S33. Mass spectrum for compound 3g**


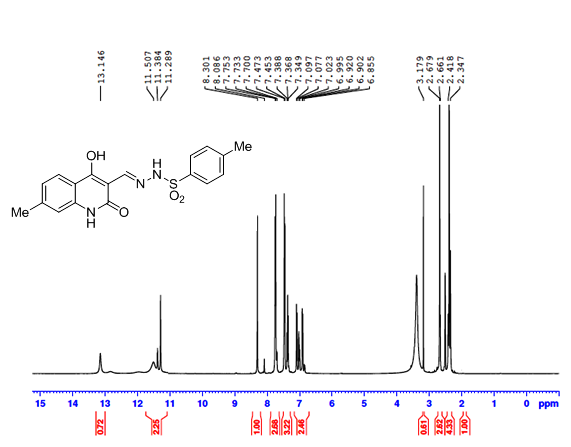


**S34.** 1H NMR Compound **3g**

**
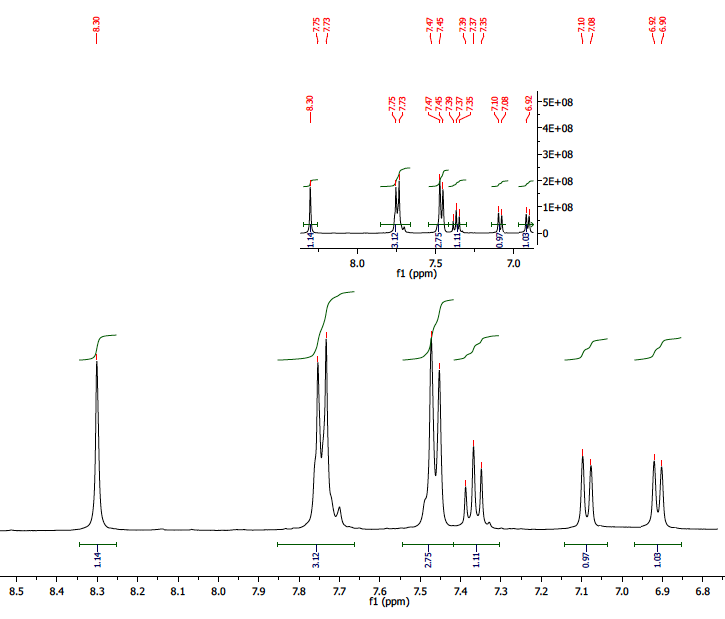
**

**S35**. Expanded 1H NMR spectrum of compound **3g**


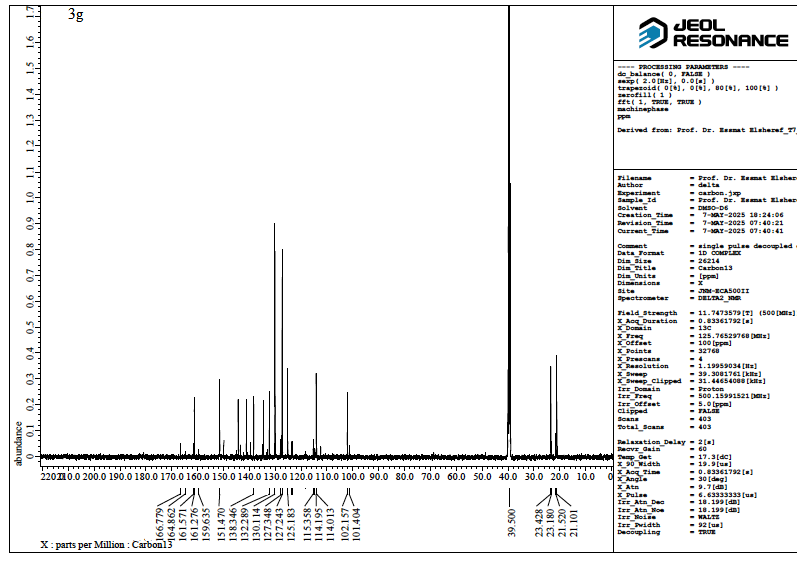


**S36.** 13C NMR Compound **3g**


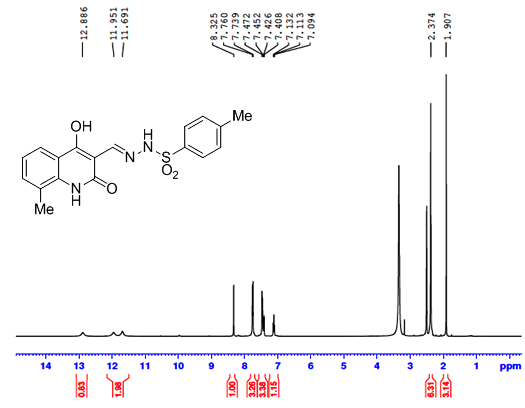


**S37.** 1H NMR Compound **3h**

**
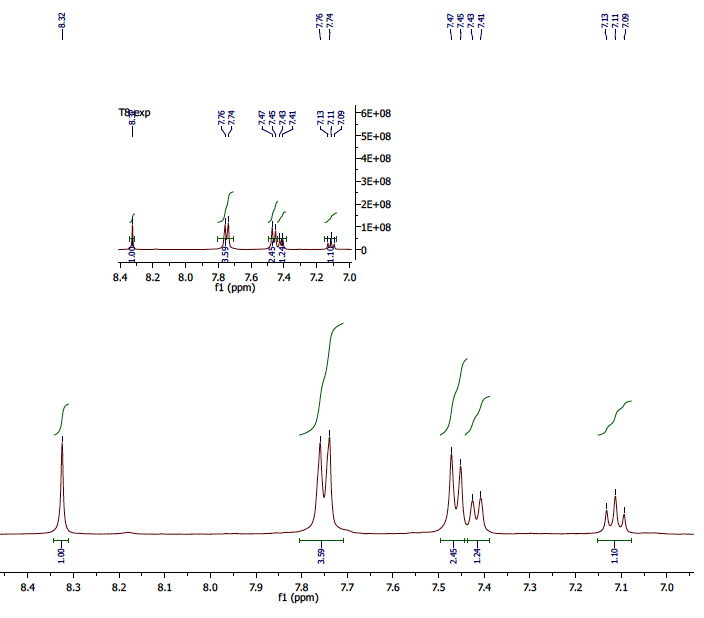
**

**S38**. Expanded 1H NMR spectrum of compound **3g**

**
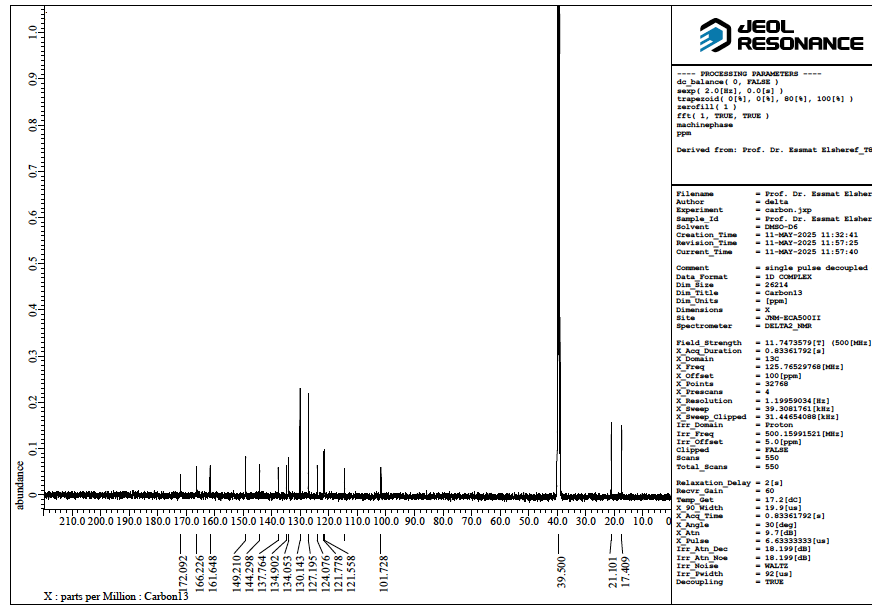
**

**S39**. Compound **3h** expansion of aromatic region

**
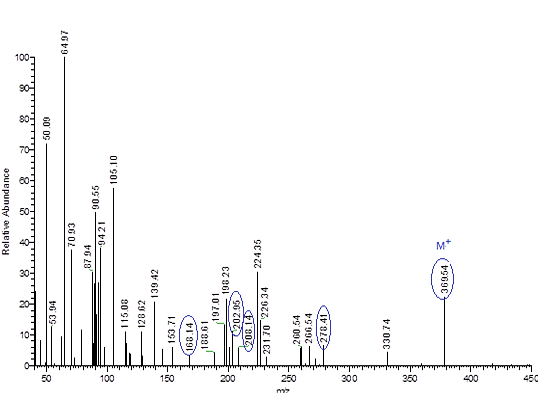
**

**S40.** Mass Spectrometry for compound **5a**

**
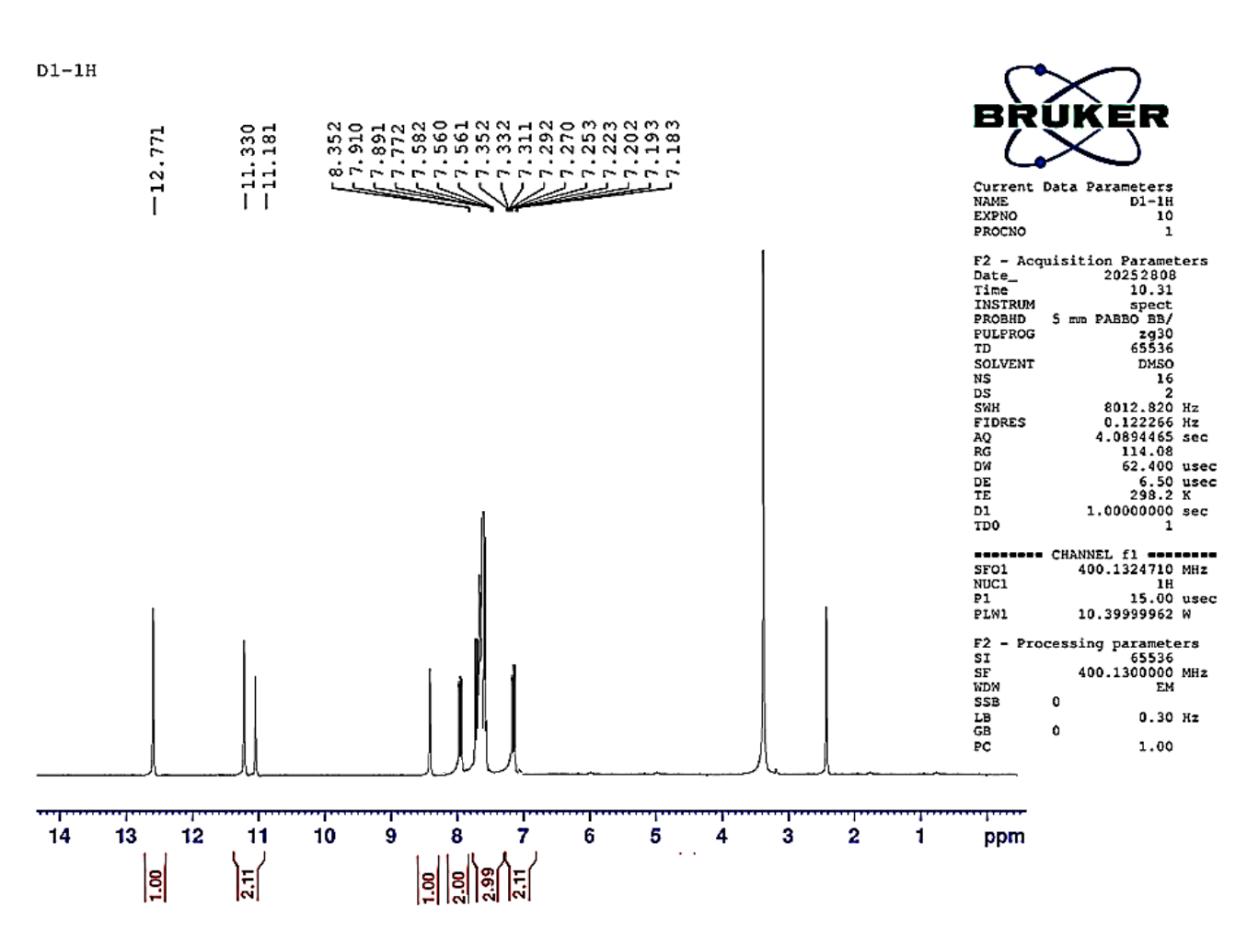
**

**S41.** 1H NMR Compound **5a**

**
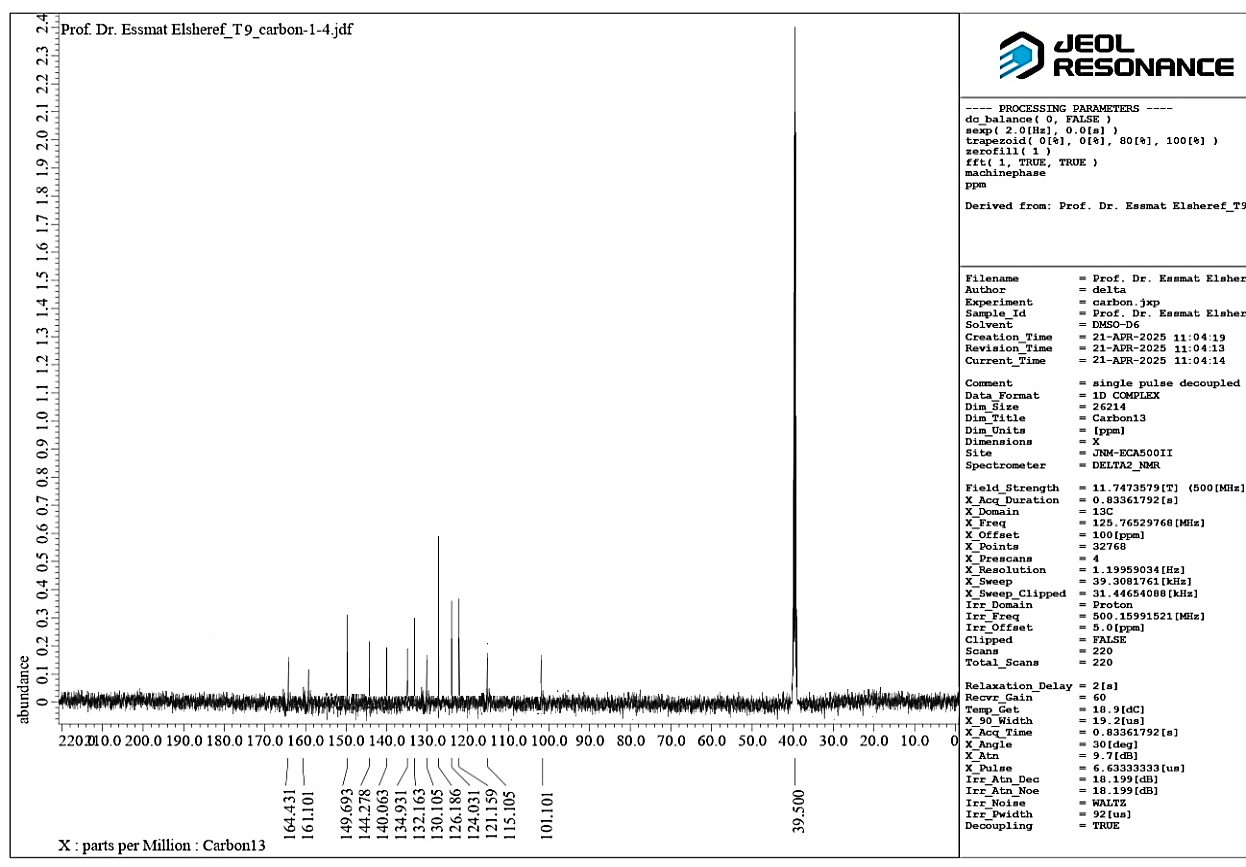
**

**S42.** 13C NMR Compound **5a**


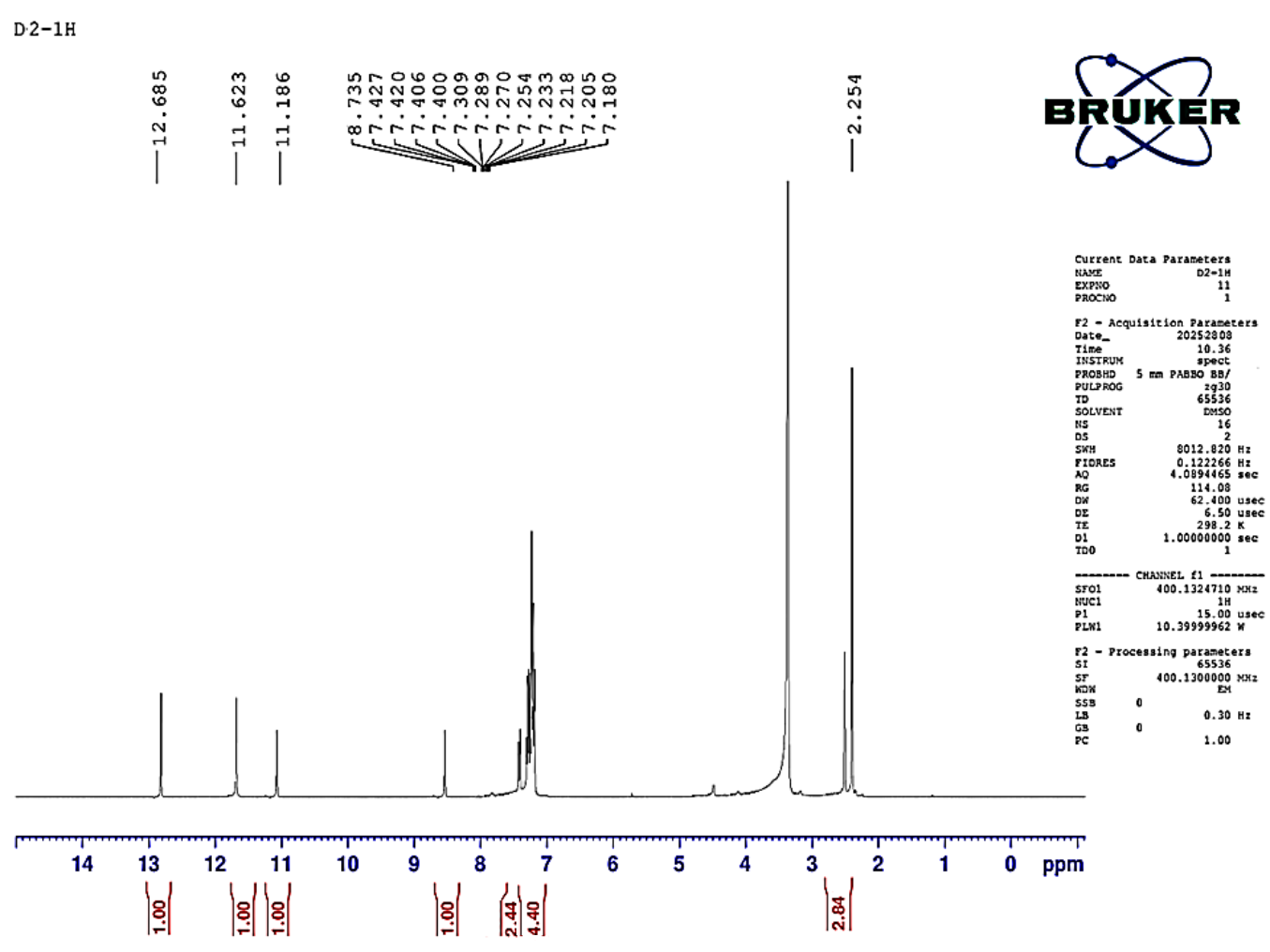


**S43.** 1H NMR Compound **5**b


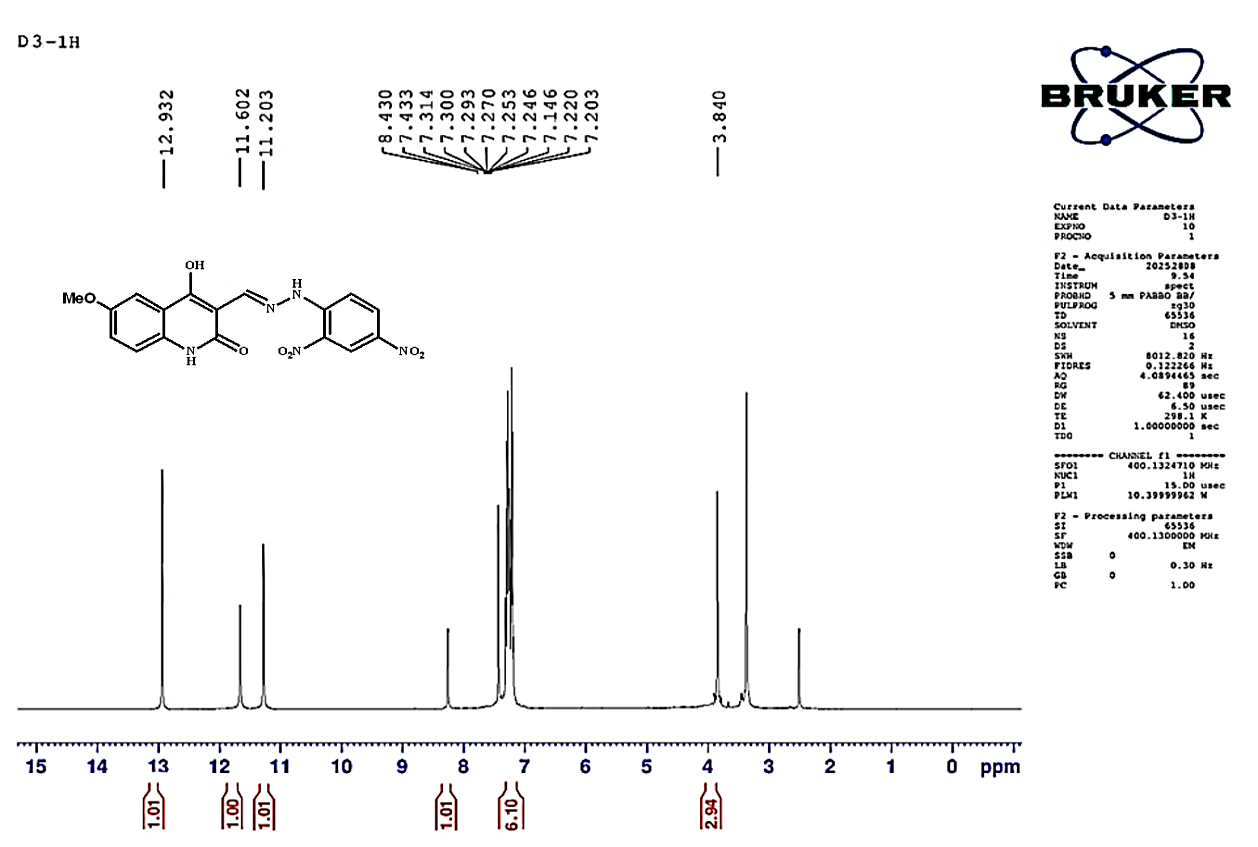


**S44.** 1H NMR Compound **5c**


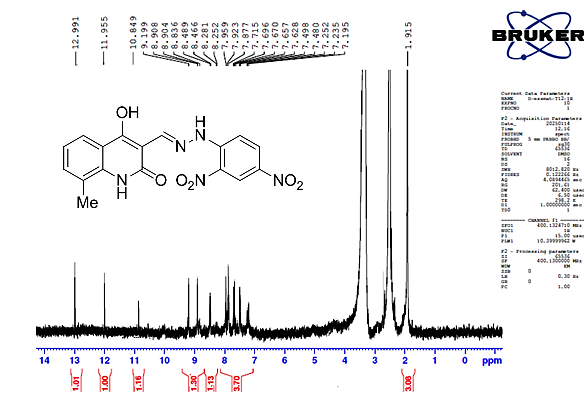


**S45.** 1H NMR Compound **5d**


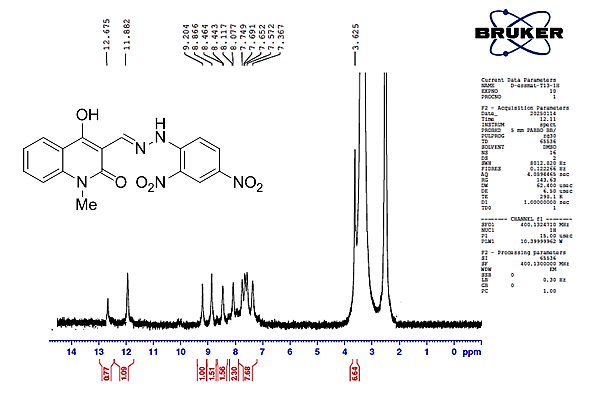


**S46.** 1H NMR Compound **5e**


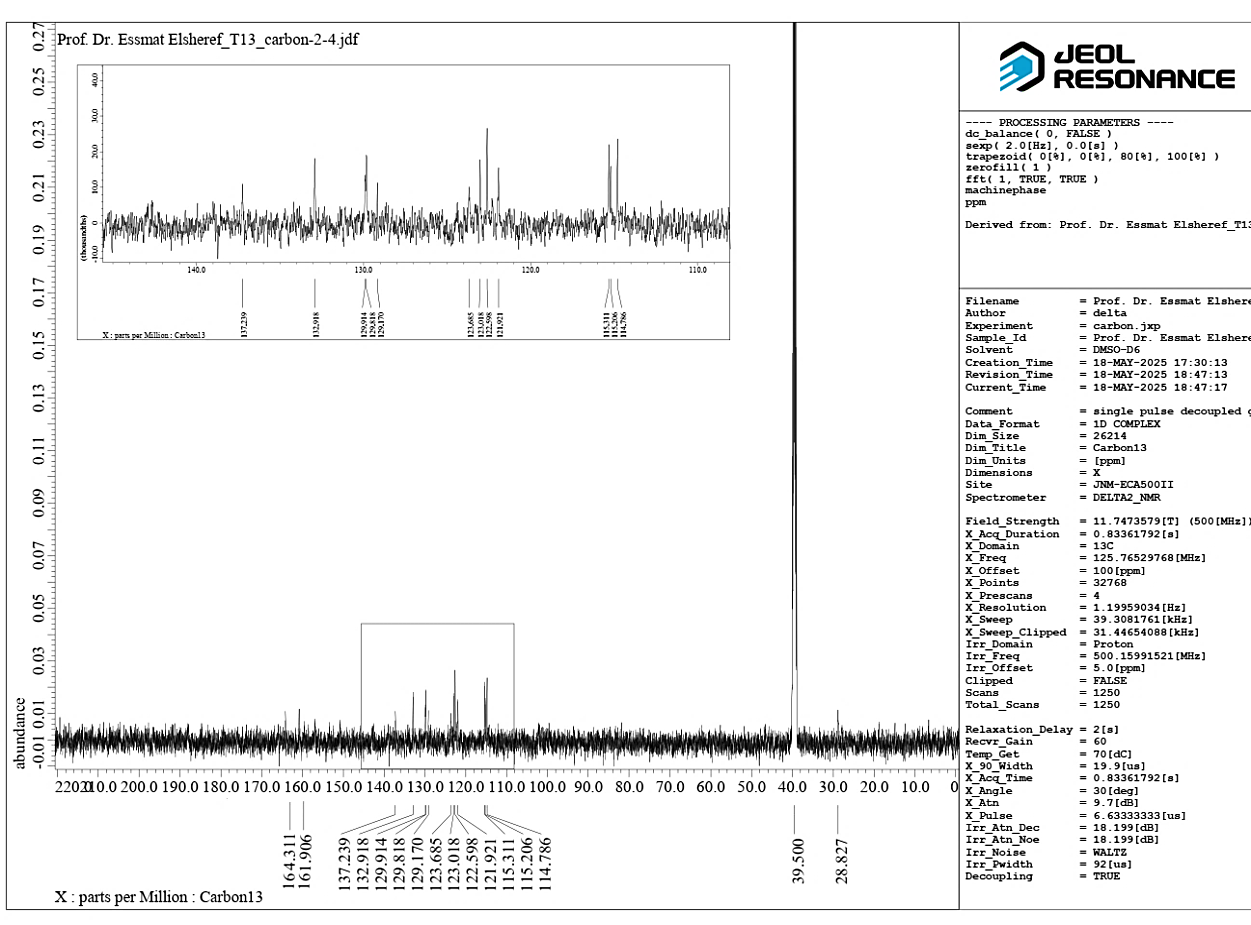


**S47.** 13C NMR Compound **5e**


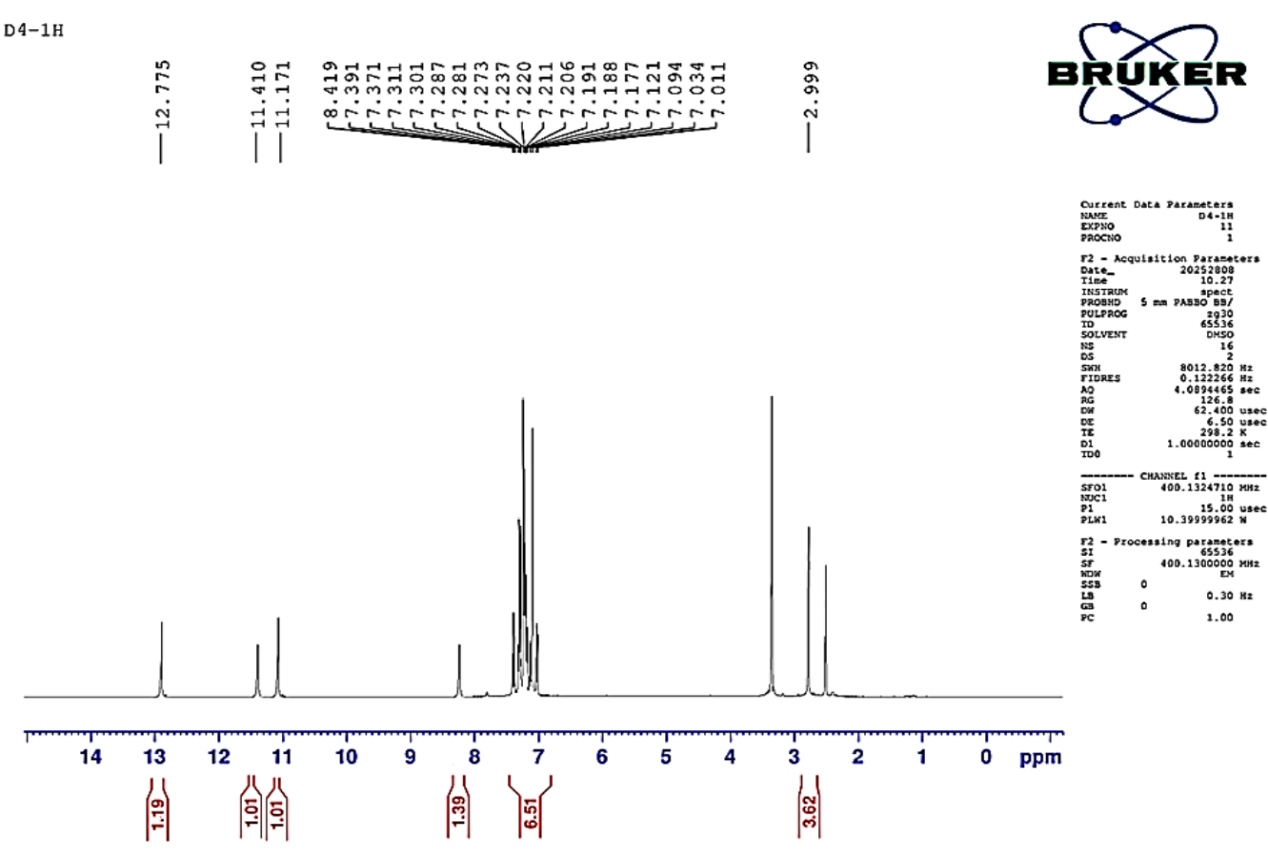


**S48.** 1H NMR Compound **5f**


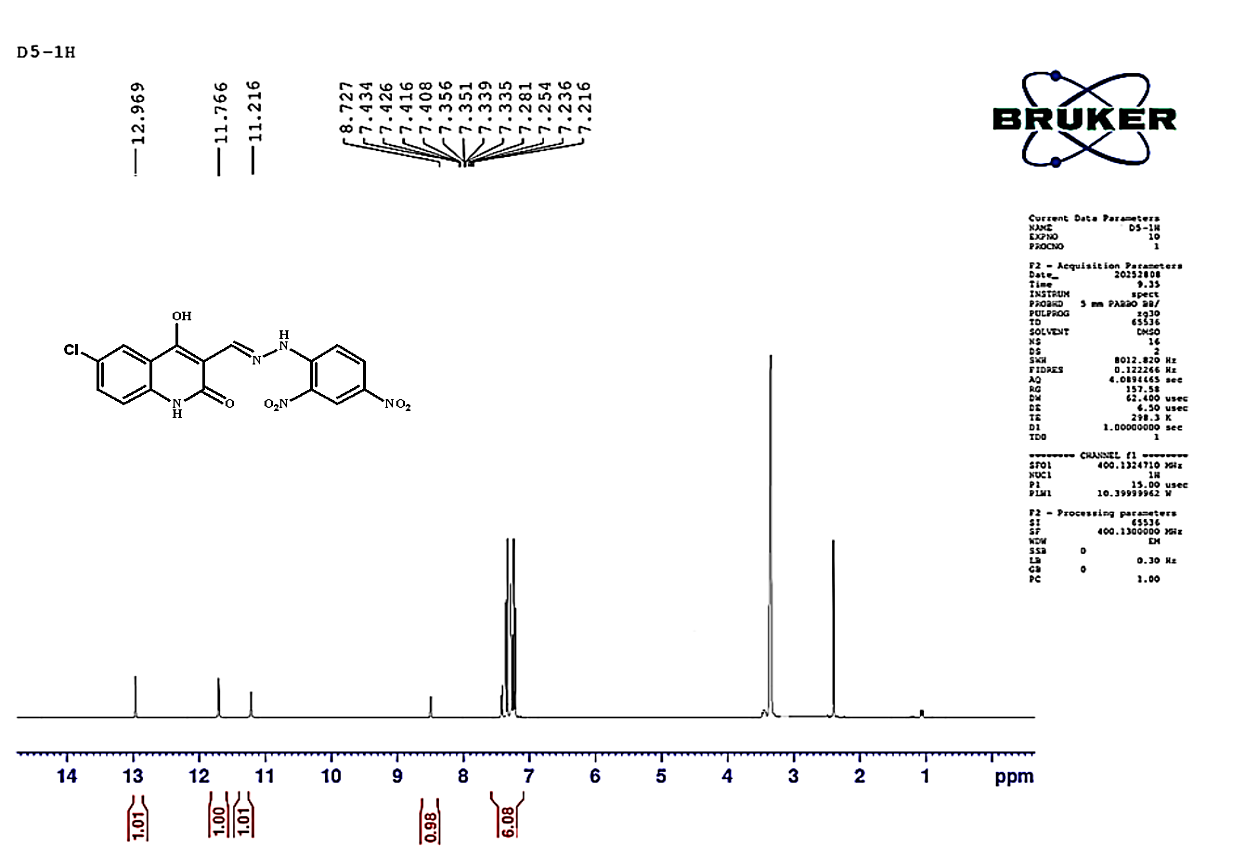


**S49.** 1H NMR Compound **5g**

**Appendix A**

**4. Experimental**

**4.1. Chemistry**

**General details**

All melting points were determined on Stuart electrothermal melting point apparatus and were uncorrected. All reactions were monitored with thin-layer chromatography (TLC) on Merck alumina-backed TLC plates and visualized under UV light. NMR spectra were measured using a Bruker AV-500 spectrometer), El Mansoura University. Also, using a (Bruker AV-400 spectrometer), Zagazig University. Chemical shifts are expressed in δ (ppm) versus internal Tetramethylsilane (TMS) = 0 ppm for 1H and 13C. The chemical shifts (δ) are reported in parts per million (ppm) relative to Tetramethylsilane (TMS) as internal standard, and the coupling constants (J) are reported in Hertz (Hz). Splitting patterns are denoted as follows: singlet (s), doublet (d), multiplet (m), triplet (t), quartet (q), doublet of doublets (dd), doublet of triplets (dt), a triplet of doublets (td), and doublet of a quartet (dq). Mass spectra were recorded on a Finnigan Fab 70 eV at Al-Azhar University, Egypt. Elemental analyses were carried out on a Perkin Elmer device at the Microanalytical Institute of Organic Chemistry, Karlsruhe Institute of Technology, Karlsruhe, Germany

**4.2. Biological evaluation**

**4.2.1 Cell Viability assay (MTT assay)**

MTT assay was performed to investigate the effect of the synthesized compounds on mammary epithelial cells (MCF-10A). The cells were propagated in medium consisting of Ham's F-12 medium/ Dulbecco's modified Eagle's medium (DMEM) (1:1) supplemented with 10% foetal calf serum, 2 mM glutamine, insulin (10 μg/mL), hydrocortisone (500 ng/mL) and epidermal growth factor (20 ng/mL). Trypsin ethylenediamine tetra acetic acid (EDTA) was used to passage the cells after every 2-3 days. 96-well flat-bottomed cell culture plates were used to seed the cells at a density of 104 cells mL-1. The medium was aspirated from all the wells of culture plates after 24 h followed by the addition of synthesized compounds (in 200 μL medium to yield a final concentration of 0.1% (v/v) dimethyl sulfoxide) into individual wells of the plates. Four wells were designated to a single compound. The plates were allowed to incubate at 37°C for 96 h. Afterwards, the medium was aspirated and 3-[4,5-dimethylthiazol-2-yl]-2,5-diphenyltetrazolium bromide (MTT) (0.4 mg/mL) in medium was added to each well and subsequently incubated for 3 h. The medium was aspirated and 150 μL dimethyl sulfoxide (DMSO) was added to each well. The plates were vortexed followed by the measurement of absorbance at 540 nm on a microplate reader. The results were presented as inhibition (%) of proliferation in contrast to controls comprising 0.1% DMSO.

**4.2.2. Assay for antiproliferative effect**

To explore the antiproliferative potential of compounds propidium iodide fluorescence assay was performed using different cell lines such as Panc-1 (pancreas cancer cell line), MCF-7 (breast cancer cell line), HT-29 (colon cancer cell line) and A-549 (epithelial cancer cell line), respectively. To calculate the total nuclear DNA, a fluorescent dye (propidium iodide, PI) is used which can attach to the DNA, thus offering a quick and precise technique. PI cannot pass through the cell membrane and its signal intensity can be considered as directly proportional to quantity of cellular DNA. Cells whose cell membranes are damaged or have changed permeability are counted as dead ones. The assay was performed by seeding the cells of different cell lines at a density of 3000-7500 cells/well (in 200μl medium) in culture plates followed by incubation for 24h at 37 °C in humidified 5%CO2/95% air atmospheric conditions. The medium was removed; the compounds were added to the plates at 10 μM concentrations (in 0.1% DMSO) in triplicates, followed by incubation for 48h. DMSO (0.1%) was used as control. After incubation, medium was removed followed by the addition of PI (25 μl, 50μg/mL in water/medium) to each well of the plates. At -80 °C, the plates were allowed to freeze for 24 h, followed by thawing at 25 oC. A fluorometer (Polar-Star BMG Tech) was used to record the readings at excitation and emission wavelengths of 530 and 620 nm for each well. The percentage cytotoxicity of compounds was calculated using the following formula:

Where A*TC*= Absorbance of treated cells and AC= Absorbance of control. Erlotinib was used as positive control in the assay.

**4.2.3. EGFR inhibitory assay**

Baculoviral expression vectors including pBlueBacHis2B and pFASTBacHTc were used separately to clone 1.6 kb cDNA coding for EGFR cytoplasmic domain (EGFR-CD, amino acids 645–1186). 5ʹ upstream to the EGFR sequence comprised a sequence that encoded (His)6. Sf-9 cells were infected for 72h for protein expression. The pellets of Sf-9 cells were solubilized in a buffer containing sodium vanadate (100 μM), aprotinin (10 μg/mL), triton (1%), HEPES buffer(50mM), ammonium molybdate (10 μM), benzamidine HCl (16 μg/mL), NaCl (10 mM),leupeptin (10 μg/mL) and pepstatin (10 μg/mL) at 0°C for 20 min at pH 7.4, followed by centrifugation for 20 min. To eliminate the nonspecifically bound material, a Ni-NTA super flow packed column was used to pass through and wash the crude extract supernatant first with 10mM and then with 100 mM imidazole. Histidine-linked proteins were first eluted with 250 and then with 500 mM imidazole subsequent to dialysis against NaCl (50 mM), HEPES (20 mM), glycerol (10%) and 1 μg/mL each of aprotinin, leupeptin and pepstatin for 120 min. The purification was performed either at 4 °C or on ice. To record autophosphorylation level, EGFR kinase assay was carried out on the basis of DELFIA/Time-Resolved Fluorometry. The compounds were first dissolved in DMSO absolute, subsequent to dilution to appropriate concentration using HEPES (25 mM) at pH 7.4. Each compound (10 μL) was incubated with recombinant enzyme (10 μL, 5 ng for EGFR, 1:80 dilution in 100 mM HEPES) for 10 min at 25oC, subsequent to the addition of 5X buffer (10 μL, containing 2 mM MnCl2, 100 μM Na3VO4, 20 mM HEPES and 1 mM DTT) and ATP-MgCl2 (20 μL, containing 0.1 mM ATP and 50 mM MgCl2) and incubation for 1h. The negative and positive controls were included in each plate by the incubation of enzyme either with or without ATP-MgCl2. The liquid was removed after incubation and the plates were washed thrice using a wash buffer. The Europium-tagged antiphosphotyrosine antibody (75 μL, 400 ng) was added to each well followed by incubation of 1h and then washing of the plates using buffer. The enhancement solution was added to each well and the signal was recorded at excitation and emission wavelengths of 340 at 615 nm. The autophosphorylation percentage inhibition by compounds was calculated using the following equation:

Using the curves of percentage inhibition of eight concentrations of each compound, IC50 was calculated. The majority of signals detected by antiphosphotyrosine antibody were from EGFR because the enzyme preparation contained low impurities.

**4.2.4. HER-2 inhibitory assay**

The ADP-Glo™ Kinase Assay was used for kinase activity detection, related kinases information: HER-2 (ab60866, Abcam), with a concentration of 100 ng/mL. Target compounds were dissolved in DMSO to obtain drug solutions with different concentrations, and the compound concentrations in the final reaction system (100 μL) were 1 nM, 20 nM, 40 nM, 80 nM, and 100 nM, respectively. 10μL of the solution containing compounds was transferred to a 96-well plate, then added 40 μL of 1 × kinase buffer (50 mM HEPES, pH 7.5) to each well, and mixed the mixture in the 96-well plate on a shaker for 10 min. Next, added 25 μL of ADP-Glo™ reagent to the above wells, mixed and incubated for another 40 min. Distributed 10 μL kinase detection reagent to the reaction and incubated for 30 min. Finally, the full-wavelength microplate reader was used to record the OD value.

**4.2.5. Caspase-3 activation assay**

Allow all reagents to reach room temperature before use. Gently mix all liquid reagents prior to use. Determine the number of 8-well strips needed for the assay. Insert these in the frame(s) for current use. Add 100 μl of the *Standard Diluent Buffer* to the zero standard wells. Well(s) reserved for chromogen blank should be left empty. Add 100 μl of standards and controls or diluted samples to the appropriate microtiter wells. The sample dilution chosen should be optimized for each experimental system. Tap gently on side of plate to mix. Cover wells with *plate cover* and incubate for 2 hours at room temperature. Thoroughly aspirate or decant solution from wells and discard the liquid, Wash wells 4 times. Pipette 100 μl of *Caspase-3 (Active) Detection Antibod*y solution into each well except the chromogen blank(s). Tap gently on the side of the plate to mix. Cover plate with *plate cover* and incubate for 1 hour at room temperature. Thoroughly aspirate or decant solution from wells and discard the liquid, Wash wells 4 times. Add 100 μl Anti-Rabbit IgG HRP Working Solution to each well except the chromogen blank(s). Prepare the working dilution as described in Preparing IgG HRP. Cover wells with the *plate cover* and incubate for 30 minutes at room temperature. Thoroughly aspirate or decant solution from wells and discard the liquid. Wash wells 4 times. Add 100 μl of *Stabilized Chromogen* to each well. The liquid in the wells will begin to turn blue. Incubate for 30 minutes at room temperature and in the dark. The incubation time for chromogen substrate is often determined by the microtiter plate reader used. Many plate readers have the capacity to record a maximum optical density (O.D.) of 2.0. The O.D. values should be monitored, and the substrate reaction stopped before the O.D. of the positive wells exceeds the limits of the instrument. The O.D. values at 450 nm can only be read after the *Stop Solution* has been added to each well. If using a reader that records only to 2.0 O.D., stopping the assay after 20 to 25 minutes is suggested. Add 100 μl of *Stop Solution* to each well. Tap side of plate gently to mix. The solution in the wells should change from blue to yellow. Read the absorbance of each well at 450 nm having blanked the plate reader against a chromogen blank composed of 100 μl each of *Stabilized Chromogen* and *Stop Solution*. Read the plate within 2 hours after adding the *Stop Solution*. Use a curve fitting software to generate the standard curve. A four-parameter algorithm provides the best standard curve fit. Read the concentrations for unknown samples and controls from the standard curve. Multiply value(s) obtained for sample(s) by the appropriate dilution factor to correct for the dilution in step 3. Samples producing signals greater than that of the highest standard should be diluted in *Standard Diluent Buffer* and reanalyzed.

**4.2.6. Caspase-8/9 activation assay**

Cells were obtained from American Type Culture Collection, cells were grown in RPMI 1640 containing 10% fetal bovine serum at 37°C, stimulated with the compounds to be tested for caspase8, and lysed with Cell Extraction Buffer. This lysate was diluted in Standard Diluent Buffer over the range of the assay and measured for human active caspase-8 content. (*Cells are Plated in a density of 1.2 – 1.8 × 10,000 cells/well in a volume of 100µl complete growth medium + 100 ul of the tested compound per well in a 96-well plate for 24 hours before the enzyme assay for Tubulin*.). The absorbance of each microwell was read on a spectro-photometer at 450 nm. A standard curve is prepared from 7human Caspase-8 standard dilutions and human Caspase-8 concentration determined.

**4.2.7. Bax activation assay**

Bring all reagents, except the human Bax-α Standard, to room temperature for at least 30 minutes prior to opening. The human Bax-α Standard solution should not be left at room temperature for more than 10 minutes. All standards, controls and samples should be run in duplicate. Refer to the Assay Layout Sheet to determine the number of wells to be used and put any remaining wells with the desiccant back into the pouch and seal the ziploc. Store unused wells at 4 °C. Pipet 100 μL of Assay Buffer into the S0 (0 pg/mL standard) wells. Pipet 100 μL of Standards #1 through #6 into the appropriate wells. Pipet 100 μL of the Samples into the appropriate wells. Tap the plate gently to mix the contents. Seal the plate and incubate at room temperature on a plate shaker for 1 hour at ~500 rpm. Empty the contents of the wells and wash by adding 400 μL of wash solution to every well. Repeat the wash 4 more times for a total of **5 washes**. After the final wash, empty or aspirate the wells and firmly tap the plate on a lint free paper towel to remove any remaining wash buffer. Pipet 100 μL of yellow Antibody into each well, except the Blank. Seal the plate and incubate at room temperature on a plate shaker for 1 hour at ~500 rpm. Empty the contents of the wells and wash by adding 400 μL of wash solution to every well. Repeat the wash 4 more times for a total of **5** washes. After the final wash, empty or aspirate the wells and firmly tap the plate on a lint free paper towel to remove any remaining wash buffer. Add 100 μL of blue Conjugate to each well, except the Blank. Seal the plate and incubate at room temperature on a plate shaker for 30 minutes at ~500 rpm. Empty the contents of the wells and wash by adding 400 μL of wash solution to every well. Repeat the wash 4 more times for a total of **5 washes**. After the final wash, empty or aspirate the wells and firmly tap the plate on a lint free paper towel to remove any remaining wash buffer. Pipet 100 μL of Substrate Solution into each well. Incubate for 30 minutes at room temperature on a plate shaker at ~500 rpm. Pipet 100 μL Stop Solution to each well. Blank the plate reader against the Blank wells, read the optical density at 450 nm. Calculate the average net Optical Density (OD) bound for each standard and sample by subtracting the average Blank OD from the average OD for each standard and sample. Using linear graph paper, plot the Average Net OD for each standard versus Bax concentration in each standard. Approximate a straight line through the points. The concentration of Bax in the unknowns can be determined by interpolation.

**4.2.8. Bcl-2 inhibition assay**

Mix all reagents thoroughly without foaming before use. Wash the microwells twice with approximately 300 μL Wash Buffer per well with thorough aspiration of microwell contents between washes. Take caution not to scratch the surface of the microwells. After the last wash, empty the wells and tap microwell strips on absorbent pad or paper towel to remove excess Wash Buffer. Use the microwell strips immediately after washing or place upside down on a wet absorbent paper for not longer than 15 minutes. Do not allow wells to dry. Add 100 μL of Sample Diluent in duplicate to all standard wells and to the blank wells. Prepare standard (1:2 dilution) in duplicate ranging from 32 ng/mL to 0.5 ng/mL. Add 100 μL of Sample Diluent, in duplicate, to the blank wells. Add 80 μL of Sample Diluent, in duplicate, to the sample wells. Add 20 μL of each Sample, in duplicate, to the designated wells. Add 50 μL of diluted biotin-conjugate to all wells, including the blank wells. Cover with a plate cover and incubate at room temperature, on a microplate shaker at 100 rpm if available, for 2 hours. Remove plate cover and empty the wells. Wash microwell strips 3 times as described in step 2. Add 100 μL of diluted Streptavidin-HRP to all wells, including the blank wells. Cover with a plate cover and incubate at room temperature, on a microplate shaker at 100 rpm if available, for 1 hour. Remove plate cover and empty the wells. Wash microwell strips 3 times as described in step 2. Proceed to the next step. Pipette 100 μl of mixed TMB Substrate Solution to all wells, including the blanks. Incubate the microwell strips at room temperature (18° to 25°C) for about 15 minutes, if available on a rotator set at 100 rpm. Avoid direct exposure to intense light. The point, at which the substrate reaction is stopped, is often determined by the ELISA reader. Many ELISA readers record absorbance only up to 2.0 O.D. Therefore, the color development within individual microwells must be watched by the person running the assay and the substrate reaction stopped before positive wells are no longer properly detectable. Stop the enzyme reaction by quickly pipetting 100 μL of Stop Solution into each well, including the blank wells. It is important that the Stop Solution is spread quickly and uniformly throughout the microwells to completely inactivate the enzyme. Results must be read immediately after the Stop Solution is added or within one hour if the microwell strips are stored at 2 - 8°C in the dark. Read absorbance of each microwell on a spectrophotometer using 450 nm as the primary wavelength.

**4.2.9. Cytochrome C assay**

Cells were obtained from American Type Culture Collection, cells were grown in RPMI 1640 containing 10% fetal bovine serum at 37°C, stimulated with the compounds to be tested for cytochrome C, and lysed with Cell Extraction Buffer. This lysate was diluted in Standard Diluent Buffer over the range of the assay and measured for cytochrome C content. (Cells are Plated in cells/well in a volume of 100µl complete growth medium + 100 µl of the tested compound + 50 µl of 1X biotin conjugated antibody+ 100 µl of 1X streptavidin-HRP+ 100 µL TMB substrate solution of per well in a 96-well plate for 24 hours before assay).

**4.2.10. Cell apoptosis assay**

Apoptosis was determined by flow cytometry based on the Annexin-V-fluoresce in isothiocyanate (FITC) and propidium iodide (PI) staining kit (BD Pharmingen, San Diego, USA). Apoptosis was determined by flow cytometry based on the Annexin-V-fluoresce in isothiocyanate (FITC) and propidium iodide (PI) staining kit (BD Pharmingen, San Diego, USA). Apoptotic cells were defined as Annexin-V-positive. Cells were grown to approximately ∼70% confluence and exposed to different concentrations of compounds (0, 2, 4, 6 and8 μmol/L) for 24 h. Treated cells were trypsinzed, washed twice with PBS and transferred into micro centrifuge tubes for centrifugation at1000 rpm for 5 min at room temperature, then resuspended in binding buffer, 5 μL of FITC and PI were added to Eppendorf tube, cells were vortexed, incubated for 15 min at room temperature in dark. Subsequently, cells were analyzed by flow cytometry (Becton Dickinson, Franklin Lakes, and USA).

**4.3. Docking Studies**

Molecular docking simulations were performed to investigate the interactions between the synthesized quinazoline/1,3,4-oxadiazole derivatives and key cancer-related proteins, including EGFR and HER2, using Discovery Studio software. The protein structures were prepared and optimized for docking analysis, retaining only the essential chains for accurate binding studies.

**4.4. Molecular dynamic simulations**

A molecular dynamics (MD) simulation was conducted using GROMACS 2023 to validate the docking results and assess the stability of the interactions between the synthesized quinazoline/1,3,4-oxadiazole derivatives and the target HER2.

The protein structures were prepared using UCSF Chimera, which included the addition of hydrogen atoms to ensure accurate geometry. The CHARMM36 force field was applied to proteins, while the CGenFF force field was used for ligands. The protein-ligand complexes were immersed in a TIP3P water box with a 1 nm buffer zone to ensure proper hydration. Sodium chloride ions were added to neutralize the system, adjusting the concentration to 150 mM. Energy minimization was performed using the steepest descent method, followed by a two-phase equilibration process: a 100 ps NVT (constant number of particles, volume, and temperature) phase and a 100 ps NPT (constant number of particles, pressure, and temperature) phase at 300 K and 1.0 bar, with position restraints applied to the protein-ligand complex. A 100-ns production run was then conducted without restraints, during which trajectories were recorded every 10 ps for subsequent analysis. Key parameters, including root mean square deviation (RMSD) and binding energy, were calculated to evaluate the stability and dynamics of the protein-ligand interactions.

**Computational Methodology (DFT Studies)**

Density Functional Theory (DFT) calculations were conducted to analyze the electronic structure, geometry, and reactivity descriptors of compound **5a**. All calculations were performed using the Gaussian 09 suite of programs, with Gauss View 6.0 employed for molecular visualization and orbital analysis. The geometry of compound 5a was fully optimized using the Becke, 3-parameter, Lee–Yang–Parr (B3LYP) hybrid functional in combination with the 6-311+G(d,p) basis set, which includes both diffuse and polarization functions to account for electron delocalization and molecular interactions.

**4.3. Statistical analysis**

Computerized Prism 5 program was used to statistically analyzed data using one-way ANOVA test followed by Tukey’s as post ANOVA for multiple comparison at P ≤.05. Data were presented asmean ± SEM
